# Genome and Transcriptome Analyses of Genes Involved in Ascorbate Biosynthesis in Pepper Indicate Key Genes Related to Fruit Development, Stresses, and Phytohormone Exposures

**DOI:** 10.3390/plants12193367

**Published:** 2023-09-23

**Authors:** Évelyn Silva de Aguiar, Abigailde Nascimento Dias, Raquel Mendes Sousa, Thais Andrade Germano, Renato Oliveira de Sousa, Rafael de Souza Miranda, José Hélio Costa, Clesivan Pereira dos Santos

**Affiliations:** 1 Postgraduate Program in Environmental Sciences, Center of Sciences of Chapadinha, Federal University of Maranhão, Boa Vista, Chapadinha 65500-000, Maranhão, Brazil; evelynsaguiar@gmail.com; 2 Center of Sciences of Chapadinha, Federal University of Maranhão, Boa Vista, Chapadinha 65500-000, Maranhão, Brazil; abigaildedias2001@gmail.com (A.N.D.); mendesraquel332@gmail.com (R.M.S.); 3 Department of Biochemistry and Molecular Biology, Federal University of Ceará, Fortaleza 60451-970, Ceará, Brazil; tandradeg@gmail.com (T.A.G.); helio.costa@ufc.br (J.H.C.); 4 Postgraduate Program in Agricultural Sciences, Campus Professora Cinobelina Elvas, Federal University of Piauí, Bom Jesus 64900-000, Piauí, Brazil; renato.sousa@ufpi.edu.br (R.O.d.S.); rsmiranda@ufpi.edu.br (R.d.S.M.); 5 Plant Science Department, Federal University of Piauí, Teresina 64049-550, Piauí, Brazil

**Keywords:** acid ascorbic, *Capsicum annuum*, metabolism regulation, gene expression, environmental stresses

## Abstract

Pepper (*Capsicum annuum* L.) is a vegetable consumed worldwide, primarily used for vitamin C uptake and condiment purposes. Ascorbate (Asc) is a multifunctional metabolite, acting as an antioxidant and enzymatic cofactor involved in multiple cellular processes. Nevertheless, there is no evidence about the contribution of biosynthesis pathways and regulatory mechanisms responsible for Asc reserves in pepper plants. Here, we present a genome- and transcriptome-wide investigation of genes responsible for Asc biosynthesis in pepper during fruit development, stresses, and phytohormone exposures. A total of 21 genes, scattered in ten of twelve pepper chromosomes were annotated. Gene expression analyses of nine transcriptomic experiments supported the primary role of the L-galactose pathway in the Asc-biosynthesizing process, given its constitutive, ubiquitous, and high expression profile observed in all studied conditions. However, genes from alternative pathways generally exhibited low expression or were unexpressed and appeared to play some secondary role under specific stress conditions and phytohormone treatments. Taken together, our findings provide a deeper spatio-temporal understanding of expression levels of genes involved in Asc biosynthesis, and they highlight *GGP2*, *GME1* and *2*, and *GalLDH* members from L-galactose pathway as promising candidates for future wet experimentation, addressing the attainment of increase in ascorbate content of peppers and other crops.

## 1. Introduction

L-ascorbate, Asc, (Vitamin C) constitutes one of the most versatile metabolites in plants and animals [1,2]. Some primates, including humans, have lost the ability to synthesize Asc due to a gene mutation that transformed the last enzyme of this pathway, L-gulone-lactone-oxidase (GulLO), into a non-functional one [3]. Thus, its supply depends on the ingestion of fruit and vegetables rich in this vitamin [4]. In animal cells, vitamin C plays a vital role, acting as a general antioxidant, cofactor of mono- and dioxygenases and absorption and cellular uptake of iron, and in the demethylation of DNA and histones [5,6]. In plants, Asc acts as a major antioxidant and enzymatic cofactor, involved in several cellular and molecular processes, such as cell division, expansion, photosynthesis, respiration, and in tolerance to environmental stresses [1,4].

Plants are sessile organisms constantly exposed to high temperatures, salinity, drought, flooding, pathogens attacks, and other environmental stressors, which challenge their survival. Under these conditions, plants trigger several biochemical and physiological responses, including increase in reactive oxygen species (ROS) production, photosynthesis unbalancing, increase in leaf temperature, photorespiration, and transpiration [7,8], impairing normal plant development and even causing death. In response to oxidative stresses, plants trigger two antioxidant mechanisms: the enzymatic system composed mainly of superoxide dismutase, catalase, and ascorbate peroxidase, and the non-enzymatic system composed mainly of ascorbate and glutathione. As an antioxidant, Asc prevents the overproduction of ROS, thereby avoiding the oxidation of DNA, proteins, lipids, and other cell structures and functioning as an essential micronutrient for rapid and efficient plant response and recovery to the stressors [9]. 

**Figure 1:**
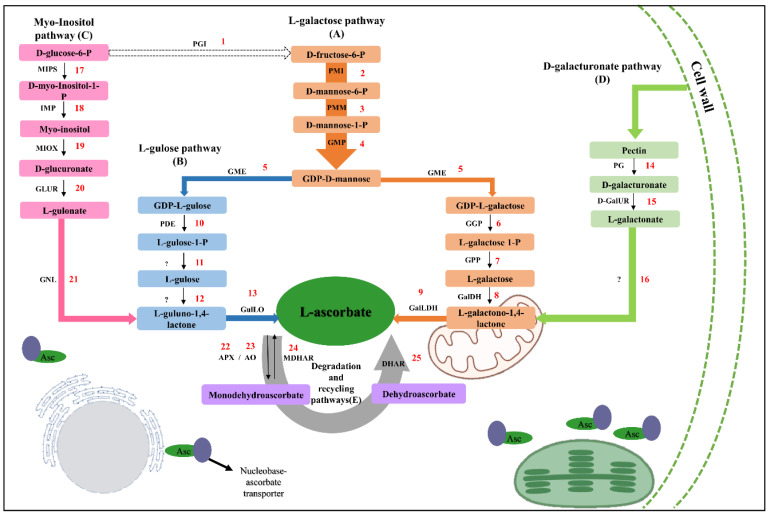
Schematic representation of ascorbate metabolism pathways in plants using the Biorender webtool (https://www.biorender.com/ (accessed on 20 July 2022)). The enzymatic steps are numbered as follows: D-mannose/L-galactose pathway (A): 1, glucose-6-phosphate isomerase (PGI); 2, phosphomannose isomerase (PMI); 3, phosphomannose mutase (PMM); 4, GDP-mannose pyrophosphorylase (GMP); 5, GDP mannose-3’,5’-epimerase (GME); 6, GDP-L-galactose transferase (GGP); 7, L-galactose-1-phosphate phosphatase (GPP); 8, L-galactose dehydrogenase (GalDH); and 9, L-galactone-1,4-lactone dehydrogenase (GalLDH). L-gulose pathway (B): 10, phosphodiesterase (PDE); 13, L-gulono-1,4-lactone oxidase (GulLO). D-galacturonate pathway (C): 14, polygalacturonase (PG) and 15, D-galacturonate reductase (GalUR). myo-inositol pathway (D): 17, L-myo-inositol-1-phosphate synthase (MIPS); 18, myo-inositol monophosphatase (IMP); 19, myo-inositol oxygenase (MIOX); 20, glucuronate reductase (GLUR); and 21, Gulono-lactonase (GNL). Degradation and recycling pathways (E): 22, ascorbate peroxidase (APX); 23, ascorbate oxidase (AO); 24, monodehydroascorbate reductase (MDHAR); and 25, dehydroascorbate reductase (DHAR). Steps 11, 12, and 16 (?) indicate unknown enzymes.

Most of the current understanding for elucidation of Asc biosynthesis was built using *Arabidopsis* and tomato models, species that possess very low Asc contents [10,11,12]. As a result, four pathways have been proposed for Asc biosynthesis in plants (Figure 1). Genetic and biochemical approaches have been employed to elucidate all steps of the D-mannose/L-galactose pathway and described as primary approaches for ascorbate synthesis [2,10,13]. Furthermore, emerging evidence indicated the presence of alternative pathways of D-galacturonate [14], L-gulose [15], and myo-inositol [11]. However, the meaningfulness of their contribution to the ascorbate pool is still quite controversial [2,16,17]. Furthermore, degradation, recycling, and transport processes are also determinants for fine-tuning of ascorbate reserves [12,13,18,19]. 

Although plants possess different pathways to biosynthesize ascorbate, studies point out that their contribution to this process varies between species, organs, and developmental stages. Analysis of the camu-camu transcriptome revealed the presence of transcripts encoding all proposed pathways [20]. Investigations in jujube and orange fruits suggested the D-mannose/L-galactose pathway was the principal pathway, assisted by the myo-inositol pathway [17,21,22]. On the other hand, the D-mannose/L-galactose pathway predominated in immature strawberry fruit, while D-galacturonate and myo-inositol pathways showed a positive correlation with Asc accumulation in ripe fruit [23], whereas the transcriptome of pineapple and acerola (fruit, leaf and flower) pointed out the only contribution of the D-mannose/L-galactose pathway [24,25], thus revealing that the L-galactose pathway is constitutively and ubiquitously operating in plants. 

Ascorbate biosynthesis reflects the genetic base of each species, being continuously adjusted according to cell compartment, plant organs, developmental stage, circadian cycle, and stress conditions [13,23]. The relevance of Asc for plant biochemical and physiological responses to abiotic and biotic stresses is becoming clear. Light is an essential signal for the activation of photosynthesis and Asc biosynthesis in *Arabidopsis* by the up-regulation of GDP-mannose pyrophosphorylase (*GMP*), GDP-Galactose phosphorylase (*GGP*), L-galactose-1-phosphate phosphatase (*GPP*), and L-galactono-1,4-lactone dehydrogenase (*GalLDH*) [26,27]. The genetic manipulation of the Asc biosynthesis process by overexpression of *GME* and *GGP* genes of the D-mannose/L-galactose pathway in *Arabidopsis* and rice leaves [28,29] and *GalLDH* in tobacco [30] increased the Asc content by 1.5 to 2.5-fold, conferring tolerance to salt and drought stresses. The overexpression of genes from alternative pathways, particularly, *MIOX4* and *GulLO2* [31] in *Arabidopsis* and *GalUR* [32] in tomato plants increased Asc by 1.6 to 3.0-fold and promoted tolerance to salt, cold, and heat.

Therefore, a better comprehension of the regulatory mechanisms responsible for Asc accumulation in horticultural crops has been a topic of interest due to its vast practical application. Increases in Asc content by genetic manipulation may improve human vitamin C uptake from diet by biofortification of the most consumed foods, increase post-harvest time of fresh fruit and vegetables, and mainly improve crops’ resilience to environmental stresses, contributing to sustainable production [6]. *Capsicum annuum* L. is one of the most consumed vegetables worldwide known as a primary source of vitamin C for human diet due to its high ascorbate content (50–250 mg 100 g^−1^ Fresh Weight) accumulated as the ripening progresses [33,34]. Pepper germplasm comprises a large group of economically important crops used as vegetables (bell peppers) and as spices for condiments (chilli peppers), displaying a wide variety of sizes, shapes, colors, flavors, and antioxidant compounds [35,36]. So far, few investigations have been performed to clarify the ascorbate metabolism in this species. These evidenced the contribution of the GDP-mannose pyrophosphorylase (*GMP*), GDP mannose-3’,5’-epimerase (*GME*) genes from the D-mannose/L-galactose pathway, and ascorbate oxidase (*AO*) from degradation process for the accumulation of ascorbate [33,34]. 

Thus, studies are scarce regarding the regulatory mechanisms of ascorbate in peppers, and there is a lack of information about the participation of the proposed biosynthesis pathways. Recently, multiple genome and transcriptome sequencing analyses have been performed in this species, and data are released in public database as GenBank-NCBI. These large-scale datasets can drive relevant advances on major biological concerns like the improvement of plant adaptation and productivity under various challenging environmental conditions. A better comprehension on ascorbate metabolism is expected to effectively contribute to future plant breeding strategies. Therefore, in this study, we present a wide investigation into diversity and expression of gene families encoding Asc biosynthesis pathways in pepper by exploring nine transcriptomic experiments related to fruit development, under stresses and phytohormone exposures. Here, we discuss the apparent contribution of principal and alternative pathways for the Asc biosynthesis process and highlight candidate markers involved in the regulation of Asc biosynthesis useful for the manipulation of Asc level in target crops.

## 2. Results

### 2.1. Identification and Characterization of Genes Encoding Ascorbate Biosynthesis Enzymes

**Figure 2:**
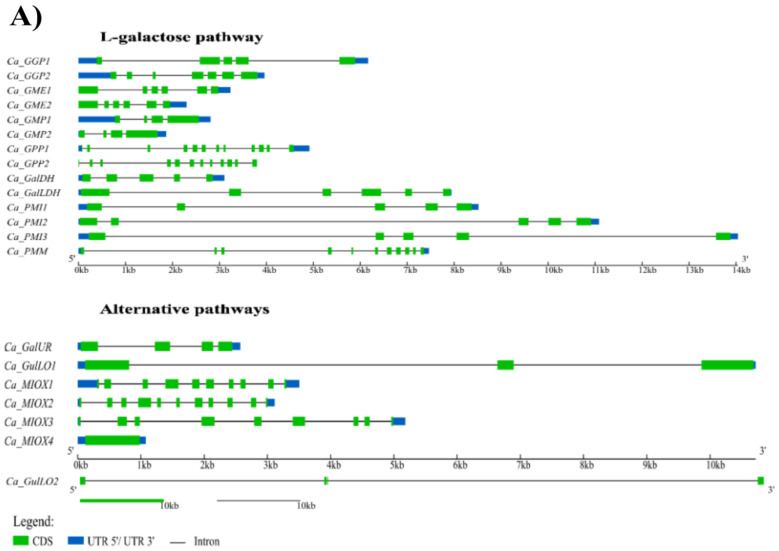

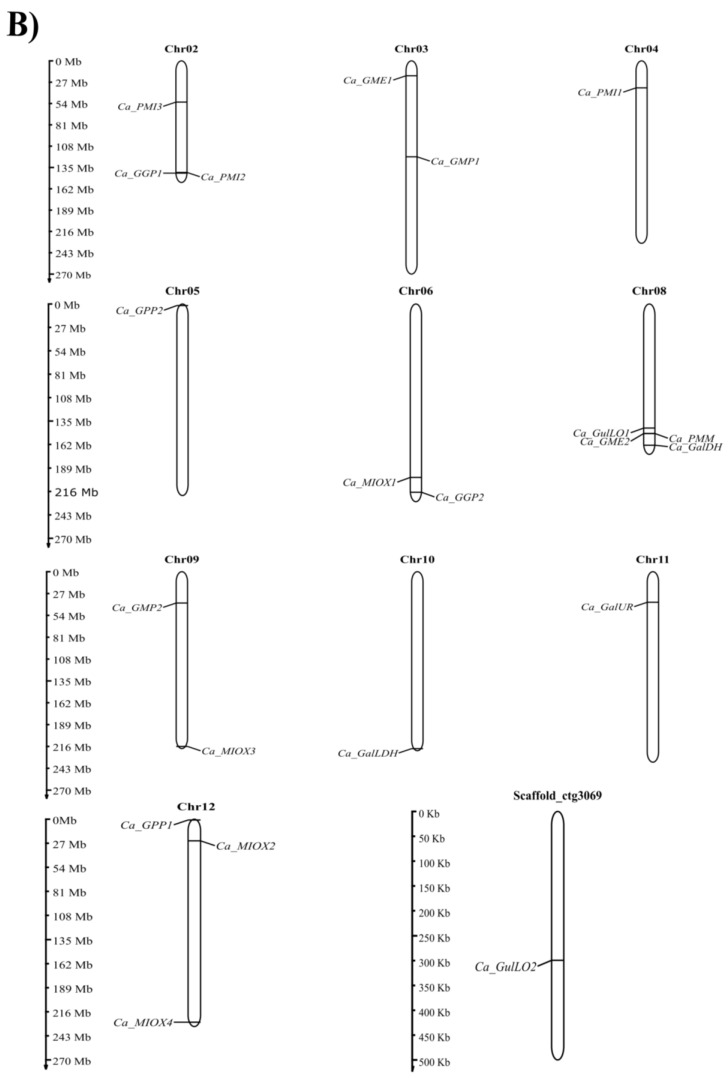
Structure and distribution of 21 ascorbate biosynthesis genes in *Capsicum annuum* L. (**A**) Disposition of exons, introns, and UTRs in each gene. (**B**) Chromosomal localization of the genes.

A total of 21 genes were identified and manually annotated as encoding enzymes in the Asc biosynthesis process of *C. annuum*. Specifically, 14 genes were associated with the D-mannose/L-galactose pathway, with the following number of genes encoding for each enzyme: one gene for PMM, GalLDH, and GalDH, two genes for GMP, GME, GGP, and GPP, and three genes for PMI. Regarding the D-galacturonate, myo-inositol, and L-gulose alternative pathways, one *GalUR*, two *GulLO*, and four *MIOX* genes, respectively, were annotated to these specific families under investigation (Figure 2A). 

In addition, these genes were found scattered in 10 out 12 chromosomes of *C. annuum* cv. UCD-10X-F1, except in Chr 1 and 7 (Figure 2B). The sequences of all deduced cDNAs and proteins from pepper are available in Table S1.

### 2.2. Expression Profile of Genes of Ascorbate Biosynthesis Enzymes during Fruit Development

**Figure 3:**
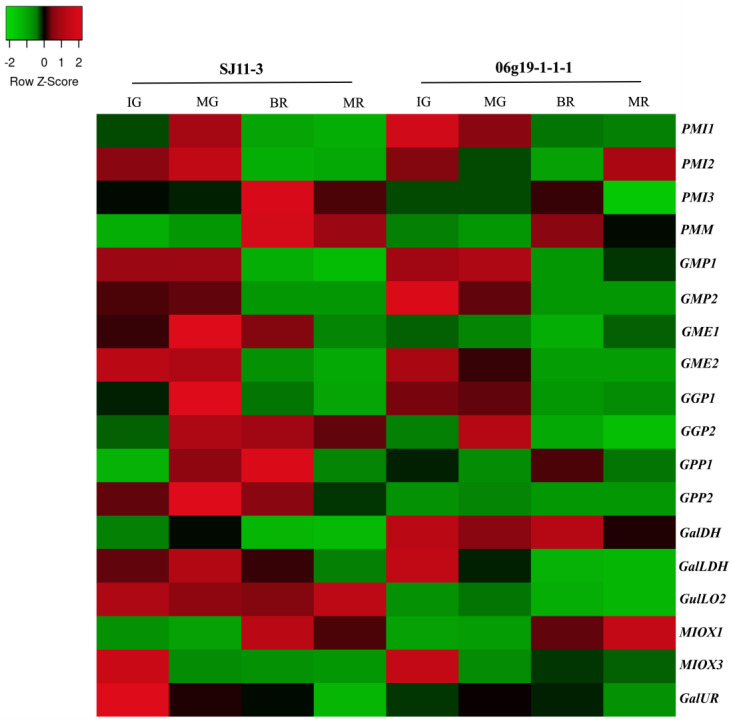
Heatmap representation of genes encoding enzymes of ascorbate biosynthesis pathways in fruit pericarps of ‘SJ11−3’ and ‘06g19−1−1−1’ pepper (*Capsicum annuum* L.) varieties at 20, 30, 40 and 50 Days After Anthesis (DAA) (PRJNA485468). Up− and down−regulated genes are indicated in red and green colors, respectively, according to Z−score values obtained from three biological replicates. Statistical significance is shown in Table S3.

**Figure 4:**
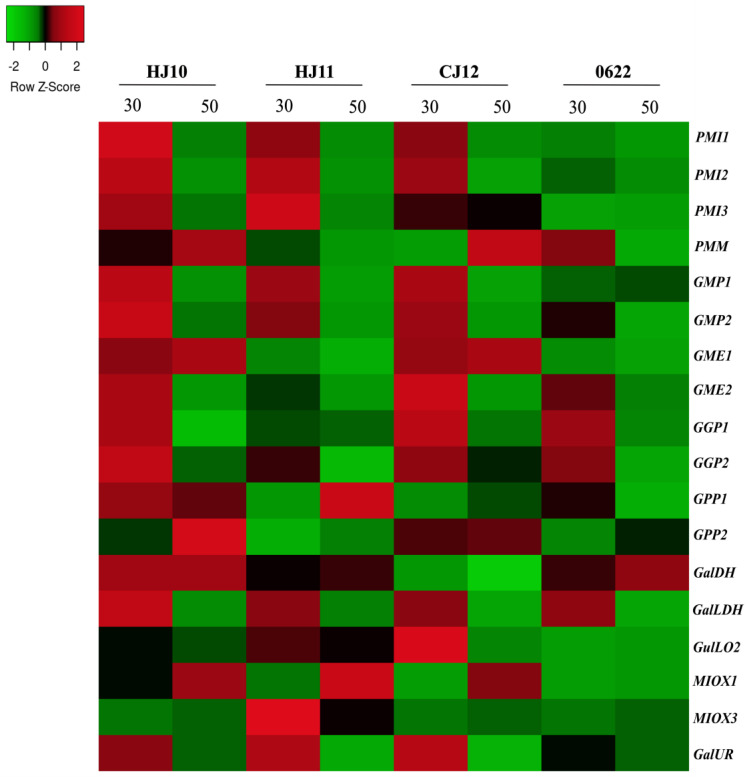
Heatmap representation of genes encoding enzymes of ascorbate biosynthesis pathways in fruit peels of four pepper (*Capsicum annuum* L.) varieties, HJ10−1, HJ11−3−1, CJ12−17−1, and 0622−1−3-2−1−3−1, at 30 and 50 DAA (PRJNA533286). Up− and down−regulated genes are indicated in red and green colors, respectively, according to Z−score values obtained from three biological replicates. Statistical significance is shown in Table S4.

**Figure 5:**
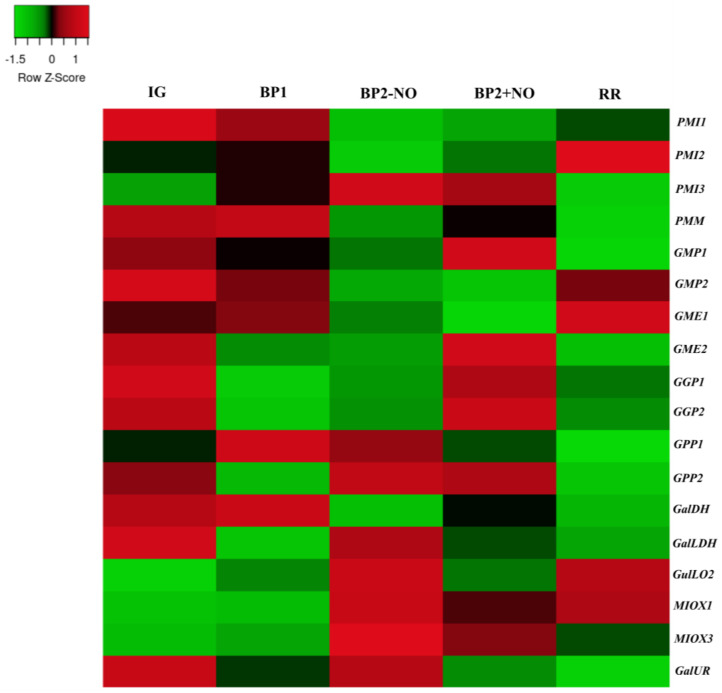
Heatmap representation of genes encoding enzymes of ascorbate biosynthesis pathways in fruit of California−type sweet pepper (*Capsicum annuum* L.) at immature green (IG), breaking point (BP), and ripe red (RR) stages, as well BP point stage treated with nitric oxide (BP2 + NO) and without NO (BP2–NO) (PRJNA668052). Up- and down-regulated genes are indicated in red and green colors, respectively, according to Z−score values obtained from 4–5 biological replicates. Statistical significance is shown in Table S5.

In the current study, three public RNA-seq bioprojects regarding pepper fruit development and ripening were selected to examine the expression pattern of genes encoding Asc biosynthesis. In general, all gene members encoding to the D-mannose/L-galactose pathway were ubiquitously expressed, and most of them exhibited high expression levels. In contrast, genes of the alternative pathways exhibited low expression levels or were even unexpressed (*GulLO1* and *MIOX2* and *4*) in these experiments (Figure 3, Figure 4 and Figure 5 and Tables S2–S5). 

In experiment 1 (PRJNA485468) [36], the transcriptome of fruit pericarps of two pepper varieties ‘SJ11−3’ (higher Asc content) and ‘06g19−1−1−1’ (lower Asc content) at the immature green (IG; 20 Days After Anthesis—DAA), mature green (MG; 30 DAA), breaker (BR; 40 DAA), and mature red (MR; 50 DAA) stages was analyzed (Figure 3 and Tables S2 and S3). Among the genes analyzed from the D-mannose/L-galactose pathway in the variety SJ11−3 (higher Asc content), all *GMP*, *GME*, *GGP*, *PMI1* and *2*, and *GPP2* members exhiited higher expression level or were up-regulated at MG stage, preceding the overproduction of Asc. While the 06g19−1−1−1 variety, mostly revealed lower levels of these transcripts and a continuous decrease from IG until MG stage. *PMI3*, *PMM*, *GGP2*, and *GPP1* genes were up-regulated until breaker stage for both varieties. However, SJ11−3 exhibited higher transcript amounts and peaked at breaker stage, when Asc increase was maximum in both varieties. In addition, transcript levels of *GMP1* and *GalDH* were higher in 06g19−1−1−1 from MG to MR. Overall, the expression of genes from alternative pathways did not seem to correlate with Asc accumulation at later stages. *GulLO2* (L-gulose pathway) expression was significantly higher in SJ11−3, mainly at IG and MR stages, while *GalUR* transcripts (D-galacturonate) showed higher amounts in IG and were absent in MR stage of SJ11−3 compared to 06g19−1−1−1. *MIOX* gene expression profile was similar in both varieties: *MIOX3* was more expressed at IG, while *MIOX1* was only expressed after the MG stage.

Regarding experiment 2 (PRJNA533286) [37], fruit peels of four varieties HJ10−1, HJ11−3−1, CJ12−17−1, and 0622−1−3−2−1−3−1, contrasting in color and shape, were analyzed at 30 (fully developed) and 50 DAA (fully mature) (Table S2). In these varieties, the expression of all *GMP*, *GGP*, *GME2*, *PMI1* and *2*, and *GalLDH* members from the D-mannose/L-galactose pathway was higher at 30 DAA (Figure 4 and Table S4). Furthermore, other members, including genes of the alternative pathways also exhibited slight up-regulation at this stage in a variety-specific manner. For instance, *PMI3* (HJ10 and 11); *PMM* (0622); *GalDH* and *GulLO2* from L-gulose pathway (CJ12); *GalUR* from D-galacturonate pathway (HJ11 and CJ12); and *MIOX3* (myo-inositol pathway) in HJ11, reinforcing the main role of D-mannose/L-galactose pathway for biosynthesis of Asc at 30 DAA. Otherwise, transcripts amount of *PMM* (CJ12 and HJ10), *GPP2* (HJ10 e HJ11), and *MIOX1* (HJ10 and HJ11) augmented at 50 DAA.

Figure 5 presents a heatmap (PRJNA668052) [35] of the genes associated with Asc biosynthesis in sweet pepper fruits, and its stages are as follows: immature green (IG), breaking point 1 (BP1), breaking point 2 without nitric oxide (BP2–NO), breaking point 2 with NO-treatment (BP2 + NO), and ripe red (RR) (Table S5). 

The large majority of genes displayed higher transcript levels at immature green stage, and their expression decreased by the ripe red stage. These included all *GMP*, *GGP*, *PMM*, *GME2*, *GPP2*, *GalDH*, and *GalLDH* of the L-galactose pathway, and *GalUR* (D-galacturonate pathway). Notably, *PMI2* and *GME1* from the principal pathway, followed by *GulLO2* and *MIOX1* and *3* from alternative pathways were up-regulated at the ripe red stage. In relation to NO treatment, *GMP1*, *GME2*, *GGP1* and *2*, *GPP2* and *GalLDH*, and *MIOX1* and *3* were more expressed at BP2 + NO compared to BP1. In addition, most of these genes from L-galactose pathway also decreased their expression at BP2-NO, while *GulLO2* increased. On the other hand, *PMI2*, *PMM*, *GMP2*, *GME1*, *GPP1*, and *GalDH* decreased their expression, most of them in both treatments, though more accentuated in BP2−NO. Overall, these results suggest that L-ascorbate biosynthesis was more active in the immature stage and in the retarding ripening treatment (BP2 + NO).

### 2.3. Expression Profile of Genes of Ascorbate Biosynthesis Enzymes under Abiotic Stresses 

**Figure 6:**
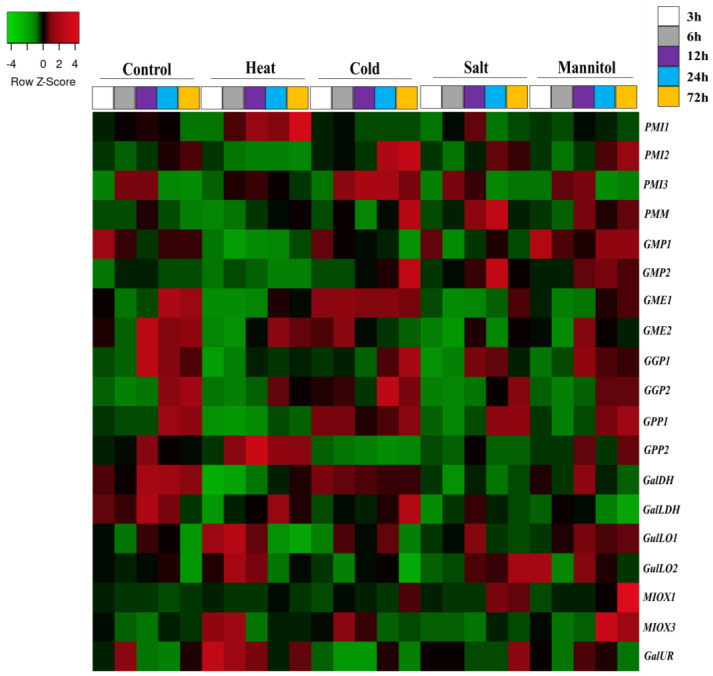
Heatmap illustration of genes encoding ascorbate biosynthesis pathways in leaves of pepper (*Capsicum annuum* L.) under heat (40 °C), cold (10 °C), salinity (NaCl−400 mM), and osmotic stress (mannitol−400 mM) (PRJNA525913). Up− and down−regulated genes are indicated in red and green colors, respectively, according to Z−score values obtained from three biological replicates. Statistical significance is shown in Table S6.

**Figure 7:**
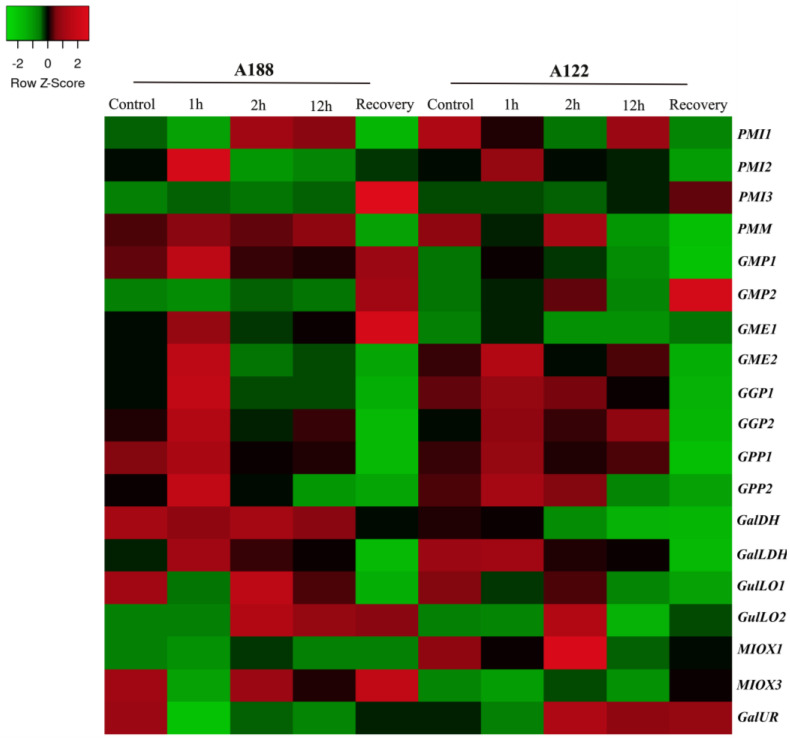
Heatmap illustration of genes encoding ascorbate biosynthesis pathways in leaves of two pepper cultivars, cold−tolerant (A188) and cold−sensitive (A122), at 0, 1, 2, and 12 h after treatment, and in rewarming after 1 h, post 72 h of cold stress (PRJNA646356). Up− and down−regulated genes are indicated in red and green colors, respectively, according to Z−score values obtained from three biological replicates. Statistical significance is shown in Table S7.

**Figure 8:**
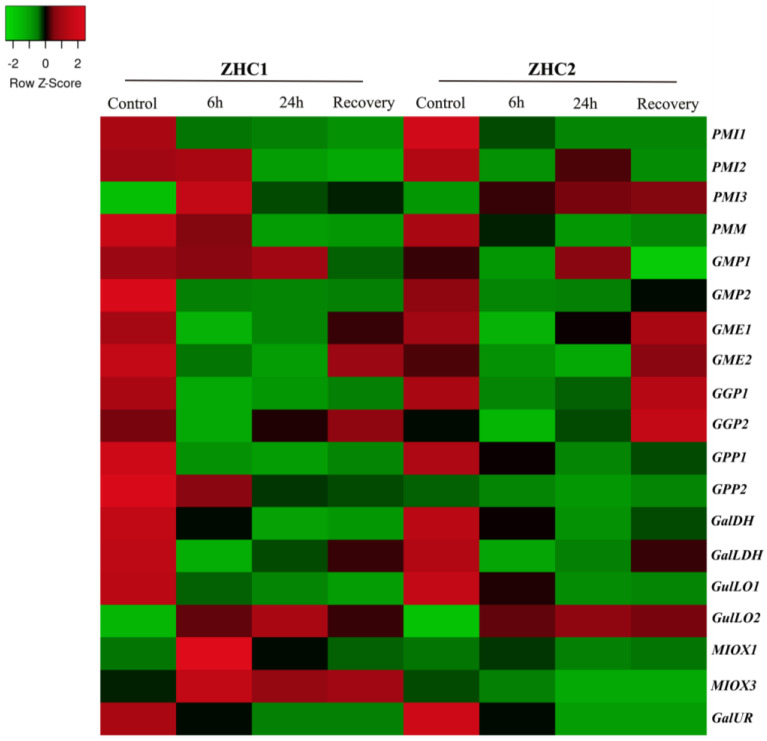
Heatmap illustration of genes encoding ascorbate biosynthesis pathways in leaves of two pepper genotypes, a susceptible (‘ZHC1’) and another tolerant (‘ZHC2’) to waterlogging at 6 h and 24 h of waterlogging stress, and 1 h post recovery (PRJNA793609). Up− and down−regulated genes are indicated in red and green colors, respectively, according to Z−score values obtained from three biological replicates. Statistical significance is shown in Table S8.

In the current study, three RNA-seq bioprojects regarding pepper leaves were accessed to examine the gene expression pattern under heat, cold, salinity, osmotic, and waterlogging abiotic stresses. In general, all gene members encoding enzymes of D-mannose/L-galactose pathway were ubiquitously expressed, and most of them exhibited high transcript levels, while genes from the alternative pathways generally exhibited lowly expression levels (Figure 6, Figure 7 and Figure 8 and Tables S6–S8).

In heat stress, plants challenged with 40 °C (PRJNA525913) [38] appeared to strongly affect the Asc biosynthesis by promoting the down-regulation of several genes, including *GMP*, *GME, GGP*, *GPP1*, *GalDH*, and *GalLDH* members from L-galactose pathway during the treatment, while *MIOX3* and *GalUR* transcript levels from alternative pathways slightly augmented until 6 h compared to the control (Figure 6 and Table S6). Moreover, the mRNA levels of *PMI1*, *GGP2*, *GPP2* (12 h), *PMI1* and *3*, and *GalLDH* (24 to 72 h) slightly increased their expression at later periods. Otherwise, the Asc biosynthesis genes in leaves under cold (10 °C) seemed to be more stimulated until 6 h. Noticeably, *GGP2* transcripts strongly increased (except in 72 h), followed by the up-regulation of *GME*, *GGP1*, and *GPP1*, with apparent contribution of *MIOX3* (myo-inositol pathway). Afterwards, most of these genes including *GMP1* and *GalDH* experienced a reduction in their expression levels. However, transcript amounts from *PMI* family and *GalLDH* showed an increase at later exposure times.

For leaves treated with NaCl 400 mM, the expression profile of genes associated with Asc biosynthesis suggested the slowdown of L-galactose pathway at all exposure times. Specifically, *GME* members and *GGP1* (except in 6 and 24 h) and *GalDH* members were sharply down-regulated at all times, followed by *GMP1*, *GGP2*, and *GalLDH* (except at 72 h), mainly at initial and later time points (Figure 6 and Table S6). Relative to alternative pathways, *MIOX3* slightly decreased their mRNA amounts at 3 h, while *MIOX1* (24 and 72 h) and *GulLO* members (72 h) increased. Regarding osmotic stress, the application of mannitol 400 mM did not affect the expression of most genes until 6 h. From 12 h onwards, the expression of *GME*, *GGP*, *GalDH*, and *GalLDH* families notably decreased, indicating an apparent reduction in Asc biosynthesis.

Figure 7 (PRJNA646356) [39] shows the gene expression of Asc biosynthesis pathways evaluated in leaves of two cultivars: a cold-tolerance (A188) and a cold-sensitive inbred line (A122) subjected to low temperature (4 °C) for 1, 2, and 12 h, and rewarming for 1 h after 72 h cold stress. Overall, tolerant plants (A188) exhibited improved responses by activation of Asc biosynthesis L-galactose pathway at the beginning and the recovery of stress. Several genes, such as *PMI2* and *3*, *GMP1*, *GME1*, *GGP1* and *2*, and *GalLDH* were up-regulated in the tolerant compared to the sensitive cultivar in these exposure times (Figure 7 and Table S7). In this line, transcript amounts of *PMI2* and *3*, *GMP1*, *GME1*, and also a member from myo-inositol pathway (*MIOX3*) were notably more expressed in the tolerant cultivar at rewarming point. In addition, *PMM*, *GMP1*, *GGP1* and *2*, *GME2*, and *GalLDH* genes decreased their expression in recovery, primarily in the sensitive plants. Genes from alternative pathways, including *GulLO* members (L-gulose), *MIOX1,* and *GalUR* (D-galacturonate pathway) were induced mainly in the sensitive cultivar after 1 h of exposure.

The expression pattern of genes involved in ascorbate biosynthesis in leaves of two hot peppers genotypes: waterlogging-sensitive (‘ZHC1’) and waterlogging-tolerant (‘ZHC2’) at 6 and 24 h of waterlogging stress, and 1 h after recovery is shown in Figure 8 and Table S8 (PRJNA793609) [40]. In general, both varieties triggered similar expression profiles for the large majority of genes with specific peculiarities. Both seemed to immediately reduce Asc biosynthesis in response to waterlogging stress, while its activation was observed in the recovery treatment mainly in tolerant plants with apparent contribution of L-gulose pathways. Among them, *PMI1*, *PMM*, all *GME*, *GGP*, *GPP*, *GalDH*, *GalLDH*, and *GalUR* were down-regulated during waterlogging exposure in both, mainly in tolerant plants. Both genotypes indicated an increase in Asc biosynthesis by the overexpression of *GGP2* genes, assisted by *GalLDH* under recovery, mostly observed in the tolerant genotype (ZHC2). Interestingly, *GulLO2* transcripts strongly augmented in both genotypes during the treatment, suggesting some contribution in response to waterlogging.

### 2.4. Expression Profile of Genes of Ascorbate Biosynthesis under Biotic Stresses

**Figure 9:**
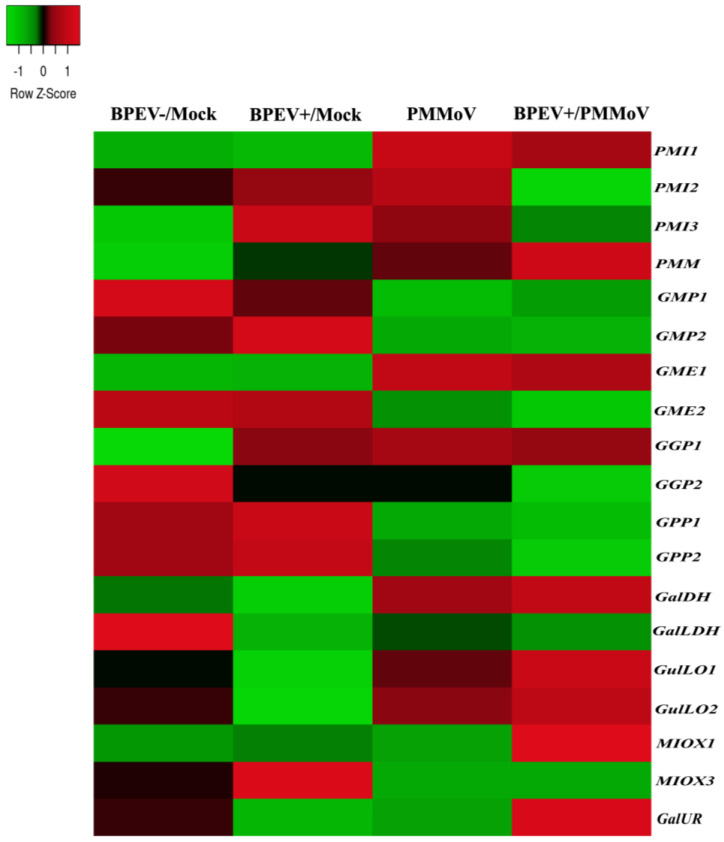
Heatmap illustration of genes encoding enzymes of ascorbate biosynthesis pathways in leaves of two bell pepper near-isogenic lines (NIL), one infected with bell pepper endornavirus (BPEV+) and other with BPEV-free, and both further inoculated with PMMoV as follows: BPEV−/Mock, BPEV+/Mock, BPEV−/PMMoV, and BPEV+/PMMoV (PRJNA588750). Up− and down−regulated genes are indicated in red and green colors, respectively, according to Z−score values obtained from two biological replicates. Statistical significance is shown in Table S9.

To obtain an overview of the expression profile of genes involved in pepper Asc biosynthesis that succumbed to pathogenic infections, data from two transcriptomic experiments were analyzed. Investigation into leaf transcriptome of two bell pepper near-isogenic lines (NIL), one infected with bell pepper endornavirus (BPEV+) and other BPEV-free, was conducted, and both were further inoculated with pepper mild mottle virus (PMMoV) resulting in the following treatments: BPEV−/Mock, BPEV+/Mock, BPEV−/PMMoV, and BPEV+/PMMoV (PRJNA588750; Table S2) [41]. The studied genes from the L-galactose pathway were apparently more responsive to PMMoV and BPEV+/PMMoV infections with some interesting peculiarities (Figure 9 and Table S9). In all treatments, *PMM* and *GGP1* mRNA increased, while *GMP1*, *GGP2*, and *GalLDH* decreased their levels, mainly in leaves infected with BPEV+/PMMoV. However, *GalDH* and remarkably *GME1* were up-regulated, whilst *GME2* and *GPP1* and *2* transcripts reduced only in PMMoV and BPEV+/PMMoV, mostly in the last one. Interestingly, genes from the L-gulose pathway *GulLO1* and *2* and *GalUR* (D-galacturonate) were up-regulated in BPEV+/PMMoV compared to BPEV+, indicating some contribution to Asc biosynthesis process, while *MIOX3* transcripts were augmented only in BPEV+.

**Figure 10:**
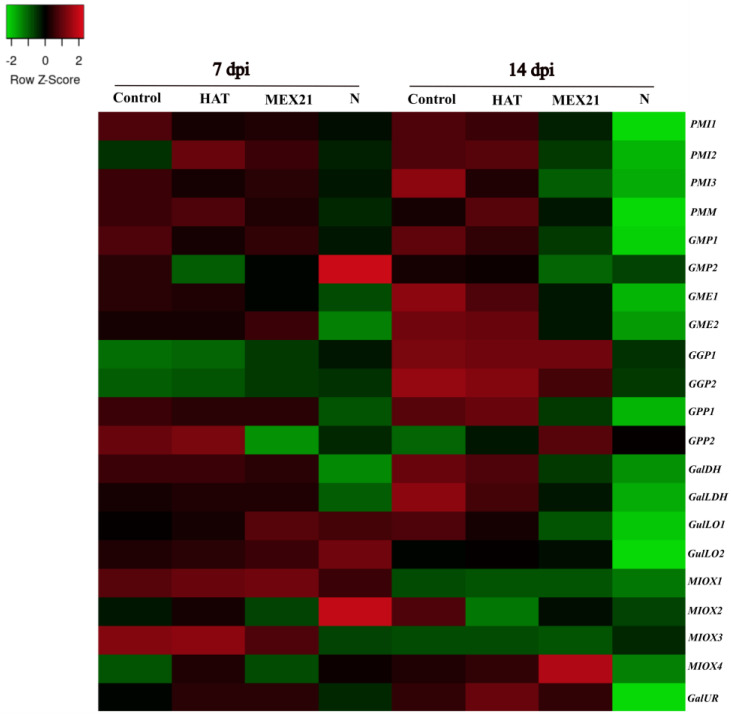
Heatmap illustration of genes encoding enzymes of ascorbate biosynthesis pathways in apical steams infected with three tobacco etch virus (TEV) strains (HAT, Mex21, and N) at 7 and 14 days post-inoculation (dpi) (PRJNA476480). Up− and down−regulated genes are indicated in red and green colors, respectively, according to Z−score values obtained from two biological replicates. Statistical significance is shown in Table S10.

Subsequently, genes of ascorbate biosynthesis pathways revealed specific strain responses to apical stems infected with three tobacco etch virus (TEV) strains (HAT, Mex21, and N) at 7 and 14 days post-inoculation (dpi) (PRJNA476480) [42]. The infection caused by severe N strain, negatively affected the expression of genes from L-galactose, myo-inositol, and L-gulose pathways, mainly at 14 dpi (Figure 10 and Table S10). Among them, *GMP1*, *GME1* and *2*, *GPP1*, *GalDH*, and *GalLDH*, and notably *MIOX3* (myo-inositol pathway) experienced a decrease in their mRNA amounts at 7 dpi. Remarkably, almost all genes from L-galactose, *MIOX1* and *3*, *GulLO1*, and *GalUR* from alternative pathways were down-regulated at 14 dpi in the N strain, whereas HAT and Mex21 did not alter gene expression at 7 dpi, while transcript amounts of *PMI3*, *GMP1*, *GME1* and *2*, *GGP2*, *GPP1*, *GalDH*, *GalLDH*, and *GulLO1* were sharply reduced by Mex21 inoculation at 14 dpi.

### 2.5. Expression Profile of Genes of Ascorbate Biosynthesis Enzymes under Phytohormones Treatments

**Figure 11:**
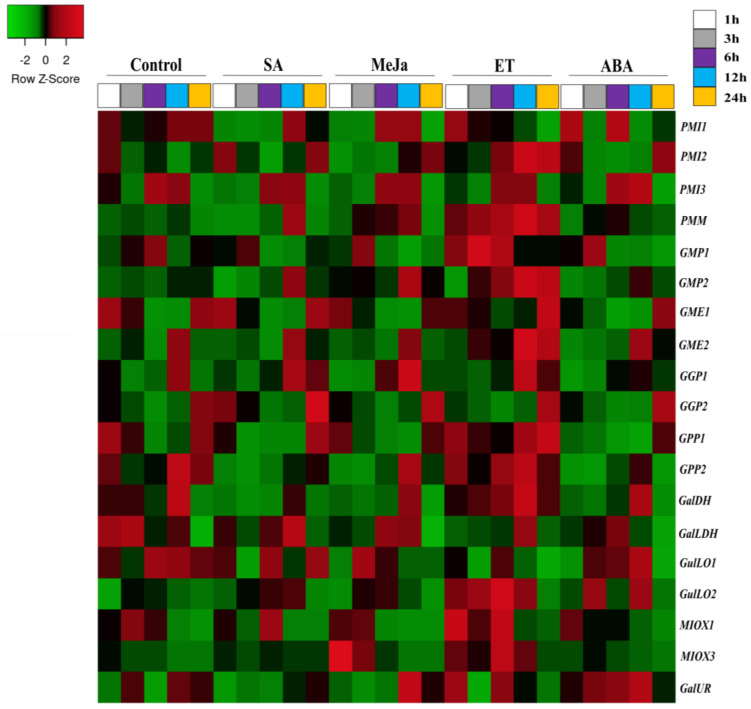
Heatmap representation of genes encoding ascorbate biosynthesis pathways in pepper leaves under treatment with exogenous sodium salicylate 5 mM (SA), methyl jasmonate 100 μM (MeJa), ethephone 5 mM (ET), and abscisic acid 100 μM (ABA) phytohormones at 1, 3, 6, 12, and 24 h (PRJNA634831). Up− and down−regulated genes are indicated in red and green colors, respectively, according to Z−score values obtained from three biological replicates. Statistical significance is shown in Table S11.

The gene expression related to Asc biosynthesis pathways in pepper was analyzed through leaf application of sodium salicylate (SA), methyl jasmonate (MeJa), ethephone (ET), and abscisic acid (ABA) phytohormones at 1, 3, 6, 12, and 24 h (PRJNA634831) [43]. Generally, phytohormones promoted different expression patterns, while MeJa and ABA down-regulated the expression of the large majority of genes involved in Asc biosynthesis pathway, and salicylate and mainly ethylene seemed to increase activity of principal and alternative pathways at all points studied (Figure 11 and Table S11).

Regarding salicylate (SA), *GGP2*, the gene encoding the rate limiting step of D-mannose/L-galactose pathway was substantially up-regulated at all treatments, except at 12 h. In addition, transcript amounts of *PMM*, *GGP1,* and *GalLDH*, followed by *MIOX1* and *3* (myo-inositol pathway) were augmented at the last two exposure times. However, the mRNA levels of *GPP* and *GalDH* (at 1, 3, and 12 h), *PMI* and *GMP1* (at 1 or 6 h), and *GalLDH* (at 3 h) slightly decreased in relation to controls. Ethephone (ET), a chemical precursor of ethylene, seems to activate Asc biosynthesis in leaves at all times of studied exposure, except at 1 h, when *GME1*, *GGP2*, and *GalLDH* transcripts levels reduced (Figure 11 and Table S11). Noticeably, from 6 h onward, the large majority of genes from L-galactose pathway (*PMI2*, *PMM*, *GMP*, *GME*, *GGP*, and *GPP*) in almost all exposure times, as well as *GalDH* and *GalLDH*, both at 24 h, increased their expression (Figure 11 and Table S11). *GME2* member was particularly up-regulated, evidencing its responsiveness to ET. In agreement with this, specific members from alternative pathways (*GulLO2* and *MIOX1* and *3*) were sharply stimulated at 1 until 12 h, evidencing some contribution.

In the case of methyl jasmonate (MeJa), several genes from L-galactose pathway, including *PMI*, *GME1*, *GGP1*, *GPP2*, *GalDH*, and *GalLDH*, were down-regulated after 1 and 3 h of treatment. Interestingly, *MIOX3* (myo-inositol pathway) was strongly augmented at the same time, while *GulLO1* exhibited an irregular pattern (down- and up-regulation, respectively) (Figure 11 and Table S11). Subsequently, MeJa appears to increase mRNA amounts of *GalLDH* (6 h), and mainly of *GMP* and *GGP* family in the two later treatments. In leaves, abscisic acid (ABA) provoked the decrease in expression of several genes from L-galactose pathway at 1, 3, and 12 h including, *GME*, *GGP*, *GPP*, *GalDH,* and *GalLDH* genes. Nevertheless, it appears that Asc biosynthesis was stimulated at 6 and 24 h by the up-regulation of *PMI*, *PMM*, and mostly *GME2* and *GGP2* members, despite some down-regulated genes in these exposure times.

### 2.6. Cis-Acting Elements in the Promoter Region of Genes Involved in Ascorbate Biosynthesis

**Figure 12:**
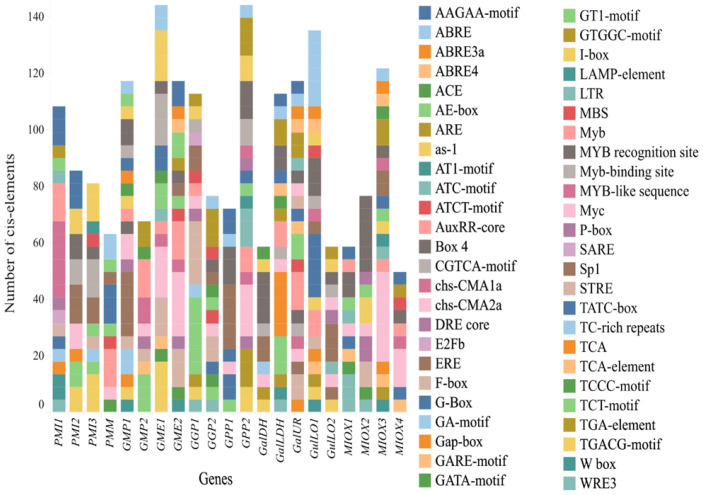
Illustration of 50 most abundant cis-regulatory elements identified in the promotor region (1000 bp) of genes encoding enzymes of ascorbate biosynthesis pathways in pepper (*Capsicum annuum* L.).

Cis-regulatory elements analysis at 1000 bp upstream of the translation starting codon (ATG) predicted putative elements for all 21 genes encoding Asc biosynthesis (Table S12). Among these, the most abundant 50 cis-elements, summarized in Figure 12, were associated with light, phytohormones, abiotic stresses, and anaerobic induction. Genes from the principal (*GMP1* and *GPP1*) and alternative pathways (*GalUR*, *MIOX3*, and *GulLO1*) exhibited the highest number of elements.

A total of 21 cis-acting elements (TCT-motif, ATCT-motif, GATA-motif, TCCC-motif, GT1-motif, G-Box, GATA-motif, GT1-motif, Box-4, AE-box, I-box, Sp1, Gap-box, LAMP-element, chs-CMA1a and 2a, ACE, GA-motif, GTGGC-motif, and AT1-motif) identified in all genes were related to light responsiveness (Figure 12 and Table S12). Regarding phytohormone-responsive elements, CGTCA-motif, and TGACG-motif associated with MeJa; P-box, TATC-box, and GARE-motif (gibberellin); ABRE (abscisic acid); TGA-element (auxin); and TCA-element related to salicylic acid were found in most of L-galactose and alternative pathway families that were studied (Figure 12). Yet, drought-responsive MBS element was found in *GGP1* and *2*, *GME2*, *PMM*, and *GalUR* promoters; LTR, TCA-motif, or TC-rich repeats of low temperature cis-acting elements were verified in all *GPP*, *PMI*, *GulLO2*, and *MIOX1* members. Furthermore, ARE, involved in the anaerobic induction, was abundantly detected in several genes, including *GME*, *GGP,* and *GPP* families. Curiously, different cis-regulatory elements belonging to the MYB, DRE-core, and MYC families were abundantly found in the promoter region of several genes, mainly in *GGP1* and *2*, *GME2*, and *GalLDH*.

## 3. Discussion

*Capsicum annuum* L. is widely recognized as a primary source of vitamin C and spice compounds required for human nutrition [35]. In peppers, ascorbate (Asc) is higher in fruit and accumulates as the ripening proceeds until over-ripe stages [33,36], similar to tomato and strawberry fruits [23,44]. The ascorbate accumulation during the maturation of tomato and pepper indicates that the protective function of the antioxidative system plays a fundamental role in the ripening process, given that fruits experience high respiratory rate and ROS production [44,45,46]. Nevertheless, the molecular bases responsible for Asc pools regulation in pepper is still unknown despite its global importance and growing number of genomes and transcriptomes sequences available in public databases nowadays. To gain knowledge on molecular mechanisms regarding the significance of Asc biosynthesis pathways and also to detect promising regulatory genes during fruit development and under environmental stresses simulations, nine different transcriptomic datasets were explored.

In the present study, we identified 21 genes encoding enzymes of the four proposed ascorbate biosynthesis pathways in pepper. Analyses revealed that these genes are unevenly distributed in 10 out of 12 chromosomes, exposing the structural complexity intrinsic to the biosynthesis of this multifaceted metabolite. Moreover, the transcriptional profile of genes revealed the central contribution of the L-galactose pathway for Asc contents determination in pepper fruit development and ripening, since the expression pattern of genes from alternative pathways did not demonstrate significant association (Figure 3, Figure 4 and Figure 5 and Tables S3–S5). Previously, the quantification of ascorbate contents for two varieties ‘SJ11–3’ (higher Asc content) and ‘06g19–1–1–1’ (lower Asc content) showed that it accumulated at breaker, and peaked at ripe stage [36]. Here, we found that all *GMP*, *GME*, *GGP*, *PMI1* and *2*, and *GPP2* maintained high expression levels, and were up-regulated at mature green stage (MG) in ‘SJ11-3’, evidencing their primary role in Asc accumulation. In addition, the up-regulation of *PMI3*, *PMM*, *GGP2,* and *GPP1* members until breaker stage mainly in SJ11-3 variety seemed to play a key role in Asc pools maintenance (Figure 3 and Table S3). In both of the following experiments, once, all *GMP*, *GGP*, *GME2*, *PMI1* and *2*, *PMM*, *GPP2*, and *GalLDH* genes were up-regulated at immature or mature green stage in the other five varieties analyzed (Figure 4 and Figure 5 and Tables S4 and S5). Thus, specific gene member induction at a specific time may reflect the diversity in regulatory mechanisms displayed by each cultivar for fine-tuning of Asc levels. In fact, previous reports also verified higher expression for most of these families at these stages and highlighted the pivotal role of the L-galactose pathway [33,34], supporting these results.

One of the strategies to delay ripening and extend the postharvest time of fruits is the application of nitric oxide (NO), since this molecule inhibits ethylene synthesis and respiration [47]. NO-treated pepper fruit stimulated the L-galactose pathway by up-regulating *GMP1*, *GME2*, *GGP1* and *2*, *GPP2*, and *GalLDH* members compared to control (BP1) (Figure 5 and Table S5), indicating that NO treatment improved the antioxidant status by inducing an overproduction of ascorbate. Indeed, NO-treated pepper fruit not only increased the Asc content by 40%, but also glutathione levels and the activities of GalLDH and ascorbate peroxidase [35,48]. Therefore, NO treatment is a promising alternative for improving the postharvest quality by biofortifying pepper fruit with ascorbate. Overall, based on expression level and up-regulation intensity before Asc accumulation, *GGP2*, *GME2*, *GMP1*, *GPP1*, and *GalLDH* may be highlighted as major points and members for the regulation of Asc levels in pepper fruits. In several species, the L-galactose pathway was the primary pathway, and high transcript levels from these families also preceded or coincided with Asc accumulation in fruits of acerola [25,27], jujube [22], *GPP* in tomato [44], and *GGP3* and *GME* in kiwifruit [13,49], supporting our findings.

Here, stresses simulations affected the gene expression pattern of the principal and secondary pathways in a type- and temporal-specific manner. Climate changes represent a challenge to plant growth and survival, especially under extreme weather events. Ascorbate improves plant resilience to various environmental stimuli by maintaining normal ROS levels [50,51]. The optimal temperature for pepper normal development ranges from 21 to 28 °C [39]. In this study, heat and cold treatments provoked an opposite effect in the principal Asc biosynthesizing pathway. While high temperature down-regulated the expression of several genes, low temperature increased their mRNA levels, mainly at the beginning and rewarming times (Figure 6 and Figure 7 and Tables S6 and S7). These findings indicate that high temperature severely reduces, but cold increases ascorbate content of pepper leaves, as previously reported in tomato fruit [44], tea [52], and kiwi leaves [53]. Data also suggest that in cold-tolerant cultivar (4 °C), L-galactose and myo-inositol pathways activation in the beginning and recovery points indicate that rapid and efficient Asc production may be in the first line for cold response (Figure 7 and Table S7). In this same cultivar, several genes encoding photosystem I and II, cytochrome b6/f complex, and F-ATPase were significantly up-regulated in the tolerant plants at 1 and 2 h of stress simulation [39], pointing that Asc biosynthesis augment was supplied by high photosynthesis flux to overcome cold negative stimuli. For corroboration, the up-regulation of *GPP* [44], *GME,* and *GalLDH* members [54,55] from the principal pathway in tomato fruits and *GME* in kiwifruit [53] were also linked to Asc and tolerance gains under cold conditions. The slight up-regulation of specific *MIOX* and *GulLO* members mainly at the beginning (Figure 6 and Figure 7 and Tables S6 and S7) indicate that both stresses may induce alternative pathways in the attempt to maintain or increase Asc pools, though apparently insufficient under heat.

Regarding saline and mannitol treatments (400 mM), mRNA amount of the L-galactose pathway reduced along the treatment, while those of the L-gulose and myo-inositol pathways slightly increased at final exposure times (Figure 6 and Table S6). These data show that the induction of alternative pathways is possibly an attempt to balance the slowdown suffered by the principal pathway. In strawberry fruit, drought and salt stresses strongly decreased the ascorbate contents, which were correlated with the down-regulation of *GalLDH* [56]. Moreover, low and moderate salt exposures reduced ascorbate levels and *GalLDH* expression levels in leaves and mainly in pepper fruits, from 7 to 21 days of stress, as a result of Na^+^ accumulation [57]. For corroboration, high concentration of mannitol increased the oxidized and decreased the reduced form of Asc, while at lower (100 mM) concentration, the Asc content was augmented [58]. Moreover, waterlogging treatment indicated a reduction in the Asc biosynthesis, since various genes from L-galactose pathway were down-regulated in tolerant and sensitive plants, but stimulated at the recovery point mostly in the waterlogging-tolerant cultivar (Figure 8 and Table S8). Waterlogging promotes a hypoxic environment that triggers ROS overproduction [59], verified in the sensitive cultivar. With regard to these cultivars [40], it was found that waterlogging-sensitive plants experienced severe oxidative damage, while the tolerant one managed well with ROS production and avoided oxidative stress by increasing carotenoids and amino acids contents, which may balance the apparent decrease in Asc biosynthetic process. Sensitive and tolerant apple plants treated with ascorbate increased their resistance to waterlogging by improving their antioxidant activity and increasing their Asc recycling enzymes [59]. In this sense, L6138 wild tomato line’s tolerance to waterlogging and its combination with heat was associated with higher protein synthesis and endogenous Asc production compared to sensitive genotypes [60].

Collectively, these findings show severe unbalancing of ascorbate biosynthesis process under stress; its apparent decrease under heat, salt, and osmotic conditions seems to be a result of the slowdown of the L-galactose pathway and the excessive use of Asc for protecting plants from cellular injury, as proposed by Li and collaborators [61]. Since ascorbate biosynthesis is down-regulated by such stresses, which virtually may result in reduced endogenous Asc content, its exogenous application has been successfully employed to prevent oxidative stress and confer plant tolerance by restoring it and triggering crucial priming of adaptive responses [62,63]. In this sense, exogenous ascorbate improved cell turgidity, chlorophyll levels, and growth of strawberry plants subjected to heat [64]. Tomato seedlings exposed to severe saline stress showed improved plant recovery and 50% survival [65], while sweet pepper seedlings showed enhanced antioxidant status, growth, and yield under moderate-to-high NaCl concentration and drought stresses [66,67,68] after ascorbate application. Therefore, genotypes that maintain or improves antioxidant status may manage better the negative stress effects, as verified in this study in cold and waterlogging tolerant cultivars.

Depending on the type of pathogens and strains, genes of the L-galactose and alternative pathways displayed particular up- or down-regulation behavior (Figure 9 and Figure 10 and Tables S9 and S10). In fact, the poor responsiveness of genes to BPEV infection may be related to its weak pathogenicity [41]. However, infection with PMMoV, an acute virus, and the mixed infection (BPEV+/PMMoV) down-regulated various genes of the L-galactose pathway, despite the remarkable up-regulation of *GME1* followed by *PMM* and *GalDH* (Figure 9 and Table S9). Furthermore, the inoculation with Mex21 and N strains, which cause moderate and severe disease, respectively, down-regulated various genes of the L-galactose pathway mostly at 14 dpi. Notably, both viruses substantially induced *GulLO* and *MIOX3* members from secondary pathways (Figure 9 and Figure 10 and Tables S9 and S10). Studies related to non-enzymatic antioxidant response to virus–plant interaction are still scarce, and there is no record for peppers. Here, we speculate that PMMoV, Mex21, and N strains inhibited Asc biosynthesis process through the primary pathway, while L-gulose and myo-inositol pathways seem to be induced in an attempt to compensate it. In accordance, *Eggplant Mottled Dwarf Virus* (EMDV) substantially decreased total Asc in the leaves of two ecotypes at 7 and 21 dpi by suppression of genes of the L-galactose and D-galacturonate pathways [69]. Wounding treatment, as a simulation of biotic stress response, reduced ascorbate content in kiwifruit species, despite the up-regulation of *GPP* and *GME* genes [53,61]. In acerola leaves, the up-regulation of L-galactose genes after wounding was interpreted as a molecular response for rescuing Asc depletion [27].

Ascorbate–hormone crosstalk is recognized to be fundamental for plant plasticity and adaptive modulation under normal and stressful environmental conditions, given that Asc is a cofactor necessary for enzymes involved in the biosynthesis of gibberellins, ethylene, and abscisic acid [51,70,71,72]. In this work, MeJa and ABA down-regulated the large majority of genes of the L-galactose pathways, mostly at the beginning of application, while SA and mainly ET induced them in almost all time points (Figure 11 and Table S11). With regard to genes encoding enzymes of alternative pathways, *GulLO* and *MIOX* members were time-specific slightly or strongly expressed by SA, MeJa, and ET. Therefore, results suggest that SA and ET signaled an increase in ascorbate levels by stimulation of L-galactose, complemented by myo-inositol and L-gulose pathways, and its decrease is triggered by MeJa and mainly by ABA. According to [73], ethylene and ABA act antagonistically in the regulation of ascorbate biosynthesis. While ethylene or its precursor ACC increased Asc levels, ABA reduced it. As a result, ethylene prevented but ABA promoted the accumulation of ROS. In addition, ascorbate-deficient vtc-1 *Arabidopsis* mutant accumulated ABA and the oxidized form, dehydroascorbate [74]. Moreover, ABA reduced the *AceGGP3* expression and Asc amounts in kiwifruit by repressing the expression of *AceMYBS1* [49]. In this sense, ABA application reduced ascorbic acid and glutathione levels, but increased the expression levels of antioxidant enzymes in *C. annuum* [75]. Moreover, exogenous ethylene and gibberellin augmented Asc content in citrus [76], while *GPP* mRNA levels increased in tomato fruits treated with ethylene [44]. In accordance, SA treatment increased endogenous Asc levels and expression of L-galactose pathway genes and reduced chilling injury of kiwi and pomegranate fruits [53,61,77]. In contrast, Asc levels were increased by ABA application in kiwi [53,61] and MeJa in carambola fruits [78], despite *GPP* and *GME* expression remaining unaffected by ABA.

Regarding the contribution of pathways, genes of the L-galactose pathway revealed ubiquitous, high expressing levels and up-/down-regulation pattern that highlight its constitutive and central role in Asc-biosynthesizing process in all pepper organs, and stress and phytohormone stimuli are evaluated here (Figure 6, Figure 7, Figure 8, Figure 9, Figure 10 and Figure 11; Tables S6–S11), as verified in tomato [44,55], *Arabidopsis* [73,74], acerola [25,27], and tea [52] species. However, expression profile supports evidence that alternative pathways could play a significantly secondary role, and even an insignificant one, given its genes’ low- and even non-expression patterns (cpm normally < 3). Under normal conditions (including fruit development), the effects of heat, salt, osmotic, and ABA treatments on alternative pathways appear to be insignificant, despite slight fluctuation in gene expression (Figure 3, Figure 4, Figure 5, Figure 6 and Figure 11 and Tables S3–S6 and S11), whereas under cold, waterlogging, pathogenic infection, MeJa, SA and ethephone, and induction of genes from myo-inositol and/or L-gulose pathways indicate some complementary role in attenuation of, maintenance of, or increase in Asc biosynthesizing activity in attempt to balance L-galactose fluctuations (Figure 6, Figure 7, Figure 8, Figure 9, Figure 10 and Figure 11 and and Tables S6–S11). Nevertheless, further wet lab experimentations are required to clarify the relevance of these pathways.

Regarding potentially key regulatory genes, these findings indicate the major role of *GGP2*, and the complementary role of *GME1*, *2*, and *GalLDH* members from the principal Asc biosynthesis pathways for fine-tuning adjustments in the biosynthetic process in specific stress/phytohormone types and exposure periods. For instance, cold stress remarkably increased *GGP2* transcripts amounts, followed by the up-regulation of *GME*, and *GPP1* (Figure 6 and Figure 7 and Tables S6 and S7). Under waterlogging recovery and SA application, *GGP2* overexpression was assisted by *GalLDH* (Figure 8 and Figure 11 and Tables S8 and S11); while under longer periods of heat, NaCl, osmotic, and biotic stresses, *GGP2* was sharply down-regulated, followed by *GME*, *GalDH,* and *GalLDH*. Interestingly, *GME1* and *GME2* were the most responsive to pathogen and ET (Figure 9 and Figure 11 and Tables S9 and S11). Among genes from alternative pathways, *MIOX3* and *GulLO2* were notably responsive to cold, waterlogging, ET, SA, and pathogenic infections (Figure 6, Figure 7, Figure 8, Figure 9 and Figure 10 and Tables S6–S10). Various studies using these genes to manipulate Asc metabolism successfully increased ascorbate content and plant tolerance to environmental stresses, thus clarifying their key regulatory role. *GGP3* member is the principal regulatory gene in *Actinidia* species; its overexpression augmented Asc content by 6 to 22,7-fold [13,49]. Rice plants overexpressing *GGP* showed a 2.5-fold increase in ascorbate and salt tolerance [28]. Also, *Arabidopsis* and rice leaves overexpressing *GME* increased Asc levels by ~1.5-fold and improved drought and salt resilience [28,29]. The manipulation of *GalLDH* in tobacco transformed plants and enhanced Asc, growth, and shoot length under salt imposition [30], whereas *GPP* was the only overexpressed and strongly correlated with tomato response to stresses and hormone exposures [44]. In addition, the overexpression of *GulLO* and *MIOX* from yeast or rat in *Stylosanthes guianensis* and *Arabidopsis* augmented ascorbate by 1.5 to 3.1-fold enhancing chilling, salt, and heat tolerance [31,79]. Nevertheless, the overexpression of only one gene has generally revealed to be insufficient to reach elevated ascorbate amounts of transformed plants [6,61]. Based on this knowledge, we recommended as a strategy, the co-overexpression of multiple steps by the combination of *GGP2* members with one or two more indicated members to satisfactorily increase the pathway flux, thus achieving higher ascorbate contents in engineered crops.

To corroborate evidence for responsiveness of genes, putative cis-acting elements were investigated. Here, at least one light-responsiveness element was found in every Asc biosynthesis genes (Figure 12 and Table S12), confirming that their expression regulation is dependent on light activation [27,44,80]. Moreover, cis-acting drought-responsive MBS, MYB and MYC, and specific elements hormone-responsive were all found in the promoter region of *GGP1* and *2* and *GME2,* which emphasize their putative regulatory role in such conditions (Figure 12 and Table S12). MBS is a MYB-binding site induced by drought, also detected in *GME* from *Actinidia* spp. [61] and various genes of the L-galactose pathway in tomato [44]. Recently, the expression profile of *MYB16* and *GalLDH* were correlated to higher Asc amounts in chilli pepper fruit [46]. Noticeably, *AceMYBS1* activates the expression of *AceGGP3*, the main gene responsible for Asc accumulation in kiwifruit [49]. In addition, Dehydration-Responsive Elements (DRE-core) found in the promoter region of *GPP2* and *GulLO2* genes, targets of DREB TFs, were associated with rice tolerance to heat, salt, cold, and drought [81]. Here, LTR, TCA-motif, and TC-rich repeats low temperature elements detected in responsive genes (*GPP2*, *PMI2* and *3,* and *MIOX3*) evidence their role in pepper plants’ resilience to chilling. Interestingly, the anaerobic-induction ARE, abundantly detected in the promotor region of *GGP, GME,* and *GPP* also found in *GPP* from kiwifruit and tomato [44,53,61], highlighting the major role of these members in Asc biosynthesis modulation under hypoxic conditions provoked by waterlogging.

## 4. Materials and Methods

### 4.1. Identification and Annotation of Genes Involved in Ascorbate Biosynthesis

Gene sequences encoding enzymes of ascorbate biosynthesis were retrieved from *Capsicum annuum* L. genome, cultivar UCD-10X-F1, deposited in the RefSeq Representative genome database from GenBank-NCBI (http://www.ncbi.nlm.nih.gov). Genome searches were carried out with the tBLASTn tool considering the e-value < 10^−5^ [82] using query proteins from *Arabidopsis thaliana* retrieved from the non-redundant protein database. The following gene families associated with Asc biosynthesis process were retrieved: D-mannose/L-galactose pathway, phosphomannose isomerase (*PMI*), phosphomannose mutase (*PMM*), GDP-mannose pyrophosphorylase (*GMP*), GDP-mannose-3’,5’-epimerase (*GME*), GDP-L-galactose transferase (*GGP*), L-galactose-1-phosphate phosphatase (*GPP*), L-galactose dehydrogenase (*GalDH*), L-galactone-1,4-lactone dehydrogenase (*GalLDH*); L-gulose pathway (L-gulono-1,4-lactone oxidase—*GulLO*); myo-inositol pathway (myo-inositol oxygenase—*MIOX*); and D-galacturonate pathway (D-galacturonate reductase—*GalUR*).

Gene annotation was performed manually following the strategy proposed by [83]. UTR, exon, intron, and ORF regions were determined with BLASTn searches in reference mRNA sequences deposited in (refseq_rna) and transcriptome shotgun assembly (TSA) transcripts databases from GenBank. The deduced cDNA sequences were translated into amino acid sequences using the ExPASY Translate tool (http://web.expasy.org/translate). Furthermore, the validation of deduced protein sequences was performed by comparing them with homologous sequences deposited in non-redundant protein sequences (nr) databases using the BLASTp tool. MG2C_v2.1 online tool [84] was used to visualize the distribution of genes in chromosomes, while gene structure was presented using Gene Structure Display Server—GSDS 2.0 online tool [85].

### 4.2. Gene Expression Analyses Using Capsicum Annuum RNA-Seq Experiments

Nine deep RNA sequencing bioprojects containing tens of millions of short reads (50–300 bp) of biological replicates were accessed to provide the expression profile of genes encoding ascorbate biosynthesis enzymes. Each transcriptome bioproject was downloaded from the Sequence Read Archive (SRA) public database maintained by GenBank-NCBI. The following experiments were selected: fruit development (PRJNA485468; PRJNA533286; PRJNA668052), abiotic stress: heat, cold, salinity, osmotic and waterlogging (PRJNA525913; PRJNA646356; PRJNA793609), biotic stress (PRJNA588750; PRJNA476480), and exogenous application of phytohormones (PRJNA634831). Experimental conditions details employed in each transcriptome are described in Table S2.

Expression analysis of mostly three biological replicates was performed in four steps that are as follows: (1) removal of adaptors and low-quality sequences with Phred Quality Score < 20 using the BBDuk tool, available in the BBtools package (https://sourceforge.net/projects/bbmap/ (accessed on 8 November 2022)), (2) mapping of pepper short reads of each RNA-seq library to the deduced cDNA sequences using the default parameters of the aligner STAR v.2.7.10 [86], (3) raw count of all mapped reads to each gene member using the QuantMode parameter of STAR, and (4) normalization of raw count reads using the count-per-million (cpm) method according to the formula: (number of reads mapped to each cDNA × 10^6^)/(total number of reads mapped to each library) [87].

### 4.3. Searches for Regulatory Cis-Elements in Genes Promoter Regions

To corroborate the expression profile of DEGs associated with Asc biosynthesis pathways, we searched for regulatory cis-elements associated with light, phytohormone, and environmental alterations in the promoter region 1000 bp upstream of the translation initiation codon (ATG). The prediction of cis-action elements was performed using the PlantCARE database (http://bioinformatics.psb.ugent.be/webtools/p (accessed on 25 June 2023)) [88]. Then, the most abundant cis-elements were manually filtered and summarized using Tableau_Desktop-2023-2-2 Software (https://www.tableau.com/pt-br (accessed on 26 July 2023)).

### 4.4. Statistical Analysis

The normalized counts of reads for each treatment were submitted to one-way or two-way analysis of variance, using GraphPad Prism 8.0.1 Software. Then, means ± standard deviations of three biological replicates were subjected to Bonferroni’s or Tukey’s test, considering *p* < 0.05. To infer the differentially expressed genes (DEGs) between treatments, means were compared as follows: (1) for fruits at different developmental stages, each point was compared to the initial stage and (2) in the case of phytohormones and stresses, each treatment was compared to the control.

## 5. Conclusions

In summary, our results support that D-mannose/L-galactose pathway is the primary pathway of the ascorbate biosynthesis process, given its constitutive, ubiquitous, high expression profile and up/down-regulation observed in all organ and environmental stimuli investigated here. Expression profile supports evidence that myo-inositol and L-gulose pathways could play a significantly secondary role, and even an insignificant one, given its low- and even non-expression pattern exhibited by its genes. Based on gene expression patterns, *GGP2*, *GME1* and *2*, *GalLDH* from the principal pathway stand out as key regulatory genes, promising ascorbate metabolism’s manipulation. Finally, our findings shed light on relevant pathways, steps, and gene members under specific spatio-temporal conditions, paving the way for future biotechnological applications targeting the development of biofortified and stress-tolerant pepper cultivars and crops.

## Data Availability

The raw data that support this paper are publicly available in the SRA database from GenBank-NCBI under the following bioproject numbers: PRJNA485468, PRJNA533286, PRJNA668052, PRJNA525913, PRJNA646356, PRJNA793609, PRJNA588750, PRJNA476480, and PRJNA634831.

## Supplementary Materials

The following supporting information can be downloaded at: https://www.mdpi.com/article/10.3390/plants12193367/s1. Table S1. Sequences of all deduced 21 cDNAs and proteins related to ascorbate biosynthesis in *Capsicum annuum* L cv. UCD-10X-F1; Table S2. Description of RNA−Seq experiments used to evaluate the expression of genes involved in pepper (*Capsicum annuum* L.) ascorbate biosynthesis; Table S3. Means of CPM normalization values ± SD (standard deviation) of transcripts from Asc biosynthesis in pepper fruits of two varieties, ‘SJ11−3’ (higher Asc content) and ‘06g19−1−1−1’ (lower Asc content), at immature green (IG), mature green (MG), breaker (BR), and mature red (MR) developmental stages (Bioproject—PRJNA485468). One−way ANOVA analysis was performed followed by Bonferroni’s test. Statistical significance between fruits at 20 DAA compared to other stages are indicated by different lowercase letters, while capital letters represent significant differences between varieties, according to Bonferroni’s test (*p* < 0.05). Up− and down−regulated genes among developmental stages are indicated in red and green, respectively; Table S4. Means of CPM normalization values ± SD (standard deviation) of transcripts from Asc biosynthesis in pepper fruits of four varieties, HJ10−1, HJ11−3−1, CJ12−17−1, and 0622−1−3−2−1-3−1, at 30 and 50 DAA (Bioproject—PRJNA533286). One−way ANOVA analysis was performed followed by Bonferroni’s test. Statistical significance between developmental stages in each variety is indicated by asterisk (*), according to Bonferroni’s test (*p* < 0.05). Up− and down−regulated genes are indicated in red and green, respectively; Table S5. Means of CPM normalization values ± SD (standard deviation) of transcripts from Asc biosynthesis in pepper fruits at immature green (IG), breaking point (BP1), and ripe red (RR) developmental stages, as well as fruits in the breaking point 2 without nitric oxide treatment (BP2–NO) and treated with 5 ppm NO (BP2 + NO) for 1 h (Bioproject—PRJNA668052). One−way ANOVA analysis was performed followed by Bonferroni’s test as follows: BP1 and RR stages vs. IG and BP2−NO and BP2 + NO treatments vs. BP1 stage. Significant differences between treatments are highlighted by asterisk (*) at *p* < 0.05. Up− and down−regulated genes are indicated in red and green, respectively; Table S6. Means of CPM normalization values ± SD (standard deviation) of transcripts from Asc biosynthesis in pepper leaves under four types of stresses, heat, cold, salinity, and osmotic (mannitol), at 3, 6, 12, 24, and 72 h after treatments (Bioproject—PRJNA525913). One−way ANOVA analysis was performed followed by Bonferroni’s test, comparing the treatments in each time point with the control plants. Significant differences between treatments are highlighted by asterisk (*) at *p* < 0.05. Up− and down−regulated genes are indicated in red and green, respectively; Table S7. Means of CPM normalization values ± SD (standard deviation) of transcripts from Asc biosynthesis in pepper leaves of two genotypes, a tolerant (A188) and another sensitive (A122) to cold, at 1 h, 2 h and 12 h of cold stress, and in the recovery (Bioproject—PRJNA646356). Two−way ANOVA analysis was performed followed by Tukey’s test. Significant differences between control plants and the treatments are indicated by different lowercase letters, while capital letters represent significant differences between cultivars at *p* < 0.05. Up− and down−regulated genes between treatments are indicated in red and green, respectively; Table S8. Means of CPM normalization values ± SD (standard deviation) of transcripts from Asc biosynthesis in pepper roots of two genotypes, a susceptible (‘ZHC1’) and another tolerant (‘ZHC2’) to waterlogging, at 6 and 24 h of waterlogging stress, and 1 h post recovery (Bioproject—PRJNA793609). One−way ANOVA analysis was performed followed by Bonferroni’s test. Significant differences between control plants and the treatments are indicated by different lowercase letters, while capital letters represent significant differences between cultivars at *p* < 0.05. Up− and down−regulated genes between treatments are indicated in red and green, respectively; Table S9. Means of CPM normalization values ± SD (standard deviation) of transcripts from Asc biosynthesis in pepper leaves of two bell pepper near-isogenic lines (NIL), one infected with bell pepper endornavirus (BPEV+) and other BPEV−free, both further inoculated with PMMoV (Bioproject—PRJNA588750). One−way ANOVA analysis was performed followed by Bonferroni’s test. Statistical significance between BPEV−/Mock compared to other treatments are highlighted by asterisk (*) at *p* < 0.05. Up− and down−regulated genes are indicated in red and green, respectively; Table S10. Means of CPM normalization values ± SD (standard deviation) of transcripts from Asc biosynthesis in pepper apical stems infected with three tobacco etch virus strains (HAT, Mex21, and N) sampled at 7 and 14 h days post-inoculation (dpi) (Bioproject—PRJNA476480). One−way ANOVA analysis was performed followed by Bonferroni’s test, comparing the treatments in each time point with the control plants. Significant differences between treatments are highlighted by asterisk (*) at *p* < 0,05. Up− and down−regulated genes are indicated in red and green, respectively; Table S11. Means of CPM normalization values ± SD (standard deviation) of transcripts from Asc biosynthesis in pepper leaves treated with exogenous salicylic acid (SA), methyl jasmonate (MeJA), ethephone (ET), and abscisic acid (ABA) phytohormones sampled after 1, 3, 6, 12, and 24 h (Bioproject—PRJNA634831). One−way ANOVA analysis was performed followed by Bonferroni’s test, comparing the treatments in each time point with the control plants. Significant differences between treatments are highlighted by asterisk (*) at *p* < 0,05. Up− and down−regulated genes are indicated in red and green, respectively; Table S12. Prediction of putative cis−regulatory elements identified in the promotor region (1000 bp) of genes encoding enzymes of ascorbate biosynthesis pathways in pepper (*Capsicum annuum* L.).
