# Supplementary material for: Genome and Transcriptome Analyses of Genes Involved in Ascorbate Biosynthesis in Pepper Indicate Key Genes Related to Fruit Development, Stresses, and Phytohormone Exposures
Source: Plants (Basel). 2023 Sep 23;12(19):3367. doi: 10.3390/plants12193367 (PMC10574469; doi:10.3390/plants12193367)
Supplement: Supplementary file 1 [file plants-12-03367-s001.zip › Table S1.pdf]

Table S1. Sequences of all deduced 21 cDNAs and proteins related to ascorbate biosynthesis in *Capsicum annuum* L cv. UCD-10X-F1

>Ca_PMI1

GAAAATAAAAGAGTCAAAAATATTTTCTTTCCTACCAAATATAAGAAACCATTTTTCTCATTTTTCCTTT
TGTTTTCTTTCTTTCCTCTGTTTTCTGATTTTTCAGTTCTGACATCTACAATCTCCATTTCTTCCACATT
GAAACTGTTAACCAAAAAAGAAAAAAAAAAAAAAAAAGATAACGCAATGCGTGTGTCTACACTAGAGCAAT
GAAACGCTCGAAATCTGCTACAGCATTCCATGGAGGAGGTAAATGGTGGTGCATTGTTGATCAAGTTGAT
CTGTTGTGTTAAGAATTCAGATTGGGGTATCATTGGTCGTGAATCTACTGTTGCACGTCTTTATTATCGT
AATGCTCGCATCGGTATTGATCAGGATCATCCATATGCTGAATTTTGGATGGGAACACATGAGTCTGGTC
CGTCATATGTTCGGGGAGGCGAAGGAGGAGGAAAGGTGACGTTGAAGGAATGGATTGAAAGAAATCCGAG
TGTTCTTGGAGATACTGTTTTGAAGAAATGGGGTACCGATTTTCCCTTTCTCTTCAAGGTATTATCTGTT
GCCAAAGCTTTGTCCATACAGGCCCATCCAGACAAAGATTTGGCCACATCTCTGCATAAAGAGCAGCCTT
CTGTTTACAAGGATGACAATCATAAACCCGAGATGGCTTTGGCGCTGACGCAGTTTGAAGCTTTATGTGG
GTTTGTCAGTCTTGAGGAGTTTAAGGTGACTGTTCGTACTACACCGGAGATTGTGGAAGTGGTTGGTAAC
GCAAAAGCAGAGCAAGTTTTAAACTTGCATCAGCATGACGGGAAGGAGGAAATTAGATTACTGCTACAAT
CAGTATTTACTGACATAATGACAGTTCGCAAGGACGTGATTGCTGAAGTGTTATCCAAGCTGATTAGTCG
CCTAAACTTTGAAGGCCAGGCGAGGCACCTAACTGACAAAGAAAAACTGATTCTACAACTTGAGAAGCAG
TATCCAGATGATGTTGGTGTCTTATCTGCGTACTTGTTAAATTATGTGAAACTCAACCCTGGCGAAGCTT
TATATTTAGGCTCAAATGAACCTCATGCATATTTATATGGTGAATCTGTTGAATGCATGGCAAATTCAGA
TAACGTGATACGTGCCGGTCTAACTCCAAAGAACCGAGATGTTGAGATACTCTGTTCAATGCTCACATAC
AAACAGGGATTTCCTGAAATACTGAAGGGCACTGTGGTTAATCCATATACAATGAGATACCAACCTCCTT
TTGATGAATTCGAGGTGGACCGTTGCATTCTTCCCCAAAACTCAACAACTGTATTTCCTTCCATTCCCGG
TCCTTCCATTTTTTTGGTCGTGAGGGGAGAGGGAACGATGACAACATCATCAGATGAGGTAGTTGCCGAA
GGTGATGTCTTATTTGCATCTGCAAATACCAGTATTACTGTTGCAACCTTGTCTGGCCTGCATCTATATA
GAGCAGGAGTTAGCAGCAGGTTGTTTGAGTTGCATAAATAGTGTATTTAATTCTAACACTAGCTAGGATT
GATTCAAATACCAGAATCTTGCTGTATAATGAGCATATATATAGATGAATATTTTGAGTCAAAGTGACAT
TAGTGATAAATGGATTCTTCTCTGGTAAACATATTTTCTCCCAACTTTTTGATATCTACAAGAAAATGAC
CCTTTTTAGGAGAGTTGAACGGTAAAAACCACACCTAAACTATCACTTTTTCGCAAGTTTCATACCTCAA
ATATCCATTGTTCTCTTTGCCTATCT

XP_016570209.1 mannose-6-phosphate isomerase 1 [*Capsicum annuum*]

MEEVNGGALLIKLICCVKNSDWGIIGRESTVARLYYRNARIGIDQDHPYAEFWMGTHESGPSYVRGGEGG
GKVTLKEWIERNPSVLGDTVLKKWGTDFPFLFKVLSVAKALSIQAHPDKDLATSLHKEQPSVYKDDNHKP
EMALALTQFEALCGFVSLEEFKVTVRTTPEIVEVVGNAKAEQVLNLHQHDGKEEIRLLLQSVFTDIMTVR
KDVIAEVLSKLISRLNFEGQARHLTDKEKLILQLEKQYPDDVGVLSAYLLNYVKLNPGEALYLGSNEPHA
YLYGESVECMANSDNVIRAGLTPKNRDVEILCSMLTYKQGFPEILKGTVVNPYTMRYQPPFDEFEVDRCI
LPQNSTTVFPSIPGPSIFLVVRGEGTMTTSSDEVVAEGDVLFASANTSITVATLSGLHLYRAGVSSRLFE
LHK-

>Ca_PMI2

ATTCTATATTTCTTACATACAAATGCATACGAGAGTGACACCAATTCTTCGATAATCTCAACTTCCTTCA
TTCACTCACTGTCATCATCGTCACTTGTTTTGTCCACCCCCCCCCCCCCCCCCCGGTCCGCCTTCAAAA
AGCCTCCTCGAATGGAAAGTAGTAGCAGCAGCATTCAATTCCATACATTCTCTTTTTCTTCTTCCTTTAT
TTACATATTTCTACTTCTTTCTTTCTCTTTCCATGGAGGCTGATGCTCTCTCAATTACAACTATGCAAGA
CAGTTTTAAGGGGTTACTCAAGTTACTTGGTTCTGTCAAGAATTACGATTGGGGTTGTCCCGCTAAACAA
TCTTGTGTTGCACGTCTCTATAGCCTTAATTCTGGTGGAAATATTGATGAGAATCAGCCGTATGCTGAGT
TTTGGATGGGGACCCACGAGTCTGGACCGTCGTACGTGGTGGAAGGAGGAGGAAGAACTGAGAATGGTTA
TGCTGATGGCGAGGGAGTTAGAGACAGGTGTACTTTGAAGGAGTGGATTGAAAAGAACCCTAGTGTTCTT
GGTGAAACTGTTCTTACCAAGTGGGGTACCCAACTCCCCTTTCTCTTTAAGGTACTCTCTGTTGAGAAAG
CTTTGTCTATACAAGCTCATCCGGACAAGGATCTTGCAATTCTTCTGCATAAGGAGCAGCCACTAGTATA
CAAGGATGATAACCACAAACCTGAGATGGCTTTGGCTTTGACCAAATTTGAGGCCTTGTGTGGCTTCACA
AGTCTTGAGGAGCTTAAAGTGATTGTTCAGACTGTACCTGAGATTGTTGAAGTGGTTGGTAGTGCGCTTG
CAGAGCGAGTATGGTACTTGAACGATGATGATGGAGAGGAGAAAGTTAAATTAGTGCTCAGAAAACTATT
TACGGAGATGATGTCAGCTAGCAAGGATGTGATCAAGGAAGTTCTTGCTAAGCTGATTAGTCGTCTGAAC
ATAAAAAACAAGGTAAGGGAGCTGACTGACAAGGAACAGCTGATCCTAGGACTTGAGAAGCAGTATCCCG
CTGATGTTGGTGTTTTAGCCGCATTCTTGTTTAATTACGTGAAGCTTAATCCTGGTGAAGCTTTATATTT
GGGGGCAAATGAACCCCATGCATATGTATATGGCGAGTGTATCGAATGTATGGCAACCTCAGACAATGTG
GTACGCGCTGGCCTTACTCCCAAGCACCGGGATGTTAGGACACTGTGTTCAATGCTCACTTATAAACAGG
GTAACCCTGAAATTCTGTACGGTACGACAATAAATCCGTACACAACGAGATACCTTCCTCCTTTTGATGA

ATTTGAGGTGGATCGTTGCATTCTCCCCCCGCATTCAACTGTTGCCTTTCCTTCTGCTCCCGGTCCTTCC
ATATTTGTGGTCATGGGAGGAGAGGGAACAATGACCACATCAGCAGAAGTGATTGTTGCGGAAGGCGATG
TTCTATTTGCGCCTGCAAATACCAATATTACCATCGCAACTTCCTCTGGTTTACACTTGTATAGAGCAGG
AGTAAACAGCAGATTTTTTGAGGAATGATAGTTGTAAGCTTGTAGCCCCTTATGCTTACTAATAAAACAG
TGAATTTGTGTTCCAGGGGCTGTTCCATCTCGTATTATTAGCATGCTTCTATTAGTGTAGTTTTGTAAGT
AATAAACATCATTGGAAGAGTGGAAATTTTGTTGTACTTTGTCTCGTCTATTGTCTAATATTGCAAACGC
AAGAAAATGAATTGTACTCAAGTATGTGAACTAGGACTAGTGGTTGCGTAATGACTTGTTAGTATCCAAA
TTCCATCGTATTTCTCATATCCATTTTAAC

XP_016560755.1 mannose-6-phosphate isomerase 1 [*Capsicum annuum*]

MEADALSITTMQDSFKGLLKLLGSVKNYDWGCPAKQSCVARLYSLNSGGNIDENQPYAEFWMGTHESGPS
YVVEGGGRTENGYADGEGVRDRCTLKEWIEKNPSVLGETVLTKWGTQLPFLFKVLSVEKALSIQAHPDKD
LAILLHKEQPLVYKDDNHKPEMALALTKFEALCGFTSLEELKVIVQTVPEIVEVVGSALAERVWYLNDDD
GEEKVKLVLRKLFTEMMSASKDVIKEVLAKLISRLNIKNKVRELTDKEQLILGLEKQYPADVGVLAAFLF
NYVKLNPGEALYLGANEPHAYVYGECIECMATSDNVVRAGLTPKHRDVRTLCSMLTYKQGNPEILYGTTI
NPYTTRYLPPFDEFEVDRCILPPHSTVAFPSAPGPSIFVVMGGEGTMTTSAEVIVAEGDVLFAPANTNIT
IATSSGLHLYRAGVNSRFFEE-

>Ca_PMI3
GTTCAATCCCTCACTCTTTCTTGAATTGGACAATAACAGCAACATTTATGTGCTCCCTCTAATTCTTCTT
CTTTTTCATTTTTGTTTGAAGTAAAGCTACTGATTCTTTTTCTCTTCCCGTAGCATATTAAGCACTCTTA
GTCTTATCTCGAAAGTATTTGTTGTGACATCTTCGAATCACTTAACCTGTTAAACTCATATTGATCCTGT
TATTTTTTAATTGAGTTAATGAGTTCAATGGAGGGGAAAGGGAGGGTAGTGAAGTTGACAGGTTGCGTAA
AGAATTACGATTGGGGAAGAGCAGGGAAGGAATCACGTGTGGCGCGGCTGTATGCTTGTAATAGTGGTGA
CACTGTTGACTTAGAGAAGCCATATGCGGAATTTTGGATGGGGACTCATGATTCTGGGCCTTCATACGTA
GTAGAAGGAGCTGCTGGAGTATCTGAGAATGGATTGGGTAATAGTGGTGGTAGAGAGAGAAACAAGTTGA
CATTGAAGGAATGGATTGAAAACAACCCTATTGTTCTTGGCGAAAAAGTTGTGAACAAGTGGGGTACCAA
CCTTCCTTTTCTCTTCAAGGTACTATCTGTTGCAAAAGCTTTATCCATACAGGCCCATCCAGACAAAGAT
TTGGCATCTCGTCTGCATAGCGAGCTCCCTGATGTTTATAAGGATGATAATCACAAACCAGAGATGGCAT
TGGCATTAACAGAATTTGAGGCCTTATGTGGATTTATAAGTCTTGAGGAGCTGAAGTTGATTGTTCAAAC
TGTGCCTGAGATTGTCGAATTGGTGGGTACTGCACGCGCAGAGCAAGTATTGGAATTGAATGAGGATGGT
GGGAAAGAGAAAGGTAAATTAGTGCTAAAATCAGCATTTACTGAGCTGATGTCAGCAAGCAAGGATGCGG
TTGCTGAAGTGATAGCCAAGCTGATTAGTCGCCTAAATGTTAAAAATCAGGCAAGGGAGCTGACGGAGAA
AGAACAAGTGGTGCTGAGACTTGAGAAGCAGTATCCAGCTGATGTTGGTGTCTTGGCTGCATTCTTGTTA
AATTACGTGAAACTCAATCCTTGTGAAGCTTTATATTTAGGGGCCAATGAACCTCATGCTTATATATATG
GCGATTGTGTTGAATGCATGGCAACATCAGACAACGTGGTACGTGCTGGCCTAACTCCAAAGCACCGGGA
TGTTAAAACTCTGTGCTCAATGCTTACTTATAGACAGGGTTTTCCTGAAATTCTGCAGGGTACTGCTGTA
AATCCTCATGTTATGAGGTACATCCCTCCTTTTGATGAATTTGAGGTCGACCATTGTATTCTCCCCGAAC
AATCAACTGCTGAATTTCCTGCTATTCCTGGTCCATCCATTTTTATGGTCTTGGAGGGAGAAGGAACCGT
GACTACTTCATCAAACAAGATTATTTGTGAAGGTGACGTCTTTTTTGAAGCTGCAAATACCAGCATTACT
GTTTCAACATCCGCCGGTTTGCAATTATATAGGACAGGAGTAAATAGCAGGGTTTTTGATGAGTGAGTTG
TGTACGTATGGATAAAATAGTTCATCAAGTTACCTCTATACTAAAGTTGACTGATCCAACCTTGTAATAT
TAGCCTGTTCATGTCCTTTTTCATGCCATTTCTGTAAATCAGGTATACAAAATAACTATGAATATACTTC
TATAGTTGCCTCATTATACCAGTCTAATAACTATGAATATACTTCTATAGTTGCCTCATTATACCAGTCT
AATGGTTCACAGTCCAAGAGAGAGGA

XP_016558788.1 mannose-6-phosphate isomerase 2 isoform X1 [*Capsicum annuum*]

MSSMEGKGRVVKLTGCVKNYDWGRAGKESRVARLYACNSGDTVDLEKPYAEFWMGTHDSGPSYVVEGAAG
VSENGLGNSGGRERNKLTLKEWIENNPIVLGEKVVNKWGTNLPFLFKVLSVAKALSIQAHPDKDLASRLH
SELPDVYKDDNHKPEMALALTEFEALCGFISLEELKLIVQTVPEIVELVGTARAEQVLELNEDGGKEKGK
LVLKSAFTELMSASKDAVAEVIAKLISRLNVKNQARELTEKEQVVLRLEKQYPADVGVLAAFLLNYVKLN
PCEALYLGANEPHAYIYGDCVECMATSDNVVRAGLTPKHRDVKTLCSMLTYRQGFPEILQGTAVNPHVMR
YIPPFDEFEVDHCILPEQSTAEFPAIPGPSIFMVLEGEGTVTTSSNKIICEGDVFFEAANTSITVSTSAG
LQLYRTGVNSRVFDE-

>Ca_PMM

TCTTCCGGTCGGTGCAAATTTTGCGCTTCCTTTAACTAGTTTGTCCTCTTGATCAATAGATATATATAAG
TATTGTTTATAAATTGGAATATATATAGAGAGAGGGAGAGTCTAGTCAGAAGGTATTGGGTTAATCTCGT
AGTCGAATAAAGTGTTTTGTTTCTATTATTTGATTTGCTTCATCTCATATTTGTTAACTTTTCTGTTATA
TCATATTCAATACTGTTTGGCAGTTGAATGCAGGAGAGTTGGTGAGAAGTATACAAGGATAAAAATGGCC
GTAAGGAAACCTGGTTTAATTGCTCTGTTCGATGTTGATGGAACTCTTACTGCACCTCGCAAGGTATCTA
CTCCAGAAATGTTGAAATTCATGCAGGAACTTAGAAAGGTTGTTACTGTTGGAGTTGTCGGAGGGTCTGA
CCTTGTAAAGATATCAGAACAGCTTGGAAGTACAGTTACGAATGACTATGATTATTGTTTCTCTGAAAAT
GGCCTTGTAGCACATAAGGATGGCAAGCTTATTGGGAAACAGAGCTTGAAGTCATATCTTGGAGAAGAGA
AGCTCAAGGAATTTATTAACTTTACCCTCCATTACATCGCTGACTTGGATATTCCAATAAAGAGAGGAAC
ATTCGTTGAGTTCAGAAGTGGCATGCTAAATGTGTCACCTATCGGGAGGAACTGTAGTCAGGAAGAAAGG
GATGAATTTGAAAAGTATGACAAGGTACAAAAGATACGTGAAACAATGGTATCAGTGCTCAGAGAAAAGT
TTGCACACTTTAATCTCACCTTCTCCATTGGAGGCCAAATTAGTTTCGATGTTTTCCCCCAAGGCTGGGA
CAAAACTTATTGTTTGAGATACCTTGAAGAGTTTAATGAAATTCACTTTTTTGGGGACAAAACATACAAG
GGAGGAAATGACCATGAGATCTACGAGTCTGAGAGAACGGTTGGTCACACAGTTACTAGCCCGGAGGACA
CATTGAGTCAGTGTTCTGTTCTATTCCTCGGCAAGGATAATGGAAGTTCTTGAGCTTGTTGCAGTTTCTC
AATACGTGATTTTATGCTGGTAACCTATGCATACGGCAGAAATGAATAATTGCAGTACTTCTTGTATTGA
ATTTACTCATCAAAATTATGATATGAATGTAGAACTTTCTTGTGTATATTTCCGTTCCAACGACTATTTT
TTACCTCATTCTCAAAACGCCACAATATTATTTTTCTTCAGGGGTGTATGATTGATCTCAACATAACC

XP_016538367.2 phosphomannomutase [*Capsicum annuum*]

MAVRKPGLIALFDVDGTLTAPRKVSTPEMLKFMQELRKVVTVGVVGGSDLVKISEQLGSTVTNDYDYCFS
ENGLVAHKDGKLIGKQSLKSYLGEEKLKEFINFTLHYIADLDIPIKRGTFVEFRSGMLNVSPIGRNCSQE
ERDEFEKYDKVQKIRETMVSVLREKFAHFNLTFSIGGQISFDVFPQGWDKTYCLRYLEEFNEIHFFGDKT
YKGGNDHEIYESERTVGHTVTSPEDTLSQCSVLFLGKDNGSS-

>Ca_GMP1
TGTTGGCCGTGTGTACATGCTATTCTAGTATATATATATATATATCCATCCATTACAAGTGGCCTTTTGT
ACTCCACCGTCCACCGATCTTCTAACACCACACAACACAAAACTCCCTAAATACACTCCCCTTCGTTGTT
GTTTGATTTGGCTGTCAACATCTCTGACTAGAGTCCACTCTTTCTGCAATTCAGTCTTTGAACTTTTTCG
ATCTGCAATTCAAAAAGGAACTTTAATAGCTCAAGATGAAGGCACTTATCCTTGTTGGAGGGTTCGGTAC
TCGGCTCAGGCCACTCACCCTCAGCGTCCCAAAGCCACTAGTTGAATTTGCCAACAAACCGATGATTTTG
CATCAGATCGAGGCTCTCAAGGCCGTCGGAGTAACCGAAGTTGTACTGGCTATTAATTACCAACCTGAGG
TGATGCTGAACTTCTTGAAAGAATTTGAGGCAAGCCTTGGAATCAAGATCACCTGTTCTCAAGAAACTGA
ACCACTTGGCACTGCTGGTCCCCTTGCTTTGGCTAGAGACAAGCTGATTGATGATTCTGGTGAACCATTT
TTTGTTCTTAATAGTGATGTTATCAGTGAATATCCTTTTAAGGAGATGATTGAATTCCATAAATCCCATG
GAGGTGAGGCTTCTTTGATGGTGACCAAGGTGGATGAGCCTTCTAAATATGGTGTTGTCGTCATGGAAGA
ATCCACAGGGCAAGTAGAGAGATTTGTGGAGAAGCCAAAGTTATTTGTTGGCAACAAGATCAATGCTGGA
TTTTACCTGCTGAACCCTTCTGTTCTAGACAGAATTCAATTACGGCCAACATCAATTGAGAAAGAGGTTT
TTCCGAAAATTGCAGCAGAGAAGAAACTGTATGCCATGGTGCTACCTGGATTTTGGATGGACGTTGGCCA
GCCAAAAGATTACATTACTGGCCTCAGGCTCTATTTGGATTCTTTAAAGAAGAACTCTTCACCTAAATTG
GCTTCAGGATCACACATTGTCGGAAATGTCATTGTCGATGAGTCTGCCAAAATTGGAGAGGGTTGTTTGA
TAGGACCAGATGTTGCAATTGGTTCTGGATGTGTGATTGAGTCTGGAGTTAGACTCTCCCGTTGCACTGT
GATGAGAGGAGTCCGCATCAAAAAACATGCATGCATTTCAGGTAGCATCATTGGCTGGCATTCTACTGTT
GGACAATGGGCTCGTGTCGAGAACATGACCATTCTTGGAGAAGATGTCCATGTTTGTGATGAAGTTTACA
GCAATGGAGGTGTGGTTTTGCCCCACAAGGAGATCAAATCTAGCATATTGAAACCTGAAATAGTGATGTG
AGGATATCATGTTTGTTAGCACTAGGATAAAGTTATGTATTCGGTGTCTTTATGATGTCTTTTTCATCAC
CTGTTCCTTTTTTGGTTGGTTTCCAGTTTTCCTCTTCTTCAAGTGTCAATGAAAATTTGTGTGATAACTT
TTCTGCTAAATTGTAGTTATGTTATGAAGGGTATGAGATATTGTAGAGACTAGAGACTGGAAATGTGTAA
TTTTTCCTATGTATAGATAAATAAAATTTCTCCTTA

XP_016565025.2 mannose-1-phosphate guanylyltransferase 1 [*Capsicum annuum*]

MKALILVGGFGTRLRPLTLSVPKPLVEFANKPMILHQIEALKAVGVTEVVLAINYQPEVMLNFLKEFEAS
LGIKITCSQETEPLGTAGPLALARDKLIDDSGEPFFVLNSDVISEYPFKEMIEFHKSHGGEASLMVTKVD
EPSKYGVVVMEESTGQVERFVEKPKLFVGNKINAGFYLLNPSVLDRIQLRPTSIEKEVFPKIAAEKKLYA
MVLPGFWMDVGQPKDYITGLRLYLDSLKKNSSPKLASGSHIVGNVIVDESAKIGEGCLIGPDVAIGSGCV
IESGVRLSRCTVMRGVRIKKHACISGSIIGWHSTVGQWARVENMTILGEDVHVCDEVYSNGGVVLPHKEI
KSSILKPEIVM-

>Ca_GMP2

CTTGAGCCGGGGGTCTATCGGAAACAACCTTTCTACTTCTTTAGAGGTAGAGGTATGAACTGCGTACATC
TTACCCTCCCCAGACCTCACTATGTGGGAATACACTGGGTTTGTTGTTGTAATCTTACATCTCCTTC
GTCCTTATATTACAATTCCACAATGATGCAAATGGTTATTTCTTGATATTTTGTTTTTCTGCTGCAGGCA
GCCTTTTGGTTTTCATTATGAAGGCACTCATCCTTGTAGGAGGTTTTGGAACAAGGCTGAGGCCACTGAC
CCTCAGTGTGCCAAAGCCCCTAGTTGACTTTGCTAACAAACCCATGATACTCCATCAGATTGAAGCACTA
AAAGCCATCGGAGTGACCGAAGTAGTTTTGGCAATTAACTATCAACCAAAGATAATGCAAAATTTCCTGA
AAGAGTTTGAGAAAAAGTTAGATCTGAAGATCACTTGCTCGCAAGAGACAGAGCCTTTGGGTACAGCAGG
GCCACTGGCTTTAGCTCGTGATGTACTAAGAGATGACTCTGGGGAGCCCTTTTTCGTCCTCAATAGTGAT
GTCATATGTGACTACCCATTGAAAGAAATGATTGAGTTTCACAAGTCCCATGGTGGTGAAGCTTCCATTA
TGGTTACAAAGGTTGATGAACCATCAAAATATGGTGTAGTGGTCATGGAAGAAGTGACGGGGAGAGTTGA
AAAATTTGTGGAAAAACCCAAAGTATTTGTGGGTAACAAGATCAATGCTGGAATTTACTTGTTGAACCCT
TCTGTTTTGGATCGGATTCAGTTGAGACCAACCTCAATCGAGAAGGAAGTCTTCCCCTGCATTGCAGCAG
AGAAGAAGCTCCATGCCATGGTCCTACCAGGCTTCTGGATGGACATTGGTCAGCCGAGGGATTACATTAA
AGGCTTGAGACTATACCTGGATTCCTTGAGGAAAAAGTCATCACCTGATCTGGCTGTTGGACGGCATATT
CTTGGAAACGTATTACTAGACGAGAGTGCTGTAATAGGCGATGGATGTCTTATCGGTCCAGATGTGGCTA
TTGGACCTGGATGTGTTATTGAATCAGGAGTTAGGCTTTCACGTTGTAGTATAATGCGTGGTGTACGAAT
TAAGAAGCATGCCTGCATCTCAAGTAGCATTGTTGGATGGCACTCAACTGTTGGTCGGTGGGCTCGGGTA
GAGAACATGTCAATCTTGGGAGAAGATGTTCATGTTGGAGATGAAGTCTACAGCAACGGAGGTGTTGTTC
TACCCCATAAAGACATACAATCCAGCATTCTGGAGCCAGAGATTGTCATGTAACATTGTGGTATGATGTG
GTTACATTCTATAAAATAAAGCAATATCTGTTCTCAGAAAATATTCTTAGATTTCCAGTTGTGTTGTCAT
TTCAATCATCAGAAATGTGCAAGTTAGAAAACTTAGGTCTCTCTGTATTTTGAAATAATGGTTAGGGCCC
TGATCTATTCAAGGTTTTGCAGTTGTCAATCACATTGGTTGGATGCTATAGTTATTTGGAGGTGATTAAC
TTCTATGTTATATC

XP_016542353.1 mannose-1-phosphate guanylyltransferase 1 [*Capsicum annuum*]

MKALILVGGFGTRLRPLTLSVPKPLVDFANKPMILHQIEALKAIGVTEVVLAINYQPKIMQNFLKEFEKK
LDLKITCSQETEPLGTAGPLALARDVLRDDSGEPFFVLNSDVICDYPLKEMIEFHKSHGGEASIMVTKVD
EPSKYGVVVMEEVTGRVEKFVEKPKVFVGNKINAGIYLLNPSVLDRIQLRPTSIEKEVFPCIAAEKKLHA
MVLPGFWMDIGQPRDYIKGLRLYLDSLRKKSSPDLAVGRHILGNVLLDESAVIGDGCLIGPDVAIGPGCV
IESGVRLSRCSIMRGVRIKKHACISSSIVGWHSTVGRWARVENMSILGEDVHVGDEVYSNGGVVLPHKDI
QSSILEPEIVM-

>Ca_GME1

TCCTAGTGTATTCAGATAAAATTTTATTCTTGATGTTCATTTTATGTGCTTTACACCTAGAGTTTGTCCT
TCATCCCTACTTGATGTATGTCTTTGACTTCTTGTTTCGGTATTAACCACAAGGAGATTCACTTCATTAA
TGTTGTTCCTTCTTTTTGCAAGTATTTTGATAATCCGGTGAGTTTGATTCTGTTATGTTCCTAACTAATG
CATCTAGACTCTCGAGATTGGCTCCATTTGACGTAGTATTCCCCACATTTTCCTTTTAATGATGGTATGT
TGTGCACAGAATGGGAACTTCTGTTGAAACTAAGTACGGGGAATACACATATGAAAACCTTGAGCGGGAA
CCTTACTGGCCTTCAGAAAAGCTTCGGATATCTATTACAGGAGCTGGTGGATTCATTGCCTCACACATTG
CAAGGCGTCTGAAGACCGAGGGGCATTACATTATTGCTTCTGACTGGAAGAAGAATGAGCACATGTCTGA
GGACATGTTTTGTCATGAGTTCCATCTTGTTGATCTCAGGGTTATGGATAACTGTTTAAAAGTCACAAAA
GGAGTTGATCACGTGTTCAATCTTGCTGCCGATATGGGAGGTATGGGCTTCATTCAGTCTAACCACTCGG
TGATCATGTATAACAATACTATGATCAGCTTTAACATGATGGAGGCTTCAAGAATAAATGGTATTAAGAG
GTTCTTTTATGCATCCAGTGCTTGCATTTACCCTGAATTTAAGCAGTTGGAAACTAACGTGAGCTTAAAG
GAGTCTGATGCTTGGCCTGCAGAGCCTCAAGATGCTTATGGCTTAGAAAAGCTAGCAACAGAGGAGCTAT
GTAAGCACTACAACAAGGACTTCGGAATTGAATGTCGCATTGGACGTTTCCATAACATTTATGGCCCATT
TGGAACATGGAAAGGTGGACGCGAGAAAGCTCCAGCAGCTTTTTGTAGAAAAGCCCTCACTTCCACTGAC
AAATTCGAGATGTGGGGAGATGGAAAGCAAACTCGATCTTTCACCTTCATTGATGAGTGTGTTGAAGGTG
TTCTGAGGTTAACGAAATCAGACTTTAGAGAGCCTGTGAACATCGGAAGTGATGAGATGGTAAGCATGAA
TGAGATGGCAGAGATAGTCCTCGGCTTTGATGGCAAGAACCTTCCTATCCATCACATCCCGGGACCAGAA
GGTGTGCGTGGTCGAAACTCTGACAACACACTCATAAAAGAAAGGCTTGGGTGGGCTCCTACGATGAAGT
TGAAGGATGGGTTGAGAATTACATATTTCTGGATCAAGGAACAAATTGAGAAAGAGAAAGTGCAGGGTTC
CGATGTGTCCGCTTATGGGTCATCCAAAGTTGTGGGAACACAAGCTCCGGTTGAATTGGGCTCCCTGCGT
GCTGCCGATGGCAAAGAGTGAAGTAGATAAAGCCAAGTGACTATCATTCAGTATGCATGAAAGCCATCTT
GTTAGTTCCACCATGTTTTGTCGCGTGGAATGTTTACCTATTGATATATTTAATATTATGTGCTAAAGCA
GTTTTATTTTTTCGCTACACCATATTGCACCTGCCAAATAATGCCTAGAGATGTAGTCAGCATATGCTTG
CATTTATGGTATTTTCTGAGTTTGTATGAGACAGCCATTTTCTATCAATCTATCGTTTTCTTGCTCCTT

XP_016566587.1 GDP-mannose 3,5-epimerase 2 [*Capsicum annuum*]

MGTSVETKYGEYTYENLEREPYWPSEKLRISITGAGGFIASHIARRLKTEGHYIIASDWKKNEHMSEDMF
CHEFHLVDLRVMDNCLKVTKGVDHVFNLAADMGGMGFIQSNHSVIMYNNTMISFNMMEASRINGIKRFFY
ASSACIYPEFKQLETNVSLKESDAWPAEPQDAYGLEKLATEELCKHYNKDFGIECRIGRFHNIYGPFGTW
KGGREKAPAAFCRKALTSTDKFEMWGDGKQTRSFTFIDECVEGVLRLTKSDFREPVNIGSDEMVSMNEMA
EIVLGFDGKNLPIHHIPGPEGVRGRNSDNTLIKERLGWAPTMKLKDGLRITYFWIKEQIEKEKVQGSDVS
AYGSSKVVGTQAPVELGSLRAADGKE-

>Ca_GME2
GGGTGTTTCTGTTTATGTCACTGTAGTCCGTAAGTTCCTCTTTTTATACTCAAATTTGTTGGTGCATAGA
ATTGAGAAGAAACACATGGTTGATAATTCAACATTAGAATGTTAACAATTGTGAATATAGCCTGCTCCTA
GTGAGTTAATTAACTAGTTAGTGTTTTTTTTTCTAGTGAAACATCATTTAACTTGATTGTAGTTTTTCTC
AGAATGGGAAGCTCTGGTATTGACTATGGTTCTTACACTTATGAGAATCTTGAGAGGGAGCCTTACTGGC
CATCTGAGAAGCTCCGTATTTCCATTACTGGGGCTGGAGGATTTATCGCTTCCCATATTGCTCGTCGTTT
GAAGAGCGAGGGCCACTACATAATTGCTTCCGATTGGAAGAAGAATGAGCACATGACGGAGGATATGTTC
TGTCATGAGTTTCATCTTGTGGATCTTAGGGTTATGGCTAATTGCTTGAAGGTAACAAAAGGAGTTGACC
ACGTCTTCAACCTCGCTGCTGATATGGGTGGCATGGGTTTCATTCAGTCCAACCACTCAGTTATTTTCTA
TAACAACACTATGATCAGTTTCAACATGATGGAAGCTGCTAGGATTAACGGTATCAAAAGGTTCTTCTAT
GCATCTAGCGCTTGCATTTATCCCGAGTTCAAACAACTTGAAACAAATGTCAGCCTGAAAGAAGCTGATG
CTTGGCCTGCAGAGCCTCAAGATGCTTACGGCTTGGAGAAGCTTGCCACTGAAGAATTGTGCAAACATTA
TAACAAGGATTTTGGAATTGAATGTCGCATTGGAAGGTTCCATAATATCTATGGACCTTTTGGAACTTGG
AAAGGTGGAAGGGAAAAAGCCCCTGCCGCTTTTTGTAGAAAAGCCCAAACCGCAACTGATAAGTTTGAAA
TGTGGGGAGACGGACTTCAAACACGTTCATTCACCTTCATTGATGAGTGTGTTGAAGGAGTTCTCAGATT
GACAAAATCTGACTTCCGGGAGCCAGTGAACATTGGAAGTGATGAGATGGTCAGTATGAATGAGATGGCT
GAGATGGTTCTCAGCTTTGAGGACAAGAAGCTTCCCGTCCACCACATTCCTGGCCCAGAAGGTGTCCGTG
GTCGCAACTCAGACAATATGCTGATAAAAGAGAAGCTTGGTTGGGCTCCGACAATGAAATTGAAGGATGG
TCTGAGAATTACTTACTTCTGGATCAAGGAGCAGATCGAGAAAGAGAAATCTCAAGGAGTTGACACAGCA
ACCTATGGATCCTCCAAGGTGGTAGGCACCCAAGCTCCAGTTGAGCTTGGTTCCCTTCGTGCTGCTGATG
GCAAGGAATAAGTCCATCCCTTCTGTTACCGTTTCAATTGGAAGCCAATCCCTGCTATAGTATTTGCTAC
ATTATGTCATATGGCATCGTAGAAGAACGTGTTACATTCTGATAATTGTTGGCTTTTCTTGGATTTGAAC
ATGTCGATGTAATCTAAACCCCCTCAGCCTTGTTGAGGGTATAGTTTAATGCGTCGGCTATATTTATCAG
TTGTTCGAGAAGATCTATATGAGAATTGAATAACTGGCAGTGGATCCTCCCTCTCTTTTTACCTAGTTCA
GGATCTTGCTGTTATGCT

XP_016538382.1 GDP-mannose 3,5-epimerase 1 [*Capsicum annuum*]

MGSSGIDYGSYTYENLEREPYWPSEKLRISITGAGGFIASHIARRLKSEGHYIIASDWKKNEHMTEDMFC
HEFHLVDLRVMANCLKVTKGVDHVFNLAADMGGMGFIQSNHSVIFYNNTMISFNMMEAARINGIKRFFYA
SSACIYPEFKQLETNVSLKEADAWPAEPQDAYGLEKLATEELCKHYNKDFGIECRIGRFHNIYGPFGTWK
GGREKAPAAFCRKAQTATDKFEMWGDGLQTRSFTFIDECVEGVLRLTKSDFREPVNIGSDEMVSMNEMAE
MVLSFEDKKLPVHHIPGPEGVRGRNSDNMLIKEKLGWAPTMKLKDGLRITYFWIKEQIEKEKSQGVDTAT
YGSSKVVGTQAPVELGSLRAADGKE-

>Ca_GGP1
GATTATTGCCGTCGTTGAAACAAGAAATTCAATCACGGCTATTCTTGGATCATTCGTTTTTATCTTCACA
ATTCATCATCAACAAGGCAAAGTGTTGATCGATAATCCAACACTTCTGGTGGACGATAAGCACCGTACCC
CTTCCCCTCACGGCGGTAGGGGTGCCTCTCCTGCTGAAGGCGGTTGCCCTTCCGATCTCCTCTTCCTCGC
CGGCGGCGGTCCACCTCTCTCTTAGACTTTTCTTGTAAGAATTGTTATAAATATTATTTCTTTTGAATTA
TTCATTGAAGAACAGGAGCAATGTTGACTATAAAGAGGGTGCCCACAGTAGTTTCAAACTACCAAGAGGA
CGTTCTTCTTGAAAGTAACGTCGTTGGTTGTGGCAGAAAGTGCCTTGGAAAATGCTGCTTGCCTGTTTCC
ATGCTTCCTCTATACTCATTCAAGAATGATGACAATGAGCCAATGGAACATAATATTCATACCTTACCTG
AGGAGGAGTATCAGATATCATTCTTAAACAATTTGCTGTTAGGTCTATGGGAGGAGCGGATGAGCCAGGG
ACTATTTCGATATGACGTGACAACCTGTGAAACAAAGGTCATTCCTGGGAAATGTGGTTTTATTGCACAG
CTGAATGAGGGGCGCCACCTAAAGAAGCGCCCAACAGAGTTTCGCATTGATAAGGTTCTTCAGCCTTTTG
ATGAGAACAAATTCAACTTTACCAAAGTGGGACAGGACGAAGTGCTTTTCAGGTTTGAACCAAGCACTGA
CTACAAACGGCGTTACTTTTCGGGCATGGGAGTAGACGCTGGTATTTCACCTAGTATTGTTGCTATCAAT
GTGAGCCCTATTGAGTATGGCCACGTGCTTTTGATACCTCGAGTTCTTGATTGTTTACCTCAGAGAATTG
GTCGTGATACTTTCACAATTGCTCTCCATTTTGCCAGAGAAATGGCAGATCCCTTCTTTAGGGTAGGTTA
TAACAGTTTGGGCGCTTTTGCTACCATTAACCACCTCCACTACCAGGCATATTACTTGTCAGTTCCATTT
CCAGTTGAGAAAGCACCAGTACGGAAGATACTGGCAAGGAAGGGGCTGGGTGGCGCTGGAGTGATTGTTT
CGAAGTTATTAAATTACCCTGTGAGAGGTTTTACTTTTGAGGGAGAAAATGGAAGTACCATCCGTGATTT

GTCTGAGGTTGTTGTCAATTCCTGCATCTCCCTTCAGAATAAAAACATCCCTTTCAACATTCTCATTGCT
CAGTGTGGAAAGAAGATTTTTCTGTTTCCCCAGTGTTATGCAGAGAAGCAAGCTCTTGGAGTTGTAGACC
AAGGGCTCCTCGATACTCAGGTGAACCCCGCTGTGTGGGAAATTAGTGGACATATGGTGCTTAAGCGAAC
AAAGGATTACGAAGATGCATCAGAGAAACTTGCATGGAAACTTCTTTCTGAGGTTTCCTTATCGGAGGAG
AAATTTGAAGAGGTGAAGGGCTATATTTCTGAAGCTGCTAATTTGGAAGAAATGGAGGAGGATAAAAGCA
TCAACCCAGATAAGGAAATTCCAGATTCTCCTGGTCCGAGAGTGGCCTCACATGTGCCTCAAGATTGTTT
GGTGCTACACTGAAGAATTGAGGCATGCTCTTGCAGGATCTAATAAAGGTTGAAGTATCTAGTGTATGGT
TGATACTTTATGTGCTGTCTTTGCAGCTTCTTGTTTGATATTGTCTTGTTAGTTGGAGTTGGTAAACTAA
TCCCCGAATAGCGTTTTTTTTCTTTTTTTGGTTAACCCGAATAGCGTTGTTAATGCCTGCGAGCTGCGGC
CTTTGGCAGGGTTGTTTAATAAATATTGCTGTACCTTCTTTGTTGGTTTTATAACTGATCATATTAGCAA
ATGTTCCTAAT

XP_016560845.2 GDP-L-galactose phosphorylase 2 isoform X1 [*Capsicum annuum*]

MLTIKRVPTVVSNYQEDVLLESNVVGCGRKCLGKCCLPVSMLPLYSFKNDDNEPMEHNIHTLPEEEYQIS
FLNNLLLGLWEERMSQGLFRYDVTTCETKVIPGKCGFIAQLNEGRHLKKRPTEFRIDKVLQPFDENKFNF
TKVGQDEVLFRFEPSTDYKRRYFSGMGVDAGISPSIVAINVSPIEYGHVLLIPRVLDCLPQRIGRDTFTI
ALHFAREMADPFFRVGYNSLGAFATINHLHYQAYYLSVPFPVEKAPVRKILARKGLGGAGVIVSKLLNYP
VRGFTFEGENGSTIRDLSEVVVNSCISLQNKNIPFNILIAQCGKKIFLFPQCYAEKQALGVVDQGLLDTQ
VNPAVWEISGHMVLKRTKDYEDASEKLAWKLLSEVSLSEEKFEEVKGYISEAANLEEMEEDKSINPDKEI
PDSPGPRVASHVPQDCLVLH-

>Ca_GGP2
GCTATACACAAAGTAAACCGTCGCCGACCACTCTTACATGTTCGGGCAGTACGACGTAAGGGTTGTGTAA
CGGCTACTAATCCTGCTCCCCACGGTGGACGTGGCGCTTTGCCATCTGAAGGTGGTAGTCCTTCCGACCT
CCTCTTCCTTGCCGGCGGCGGTTCCATTTTCTCCTCCTACTACTAGATATAGCTTGCTTTTACTTACCTA
CCTTAGATCTCTAGATTATTCCGTACAGTTTAATTAAGGTTGTTCTACCTAGTAAAAATTCGAAGATAAA
ATCACAAAAAAGAAGTAAAGATGATGCTTAAGATTAAGAGGGTTCCTACACTTGTCTCTAACTTCCAAAA
GGATGAAGATGAAATTGGTGCTCGTGGTGTCGGCTGTGGTCGGAATTGCCTTAGGAACTGCTGCCTTCCA
GGTTCAAAGCTGCCATTGTATGCTTTCAAAAATTTGAGCTACGGAAAGTTTGTTGCTGATGAAACCATGG
AACCTCCCGTTGACTTTCTGGAATCCCTTCTTCTTGGGGAATGGGAGGATCGTCAGCAGAAAGGCCTCTT
TCGCTATGATGTCACTGCCTGCGAAACCAAGGTGATTCCTGGAGAATATGGTTTCATTGCTCAACTGAAT
GAGGGAAGGCACCTCAAGAAGAGGCCAACTGAGTTTCGAGTTGATAAGGTGCTGCAGCCTTTTGATGGAA
GCAAGTTCAACTTCACTAAGGTTGGACAGGAAGAGTTGCTCTTTCAGTTTGAAGCAAGTGATGAAGATGA
AGTCCAGCTCTACCCAGATGCACCCATTGATGCTGAGAAATCTCCAAGTGTTGTTGCCATCAATGTCAGT
CCCATTGAATATGGACACGTGCTTTTGATCCCTAAGGTCCTTGAATGCCTTCCCCAGAGGATCGACAGGG
ACAGCTTCTTGCTTGCGCTGCATATGGCTGTGGAAGCAGCAAACCCATACTTCCGATTGGGTTACAACAG
TTTGGGTGCATTTGCTACCATCAACCATCTTCACTTTCAGGCTTATTACTTGGCTGTGCAATTCCCCATT
GAGAAGGCCCCAACTCAGAAGATATCCGTTACTGGTGCTAGAGTGAAGATATCGGAGATGCTGAATTACC
CAGTTCGAGGACTTGTCTTTGAGGGTGGAAATACTTTGGAGGATTTGGCCAATGTTGTCTCAGATTGTTG
CATTTGCCTGCAAGAGAACAACATCCCCTACAATGTTCTAATCTCTGATTCTGGAAAAAGGATATTCCTT
CTCCCACAGTGCTATGCAGAGAAACAAGCTCTTGGAGAGGTCAGCCCTGAACTCCTCGACACCCAAGTCA
ATCCTGCGGTTTGGGAGATTAGCGGACACATGGTCTTAAAGAGGAAGGAGGATTACGAGGGTGCCACCGA
GGCAAATGCCTGGAGGCTTCTTGCTGAGGTCTCACTCTCCGAAGTGAGGTTCCAAGAGGTTACTGCTCTC
ATCTTTGAAGCCATTGGTCTCAGTGTTGAAGAGAATGAGCACGCCCCTGAAGGTTCTCCTGAGGATCTAG
ATGTCACACCACCTCAGCCCATGGAGGAAATCGATGGTCTCAGCACCCACACTACCATGGTTCCCGTCTA
GGGCTTCCCATGGCCAAGCTCTGGTGTTGTTCCCGTCTTTATCTCTCTCTTTGCTGGCTGCCCTTGTGAA
ATATCACTAAATAAGGCAAAGCCATGTTGTGTTGATGAATTTGGTCTGTTCTTCTATGAATTGAAACCTT
GTTTCTTATTGCTTTTAGGTTGGTCTGTGAATTGACCTTTGGTACGACGTTTGTTGTTCTTAATTCTAAG
ACTATTATTTGCTAATGGAAGTTGGATTTCCTCTTTTTGTTGTTAAAACATGTGCTTCTGATGTATAGTT
ACAGAGTA

XP_047269959.1 L-galactose phosphorylase 1 [*Capsicum annuum*]

MMLKIKRVPTLVSNFQKDEDEIGARGVGCGRNCLRNCCLPGSKLPLYAFKNLSYGKFVADETMEPPVDFL
ESLLLGEWEDRQQKGLFRYDVTACETKVIPGEYGFIAQLNEGRHLKKRPTEFRVDKVLQPFDGSKFNFTK
VGQEELLFQFEASDEDEVQLYPDAPIDAEKSPSVVAINVSPIEYGHVLLIPKVLECLPQRIDRDSFLLAL
HMAVEAANPYFRLGYNSLGAFATINHLHFQAYYLAVQFPIEKAPTQKISVTGARVKISEMLNYPVRGLVF
EGGNTLEDLANVVSDCCICLQENNIPYNVLISDSGKRIFLLPQCYAEKQALGEVSPELLDTQVNPAVWEI

SGHMVLKRKEDYEGATEANAWRLLAEVSLSEVRFQEVTALIFEAIGLSVEENEHAPEGSPEDLDVTPPQP
MEEIDGLSTHTTMVPV-

>Ca_GPP1

ATGGCAATACATTCGTTTATAATAATGCAAAACCCAATTTAGCTAGAATTAGAAAATATATTCTTATAAT
TTTTAAACTAAAAATTTTATTTAGACGTGAATTAAATAAATATCAAAAGCTAAAGAGATAAATTTTATTT
TCTGCTCAAAATTTGAATTATTTTTCCAACGTAAACAAATTTTATGACCTATATATAATATTCTTCTTTC
ATGTGCTACGTAAAAATATTTATTTATACATCATATTGTCCCTTTCTCTTCTCAATCACTCAATTTAAAA
AAATATTTTTTTAAGATGGCACAAAATGGTGCAGTTGAAGAGTTTCTTGATGTTGCAGTTGAAGCAGCAA
AGAAAGCTGGAGAGATAATTCGTGAAGGATTCTACAAGACTAAGCATGTAGAGCACAAAGGAGTGGTGGA
TTTAGTCACAGAGACTGATAAAGCATGTGAAGATCTCATTTTTAATCATCTCAAAGAGCATTTCCCGAGC
CATAAGTTCATTGGTGAAGAAACAACTGCTGCTTCTGGAAATTTTGAGCTGACTGATGAACCAACTTGGA
TAGTTGATCCACTTGACGGAACCACTAACTTTGTGCACGGGTTCCCTTTTGTCTGCGTGTCTATTGGTCT
CACAATTGGTAAGAAACCAACGGTTGGTGTTGTTTACAACCCAATTATTGATGAGCTTTTCACTGCAATC
GAGGGTAAAGGTGCTTTTTTCAACGGGAAGCCTATCAAAGTATCTTCACAGTCCGAACTTGTGAAGGCTC
TTCTTGCTACAGAGGCTGGAACAAAGCGGGATAAATTAACTGTTGATGCTACTACAGGGAGAATCAATAG
CTTGCTTTTTAAGGTCAGGTCCCTCAGGATGTGTGGTTCTTGTGCATTAAATCTCTGTGGAGTGGCATGT
GGAAGGCTTGATCTCTTCTACGAACATGAATTTGGAGGCCCTTGGGATGTGGCAGGTGGTGCTGTGATAG
TGAAAGAAGCTGGAGGTTTCGTGTTTGATCCATCTGGTTCAGAACTTGACCTCACAGCTCGACGGGTAGC
TGCTACAAACGCTCATCTCAAGGACGCATTTATCAAGGCCTTGAATGAATCAGAATGAGATGGAAAATGG
AAAGAACTTACTGTCAGAACCGGTTCTCTTTCATTATTAAATTGCATAAAGCTACTCAATTCTTGCCTCC
ATCTCTCAGGATTCAAGCGTTATTTAGCCTAACAACAATCAAATTCCTGCCATTTACGGCTGGTTGTTAA
TTAACTAGGCAAGTCTACTGTACTATTCTTTTCAATATTCAAAGACCTGATAATGGTATAGATTCGATTC
TTCCTGGAAGGCCATTTTCTATCAGAGACGTTAGTTTGTTTGATGGAAATGGACTTCAATGATATAAATA
ATAGGACA

XP_016551877.1 inositol monophosphatase 3 [*Capsicum annuum*]

MAQNGAVEEFLDVAVEAAKKAGEIIREGFYKTKHVEHKGVVDLVTETDKACEDLIFNHLKEHFPSHKFIG
EETTAASGNFELTDEPTWIVDPLDGTTNFVHGFPFVCVSIGLTIGKKPTVGVVYNPIIDELFTAIEGKGA
FFNGKPIKVSSQSELVKALLATEAGTKRDKLTVDATTGRINSLLFKVRSLRMCGSCALNLCGVACGRLDL
FYEHEFGGPWDVAGGAVIVKEAGGFVFDPSGSELDLTARRVAATNAHLKDAFIKALNESE-

>Ca_GPP2

TGATTAAATAATTTAATTAAGTAAAATATATATTAATTAATCATGATTAAATAACGTAAACTCTAAATTC
AAACACATGGATATGAAAATTTTATCTCTAAAGTTGATAACATATGACTTATAAAGAAATATTAGACCTG
AATGTAACATTATTCAAAAGGACAATTTTGCAAAAACTATATTTGCAAAACTTTTTAATTATAAGAATAA
CATCTGAATGACCTAAAATAAAAAGTTTCATTTGCATAAATCAAATTCACCAATTTCTCTCTTTTCCAAA
AATAAAAAATAAAAAATTGAATGGCTCAACATGTTTCACTAGAAGAATTCTTGGTTATTGCAATTGATGC
AGCAAAGAGAGCTGGAGAAATAATTTGCAAAGGATTTTATGAGACTAAACATGTGGTGCACAAAGGCCAG
GTGGATTTGGTCACGGAGACAGACAAGGTGTGTGAAGATCTCATATTCAATCATCTGAAGCAACATTTTC
CGACTCACAAGTTCATAGGTGAAGAAACTTCTGCTACTACCGGAGATTTTGAGCTAACTGACGAACCAAC
ATGGATAGTTGATCCGGTTGATGGCACAACTAATTTTGTGCATGGGTTTCCTTCTGTCTGTGTCTCGATT
GGTCTCACGATTGGGAAGATTCCAACAGTTGGCGTTGTTTATGACCCAATCATCGACGAGCTTTTCACTG
GAATTGACGGGAAAGGGGCATTTCTCAACGGGAAGCCTATCAAAGTATCTTCTCAGTCCGAACTTGTGAA
GTCCCTCCTCGGCACAGAGGTTGGAACTACGCGGGATAACTTAACTGTAGAAGCTACAACGAAGAGAATC
AATAACCTGCTTTTCAAGGTTAGATCGCTCCGAATGTGTGGTTCGTGTGCACTTGATCTCTGTTGGGTAG
CATGTGGAAGGCTTGAACTCTTCTATTTAATTGGATTTGGCGGCCCTTGGGATGTTGCAGGTGGCGCAGT
AATAGTTAAGGAAGCGGGAGGCATCCTGTTTGATCCATCTGGTTCGGAATTTGACATCACAGCCCAACGT
GTAGCTGCGACGAACCCTCATCTCAAGGAAGCATTTATCGAGGCCTTGCAACTATCCGAATGAGAATCAG
TTAAGTTCTTTCATTATTAGGCCAAAAGAATTCAATTCTTGTATGCATTGTTACTGATTTTCTTTTAGTA
GCTACTGTTATCTGGCTATATGGTGTAAGAAACTTTCTGTAAACAGCTCATGTGTGACATCAAAAAATGT
TTTTTTGTTTTGGTTGAAAAAAGTTAACTTTAGCTTTTGATCTTGAGTGGTAAAAGGCGCAGCGTGTGAT
GTGTGAATTAGGCGCACATTATGGGGTTCGAGCTGTGTCATAGAGAAAA

KAF3637985.1 Inositol monophosphatase 1 [*Capsicum annuum*]

MAQHVSLEEFLVIAIDAAKRAGEIICKGFYETKHVVHKGQVDLVTETDKVCEDLIFNHLKQHFPTHKFIG
EETSATTGDFELTDEPTWIVDPVDGTTNFVHGFPSVCVSIGLTIGKIPTVGVVYDPIIDELFTGIDGKGA
FLNGKPIKVSSQSELVKSLLGTEVGTTRDNLTVEATTKRINNLLFKVRSLRMCGSCALDLCWVACGRLEL
FYLIGFGGPWDVAGGAVIVKEAGGILFDPSGSEFDITAQRVAATNPHLKEAFIEALQLSE-

>Ca_GalDH

ATTTCACTTTTATTTTTATTAGTTTCACTAATAATTAATTAAATTTTTTATAATTATTTTGTAAAAATAA
AAATTTGTGTGAATATAACAAATTAAGGAATATGTTTTATGTTATTTTTCCAATAACGTATTTTTAGGAA
ATGAGTCGTTTAATTTCTGAGTAAGGCAGCACGAATTTTTTGGAATCAGTAACGTCTCTCATCCACTCAC
AATTGTCATCTTCTCAACGAAGCAGAAGACAATCTTCAGATTTCAAAAACCCTAACATTTCCATCAATGG
CGGCAGCAGCAGCTCAGACATTGCAGCTCCGACCACTTGGCAATACTGGACTTAAACTCAGCTCCGTCGG
TTTCGGCGCTTCTCCACTCGGCAAGGTTTTCGGTGATGTCTCCGAACAAGACGCCATCGCCGCCGTCCGG
GAAGCCTTTCGACGAGGCGTCAATTTCTTCGACACTTCCCCGTTTTATGGAGGAACTTTATCGGAAAAGG
TATTAGGGAGAGCGTTGAAGGCTCTTGGAGCGCCTAGAGATCAGTACATTGTGTCTACGAAATGTGGGAG
GTACAAAGAGGGATTTGATTTCTGTGCTGAGAGAGTCACTAAAAGCATTGATGAGAGCTTGGAGAGGCTG
CAGCTTGATTATGTTGATATCTTTCATTGTCATGATATTGAATTTGGGTCCCTCGATCAGATTGTGAATG
AGACGCTTCCCGCCCTTCTAAAACTGAAGCAAGCTGGAAAGATCCGTTTCATTGGTATAACCGGCCTTCC
TTTGGGGATATTCACTTATGTGCTTGATCGCGTCCCTCCAGGCACAGTTGATGTAATCCTGTCATATTGT
CACTACAGTATCAACGATTCAACTTTGGAGGATCTGTTGCCATACCTGAAGAGCAAGGGTGTGGGAGTGA
TCAGTGCTTCTCCTCTTTCAATGGGTCTTCTTACTGAGGCTGGAGGTCCGGAGTGGCACCCTGCTTCTTC
TGAACTTAAGGCTGCCTGCCGAGCTGCTGTCGATCATTGCAAAGAAAGGGGAAAGAATATCTCAAAATTA
GCCTTGCAGTACAGCTTAACAAATACTGATATTTCTACCATACTAGTGGGGATGAAGTCAGTTAAAGAGG
TGGAGGAGAATATAGCAGCTGCCCTAGAACTAGCAACGGTTGGGATGGATGAAGAAGCATTATCAGAGAT
CACAGAAATTCTGAAACCAGTTAAGAACCAGACATGGCCTAGCGGTATCCAACAACGTTGATTTTTTTGA
CAGTTCTATGAGCTGTGAAATAAGGTCTTGTGATCCCCTTTTTTTGCATGTTTATTTTCACTTGGACATC
TCATACTAATACTAGTCCAAGTTGAAATTTTATGTTGTATTCTTTGAGCTTTTACCTGTCAAAATTGAAA
ATAATGAAGAAATGTTGCATTACTGAAGTACGGGGGGCTGGTTTTCGTTTTTTATGCCTTTCTTGGAACT
GATAGAGATTTGTTGGAAGCAAATTAGATCTAA

XP_016539344.1 L-galactose dehydrogenase [*Capsicum annuum*]

MAAAAAQTLQLRPLGNTGLKLSSVGFGASPLGKVFGDVSEQDAIAAVREAFRRGVNFFDTSPFYGGTLSE
KVLGRALKALGAPRDQYIVSTKCGRYKEGFDFCAERVTKSIDESLERLQLDYVDIFHCHDIEFGSLDQIV
NETLPALLKLKQAGKIRFIGITGLPLGIFTYVLDRVPPGTVDVILSYCHYSINDSTLEDLLPYLKSKGVG
VISASPLSMGLLTEAGGPEWHPASSELKAACRAAVDHCKERGKNISKLALQYSLTNTDISTILVGMKSVK
EVEENIAAALELATVGMDEEALSEITEILKPVKNQTWPSGIQQR-

>Ca_GalLDH

TAAATTCAAATTTAGTTAAACTCCATCACGCGTCTTCTTCACTCCAATGTAAGACTTTTCGTGCCTAAAT
TCAAATTTAGTTTAACTCCATCACAAATATTGAACACGGAGTGTAGAAGTAGAACCAAATAAAAGAAAGA
AAGAGCATGGTCCCAAATTTTTCCTAAACCATAAAATCACAAAGAAATAATACTTACATTCATCATCATC
ATCATCATCATCAATCAACACTTGCACTAAAACCTCCCCAAAACTCCATCAGAACGACACCGTTTTCCAA
TGCTCCGTTCCTTCATTTCCAAACGCTCTCTCCAGTCCTTCCTCCGCTACCACCACCACCACCACCGGGC
CCACCGCCGCCCCTTCTCCACCACTCCGGGCCCACCTACCGCCGACGCTGAACTCCGTAAATACATCGGC
TACACTCTCCTCCTCGTAGCCTCCGGCGCCGCCACTTACTATTCCTTCCCATTCCCCGAAAATGCTCGCG
ACAAAAAAGCCCAATTGTTCCGTTACGCTCCGTTACCCGACGATCTCCACACCGTTACCAACTGGAGTGG
GACCCACGAGGTGCGGACCCGGACTTTCTTACAACCCGAGTCAATTGAGCAGCTCGAAGGAATAGTGAAA
GAAGGACATGAAAGGAAGCATAAGATAAGGCCAGTCGGGTCGGGTTTATCCCCGAATGGAATCGGGTTGA
CCCGAGCTGGGATGGTGAATTTGGCTTTAATGGATAAGGTATTGAGTGTTGATAAGGAGAAGAAGAGGGT
TACTGTACAAGCTGGGATTCGGGTTCAGCAACTTGTTGATGAGATTAAGGAGTATGGAATTACTCTTCAG
AATTTTGCTTCGATAAGGGAACAACAGATTGGTGGCATTGTTCAGGTTGGTGCCCATGGCACTGGTGCCA
GGTTGCCTCCAATTGATGAGCAAGTCATAAGCATGAAACTGGTTACCCCCGCCAAGGGTACAATAGAAAT
TTCAAAAGAGAAAGATCCAGAACTCTTTTATCTAGCTCGATGTGGACTTGGGGGACTTGGTGTGGTTGCA
GAAGTCACTCTTCAGTGTGTTGAGAGGCAGGAGCTTGTAGAACATACTTTCCTCTCTAACATGAAAGATA
TCAAGAAAAATCACAAGAAATTCCTATCTGAGAACAAGCATGTCAAATACTTGCACATTCCATATACTGA
TGCAGTTGTGGTTGTGACATGCAATCCTGTATCTAAGTTGCGAGGTCCACCAAAACATAAACCTATATAT
ACTACAGAAGAAGCCTTGCAGCATGTACGAGATCTCTATCAGGAGTCTCTGAAGAAGTACAGAAGTCAAG
TTGCTGCTTCTGGTTCTCCAGATGAACCCGAAGTAGATGAATTATCATTCACTGAGCTGAGAGATAAACT
ACTTGTGATGGATCCTCTCAACAAGGAACATGTAATTAAAGTCAACAAAGCTGAGGCAGAATACTGGAGG
AAATCAGAAGGATACCGAGTAGGCTGGAGTGATGAAATTCTAGGCTTCGACTGTGGTGGCCACCAGTGGG
TATCGGAGACATGTTTCCCTGCAGGAACACTGTCAAAGCCTAGCATGAAAGACCTGGAGTACATAGAGGA
ATTGATGCAACTCATTGAGAAAGAAAGTGTACCTGCCCCTGCACCTATAGAACAAAGATGGACTGCTTGC
AGCAAAAGTCAAATGAGTCCAGCTTATAGCTCCGCGGATGATGATATATTTTCATGGGTTGGAATCATTA
TGTATCTTCCAACCATGGATGCTCGGCAGAGAAAGCAAATCACCGAGGAGTTCTTCCACTACAGGCATAT
GACACAAGCACAATTGTGGGATCGTTATTCTGCTTTTGAACATTGGGCAAAGATTGAGGTTCCCAAGGAC

AAGGAAGAGCTCGCAGCTCTGCAGGCAAGGCTAAAAAAGAAATTCCCCGTGGATGCATACAATCAAGCAC
GAAATGAGCTAGACCCGAACCACATCTTATCTAATAACATGCTTGAGAAGCTCTTCCCTTCCTCTGAAGC
TCAGTAACTGGTTCTCCAGAATTCTTGGTTGGTTTACATCTCCTTATAAATTCATTTTAACGATAGTGAT
CCTCATCATCTTTACGTCTGTTTAAAAGCAGATATTTTGTGAGGAAATTTTTGTCCTGTATATTGTTGTT
TCAGAATGTTAAGTTGAAGGAATTTTTATAATAATGCTTATCATGCACTGTATATGCTTGAGGCCAGTGT
CAAGTACTGATCGAAGAATTGTTAATTTGAAACAGTCGTTATACATT

KAF3675459.1 L-galactono-1,4-lactone dehydrogenase, mitochondrial [*Capsicum annuum*]

MLRSFISKRSLQSFLRYHHHHHRAHRRPFSTTPGPPTADAELRKYIGYTLLLVASGAATYYSFPFPENAR
DKKAQLFRYAPLPDDLHTVTNWSGTHEVRTRTFLQPESIEQLEGIVKEGHERKHKIRPVGSGLSPNGIGL
TRAGMVNLALMDKVLSVDKEKKRVTVQAGIRVQQLVDEIKEYGITLQNFASIREQQIGGIVQVGAHGTGA
RLPPIDEQVISMKLVTPAKGTIEISKEKDPELFYLARCGLGGLGVVAEVTLQCVERQELVEHTFLSNMKD
IKKNHKKFLSENKHVKYLHIPYTDAVVVVTCNPVSKLRGPPKHKPIYTTEEALQHVRDLYQESLKKYRSQ
VAASGSPDEPEVDELSFTELRDKLLVMDPLNKEHVIKVNKAEAEYWRKSEGYRVGWSDEILGFDCGGHQW
VSETCFPAGTLSKPSMKDLEYIEELMQLIEKESVPAPAPIEQRWTACSKSQMSPAYSSADDDIFSWVGII
MYLPTMDARQRKQITEEFFHYRHMTQAQLWDRYSAFEHWAKIEVPKDKEELAALQARLKKKFPVDAYNQA
RNELDPNHILSNNMLEKLFPSSEAQ-

>Ca_GulLO1
AAAACTTGCTATTAAAGTAAAATAATTGATAATCAATTAATATTGTGATTTATATATAATAATTACTTAA
AAACTGCCTATTTATGTTTTATTTTTGTTTGTTTTTTCCCATTTACGTAAAAGGCCAAAATACTCCCATA
ACTCTAGTCAATCGCCTCCTCCCTCTCGGCCCCCCCCCCGCACCCACCCACCCTCCCCAACCCCCAAGT
GGACTTCCCTTCGAAGCAATATTCATCATCTTTATAAACATCCATCCTTCTTTAAGTACAATCCAAGATT
TCATTTCAAAAGAAAAATTAATGACCAATTTATTGTGGCTCTGCCGTGGCCACTACCTTGTCCTTCTATG
GATTTCGGAAGCCACACTTTTAACAATCTCCACTGCTATGCCACCACCAAATCCCCTCAAATGCAACAAC
GTTAACTCAGATTGCCAACTTTCCAATTCCTACGGAGTTTGGGGCGATAGACAAACATGTCGCGCCCCAA
ACATTGTATATCCAACAACTGAAGAAGAACTTCGTCGAGAATTAGCCAATGCAAACAAAAACAACCTTAA
AGTCAAAATCGTAACACGATTTTCACACACTATTCCTAAACTTGCATGCCCCACGAGCAACTCAAAAAAT
TATGTCTTCATAAGCACTGAAAAATATGACTCTACGGTCGATATTAACATGGAAAAACTAACTGTCACCG
CTGATGGTGGAGTTGGACTCCGAAAACTTATTGATACGATAGAAAAGGCGGGGTTGAGTTTGGTAGCGGC
TCCGTATTGGGAAGGAGTGACGGTTGGCGGCGTAATAAGTACGGGGGCTCACGGTAGTTCGTGGTGGGGC
AAAGGAGGAGCAATTCATGATCATGTTATTGGTATAAGTCTAATTGTACCAACTAATGAATCTGAGGGTT
ATTCTAAAATAATCAAATTGACACCAGAGGATCCACTTTTGAATGCTGCTAAAGTTTCTCTTGGATTGCT
TGGCATCATTTCTAAAGTGACATTTCAGTTGGAGCCAGCATTCAAGAGAAGTATAAGATTTAATTTCACA
AATGATAGTGCGATAGAAGATGAATATAAGGAACATGCAAAGAAGAATGAATTTGGCGATATTCAATGGT
ACCCTTCTAGACAAACTGCTGTCTACAGATATGACAATAGAGTCCCCTTAAATACTTCTGGCGATGGTCT
CAATGATTTTCTTGGATTTCAATCCAATCCTATTTTGCTTTCCAAGTCTGTTCGAGCCATAGAAAAGGGA
TTTGAAACCACTAGAAATGTAGGTGGAAAATGCACAATGGCAAGTTCTTTCGTGGCATACAAGAAACTGA
TAGCAAATGGATTTAAAAACAACAAATTAATCTTCACTGGTTATCCAGTAGTAGGCCATCAAGGCAAAAT
GCAAACTTCAGGTTCTTGTTTATACTCATCTCCTATAGACATCACCACCACATGTGCTTGGGATCCAAGA
ATCAATGGACTCTTCTTCTATGAATCCACAGCCATAATCCCATCTTCAAAATTCGTAGATTTCATACGTG
GTGTGAAAAATTTGCGCGATTCGGTCAAGTCAGAGAGTATGTGTGGGGTTGACCTGTATAACGGATTCCT
ATTTCGTTTTATCAAGGCCTCAGATGCATATTTAGGCCAAGGCGAAGATTCAGTGGTTGTTGATTTTAAC
TATTATCGTGCTAGCGATGCCTTAACCCCGCGACTTAACCAAGACATTTGGGATGAAATTGAGCAAATGG
CATTTGTTAAGTATGGGGCTAAGCCACATTGGGCTAAGAATAGGATTGTAGCATTTCTTGATGTGCAAAA
GAAGTATCCAAAATTCAACAAGTTTGTTGCTGCCAAGGCACAAATGGATCCCAAGAATATGTTTTCTAGT
GAATGGTCTGATGAGATATTGTTTGGAAAACAAGAAAGTTTAAAGGGTGATGGATGCGCCTTAGAAGGGC
TGTGTATTTGTTCAGAAGATAGACATTGTAGTCCCTCAAAAGGGTACTTTTGCAAGCCAGGACTTGTTTA
TCAAGAAGCACGTGTGTGTAGGTTTTCATCAACTTCCACAAGTTGAATAAACATTTTATCCTACTGAAAA
TTAATGTCCAAGATAGAACCAGTGTACCTCGTGTTTTGTCTTGATGCAAAATTTCAATTCCAGAAAAACA
AAAAAGCAATAACATGTGGCACTCGAATTGGAAGAAGCGTGAGATTTATGGCTTATTGAGGTTAATGTGT
AGAATTAAACCAGACCACATATTTTAAGTGGCTGACTGACTTATAGATTTTCCAAAATTTTAAATAGAAA
ATGTTGTAGTAATATTTTATCATGTGTTAAAATATTCAATCATGTTTTGTGAGT

XP_016582161.1 L-gulonolactone oxidase 3 [*Capsicum annuum*]

MTNLLWLCRGHYLVLLWISEATLLTISTAMPPPNPLKCNNVNSDCQLSNSYGVWGDRQTCRAPNIVYPTT
EEELRRELANANKNNLKVKIVTRFSHTIPKLACPTSNSKNYVFISTEKYDSTVDINMEKLTVTADGGVGL
RKLIDTIEKAGLSLVAAPYWEGVTVGGVISTGAHGSSWWGKGGAIHDHVIGISLIVPTNESEGYSKIIKL

TPEDPLLNAAKVSLGLLGIISKVTFQLEPAFKRSIRFNFTNDSAIEDEYKEHAKKNEFGDIQWYPSRQTA
VYRYDNRVPLNTSGDGLNDFLGFQSNPILLSKSVRAIEKGFETTRNVGGKCTMASSFVAYKKLIANGFKN
NKLIFTGYPVVGHQGKMQTSGSCLYSSPIDITTTCAWDPRINGLFFYESTAIIPSSKFVDFIRGVKNLRD
SVKSESMCGVDLYNGFLFRFIKASDAYLGQGEDSVVVDFNYYRASDALTPRLNQDIWDEIEQMAFVKYGA
KPHWAKNRIVAFLDVQKKYPKFNKFVAAKAQMDPKNMFSSEWSDEILFGKQESLKGDGCALEGLCICSED
RHCSPSKGYFCKPGLVYQEARVCRFSSTSTS-

>Ca_GulLO2

ATAATCTTATAGATATGGATTAGTGAATCCAAAGTTTGTTTATCTAAAAGTATAAACATCGGGTTCAGTA
GATTGCGAGACAAAAAACAAGGCCCACACAACAATTAGTGTTAATTGCATGTTACCCGAAAATAATTTTT
TGCAATTTCAACATACAAGCCGCATCATACATGTATTTATATTTATGTCGGCAATTCTCTATGTACAAGT
TGTAGGTTCTCTCAAATCACCAGCTACCAGTCATGGGCGGCCCCAATATCTTAAAACTAAGTTGGATTTT
CTTTATGGTTTACTTCGGGAAATGCAGTCCCCCTGAAGAACCCATAAAATGTTCATCCAAAACCAATTTG
AACTGTACCATTACAAACTCTTATGGTGCCTTCCCGGATCGGGCCGTTTGCAAGGCAGCTCAAGCTGTTT
TCCCAACCACAGAAGAAGAGCTCATTTCTGTGGTGGCAAATGCAACAAAGGAAAAGAGAAAAATGAAGGT
AGCAACTCGATTTTCTCACAGCATACCTAAATTGGTCTGTCCAGATGGTGAAGACGGATTGCTTATAAGT
ACAAAATTCCTAAACAAGATCATTAGAGTGGATCAAGAAAACATGACTATGACTGTTGAAAGTGGGGTGA
CATTGAGACAGCTGATCAGTGAGGCAGCCAAGGCAGGACTCGTCCTGCCTTACGCGCCTTACTGGTGGGG
TTTAACCATTGGTGGCCTAATTGGGACGGGTGCTCATGGAAGTACTCTTTGGGGTTTGGGAAGTTCTGTA
CATGATTACATTGTGCAACTTCGAATTGTCACACCAGCTGAAGCTGCACACAGTTATGCTAAAGTCCGTA
CATTGGAAAATGGTAACCCCGAGCTGAATGCAGCTAGAGTCTCCCTCGGCGTTCTTGGAGTTATTTCACA
GGTTACACTGAAATTAGAACGAATGTTCAAACGTTCAATTACCCTCTCAGAAAGGAATGACTCATACTTG
TCGAAAGAAGCAGCTATATTTGGGAGACAACATGAATTTGCAGATTTTACATGGTATCCTAGCCAGCACA
AAGTTGTTTATAGGATTGATGATCGAGTTCCTGCCAACACTCCTGGCAATGCCCTTAATGACTTTCTTGG
ATTTCGCTCAACAGCTTCACTCGTCCTCGCCATTTTAAGAACCACAGAGGAGACTCAAGAATCAATAAGC
GATGCTACTGCAAAATGCTCAATTGCCAAACTAACCACTTCCACACTGAAGATTGGTGCTTATGGGTTGA
CAAACAATGGTTTGGTCTTTACTGGCTATCCAGTTGTTGGGTTTCACAACCGAGTCCAAGCGTCAGGAAC
ATGCCTAGACAGCCTTGAAGATGCAAGAATCACATCTTGCCCTTGGGACCCTAGAGTTAAAGGTCTCTTC
TTTCACCAAACAACATTCAGTATTAGCTTATCCAAAGTCAAAGGCTTCATTGAAGATGTTCAAAGGCTAG
TTGTTTTGGAGCCTAGGTCACTGTGTGTGCTTGATTTATATGATGGCATTCTCATGAGGTACATTACAAC
TTCAAATGCTTACTTGGGTGAACAAGAAGATGCTTTGGATTTTGACATCACATATTACCGAAGCAAGGAC
CCAATGTCCCCTAGACTCTTTGAAGATTTCCTGGAAGAGATCGAGCAACTTGCATTCTTCAAATATGGGG
CCTTGCCACATTGGGGAAAGAATAGGAACATAGCATTTATTGGGGCAATCAACAAGTACAAAAATGCTGA
TAAATTCTTGAAAGTGAAACAAAGCTATGACCCATTAGGTTTATTTTCCAGTGAGTGGACAGATCAAGTT
TTAGGCTTAAAAGATGGGTTGACCATAGTGAAAGATGGGTGTGCTCTGGAAGGACTATGCATTTGTTCTG
AAGATATACATTGTGCTCCAAAGAAGGGTTACTTTTGTCGACCAGGCCGAATTTACAAAGATGCAAGGGT
GTGTACTCGTTTGTCGTCACGGTAAAGTCCTTATAGTACAAACTAATATCTTGATATATGATGTAAAATA
CAACAAGACCTGCAGTGATGTGCACAGGAAGGAAATAAATACACACATTAATGTAACAAGTAAACCTTAA
TATAATACACTTAAATCTATTTATACAACTGTTCTTCATAAGTTCTTATTACTTTTTTATTCAAATACTA
AAGATGGTCTATGCTCCTTTTTTTATTCAATTGAATGCACACTTTTCTTATTGCATCACGGTCATATTGG
ATAGTACTTTTGAAATTCCCATACTT

XP_047258886.1 probable L-gulonolactone oxidase 6 [*Capsicum annuum*]

MGGPNILKLSWIFFMVYFGKCSPPEEPIKCSSKTNLNCTITNSYGAFPDRAVCKAAQAVFPTTEEELISV
VANATKEKRKMKVATRFSHSIPKLVCPDGEDGLLISTKFLNKIIRVDQENMTMTVESGVTLRQLISEAAK
AGLVLPYAPYWWGLTIGGLIGTGAHGSTLWGLGSSVHDYIVQLRIVTPAEAAHSYAKVRTLENGNPELNA
ARVSLGVLGVISQVTLKLERMFKRSITLSERNDSYLSKEAAIFGRQHEFADFTWYPSQHKVVYRIDDRVP
ANTPGNALNDFLGFRSTASLVLAILRTTEETQESISDATAKCSIAKLTTSTLKIGAYGLTNNGLVFTGYP
VVGFHNRVQASGTCLDSLEDARITSCPWDPRVKGLFFHQTTFSISLSKVKGFIEDVQRLVVLEPRSLCVL
DLYDGILMRYITTSNAYLGEQEDALDFDITYYRSKDPMSPRLFEDFLEEIEQLAFFKYGALPHWGKNRNI
AFIGAINKYKNADKFLKVKQSYDPLGLFSSEWTDQVLGLKDGLTIVKDGCALEGLCICSEDIHCAPKKGY
FCRPGRIYKDARVCTRLSSR-

>Ca_MIOX1

GTAATCCAAACAAACACTTGGCTTGAATGTATAATGAATAATGAATAGAATAATAATTAAAAAAAAAAAA
AACTTAGTTGGGAGGATACAAAGTTTAGAGATTTTCAGGGAAGGACTCCAAACGTCCGAAATCGCAGCGA
AGGACTTTCCGGCTGCTATAAATACTCTCTTTTCTTTTAACCTAAAACACGCAACCTACGCATACTTTTC
TAACATATCCTCCCTATTTGATCTTTTTTATTAGTAAAGTTTAGATTTAGGTCCTTGTATTTTCGTTATT
TTTTTTTTTTCCATTCACTATGACTATCCTCATTGAGCAGCTTCCCTTTGATATCCAGGCTGACGAAACG
ACTGTCCACGGTGTGAATGAAACGGAATTGGTAGTGGATGGTGTATTTGCGGCGCCTGAAACAAATGCGT

TTGGGCATAATTTTAGGGACTACACTGCTGAAAGTGCTAGGCAAGAAGGGGTAGAAAACTTTTACAAGAT
CAATCACCTAAATCAGACTTTTGACTTCGTTAAAAAGATGAGGGCTAAATATGCAAAATTGGACAAGGCT
GAAATGAGCATCTGGCAATGCTGTGAACTACTGAATGATGTAGTTGATGACAGTGACCCTGACTTGGATG
AACCCCAAATTCAGCATTTGTTGCAAAGTGCTGAAGCCATTAGGAAAGATTATCCTGATGAAGACTGGCT
TCATTTGACTGCCCTTATTCATGATTTGGGGAAAGTGCTTCTTCTTCCTAGCTTTGGAGAGCTGCCTCAA
TGGGCTGTTGTTGGTGACACATTCCCTGTTGGCTGTGCTTTCCATGACTCTATTGTTCACTCCAAGTACT
TCAAGGAAAACCCTGACTACAATAATCCAACTTACAACAGCAAATTTGGAATCTATTCTAAGGGTTGTGG
ACTACACAACGTCATGATGTCATGGGGTCACGATGACTATATGTACATGGTTGCAAAGGAAAATGGGACA
ACGTTGCCATCAGCTGGTCTTTTCATCATTAGATATCATTCATTTTATGTCCTGCATAAATCTGGAGCTT
ATATGGAACTAATGAATAAGGAAGATAAAGAAAATCTCAAATGGCTTCATATTTTTAACAAATATGACTT
GTACAGCAAAAGCAAGGTTCAGGTTAACGTGGAAGAAGTTAAACCTTACTATATGTCTCTCGTTGAAAAG
TATTTCCCAGCGAAGCTGAAATGGTGAAGAGTTGCGTTTAGAAGAATTAACATATGGAAGAGCTGACAAT
TTAGAAAATTAAAACTAAATAAAGACCATAGCTTGTAGGGCCCGATAGTTGGATAGTTTTTCTTGTTTTT
ATCTAATCTTTATTTTCTGTCTTTTGTTTTAGTGTTTTGCAATGTAAAAATATGGTCTGGCTTAGCAATA
AAGTGGCTGTTTAAGTTGATGGTCCA

XP_016578588.2 Mio-inositol oxygenase 1 [*Capsicum annuum*]

MTILIEQLPFDIQADETTVHGVNETELVVDGVFAAPETNAFGHNFRDYTAESARQEGVENFYKINHLNQT
FDFVKKMRAKYAKLDKAEMSIWQCCELLNDVVDDSDPDLDEPQIQHLLQSAEAIRKDYPDEDWLHLTALI
HDLGKVLLLPSFGELPQWAVVGDTFPVGCAFHDSIVHSKYFKENPDYNNPTYNSKFGIYSKGCGLHNVMM
SWGHDDYMYMVAKENGTTLPSAGLFIIRYHSFYVLHKSGAYMELMNKEDKENLKWLHIFNKYDLYSKSKV
QVNVEEVKPYYMSLVEKYFPAKLKW-

>Ca_MIOX2
TTACGTCGGCATGACTCATTAAACATATCTCTTTTTTTTTAATTATCCACACTAGGCTGAGTCACCGTCC
TTAGTATTAATTAATTTACCAAAAAAGAGCTTTATTGTCTCACTATAAATAAACAAACGTTTTTATCAAT
TTTTTTAACACAAGCAAATTATCATATCTTATTACTCTTGTTTTAGTTGAGCCTTCTTTGTCAAGCAAAA
CATGACTATCCTTGTTGCCCAAACTGAGCATGGTGCAGAAGTGGGGAAAAAGAAGGGAAGTTCTAATGGT
GGATTTGCTGTGCCTGGAAACAATGCATTTGGCAACTCATTCAGGGATTATAATGCAGAAACTGAACGGC
AAAAGATTGTAACTGAACTCTATCGCCAAAGCCACATTAACCAAACATATGATTTTGTGAAAAAGATGAG
GGAAGAATATGGAAAGTTGAATAAAGTTGAGATGAGCATATGGGAATGTTGTGAACTTTTGAATGAAGTT
GTGGATGATAGTGATCCTGATTTGGATGAACCACAAATTGAGCATTTGTTGCAAACTGCTGAAGCTATTA
GAAAGGATTATCCTAATGACGATTGGTTGCACTTGACTGGTCTTATTCATGATCTTGGTAAAGTTCTTCT
ACTTCCCAGCTTTGGAGGGCTTCCTCAATGGGCTGTTGTTGGTGATACATTTCCCCTTGGCTGTGCTTTT
GATGAATCAATTGTGCTTCATGAGCAATTGAAGGAAAATCCTGATAACAGCAATCCAGCTTACAACACAA
AATATGGAGTTTATTCTGAAGGATGTGGGCTTGAAAATGTGGTGACGTCTTGGGGACATGATGACTATAT
GTACTTGGTGGCTAAGGAAAACAAAACTACTCTTCCTTCGGCTGCATTGTTCATCATCCGATACCATTCC
TTCTATCCTCTGCACAGGGGAGGGGCATATACTCACTTGATGAATAAAGAGGATCGTGAAAACCTGAAAT
GGCTTAAAATCTTCAGCAAGTATGATCTTTATAGCAAGAGCAAATTTAGGATTGATGTGGAGAAAGTGAA
ACCTTATTACATGTCTCTCATTAAGAAGTACTTTCCAGCCAAGTTGAAGTGGTGAAATCTTCCAATCAGG
GTAGCTATGAAACAAGATACTCGTGGGCTACAAAAAATAACAAGCTTCAGGAACTAGCTATGGAATTTTA
TTTGTGTTTTCTTTTTACCTTTTCCTTTTTTCTTTGGGGGGTAGGTAGGGTGAGGGATGTAGTGTTTCCT
TGTATAAGTCATGGAAGTCTATGTAATTAAAGTTTTTAATAAATAAAAGTTTCTTTGTTGAAACTGCATT
TGTTTCAACTTCTAATCATTCGGATAAATGATGGCACAAACTATATAGTTTAGTTGAGCAAACT

XP_016552521.2 Mio-inositol oxygenase 2 [*Capsicum annuum*]

MTILVAQTEHGAEVGKKKGSSNGGFAVPGNNAFGNSFRDYNAETERQKIVTELYRQSHINQTYDFVKKMR
EEYGKLNKVEMSIWECCELLNEVVDDSDPDLDEPQIEHLLQTAEAIRKDYPNDDWLHLTGLIHDLGKVLL
LPSFGGLPQWAVVGDTFPLGCAFDESIVLHEQLKENPDNSNPAYNTKYGVYSEGCGLENVVTSWGHDDYM
YLVAKENKTTLPSAALFIIRYHSFYPLHRGGAYTHLMNKEDRENLKWLKIFSKYDLYSKSKFRIDVEKVK
PYYMSLIKKYFPAKLKW-

>Ca_MIOX3
AATTTTAAAAATTTTAATGAATTACTAGATTTTCTTTTGAGGACTCCATAGACCAAATGGTTTCTTTTAA
GGACTCTAAAATATTCCAAATTTCATGGAAGGACAATTTTACTATGACTACTATAAATACCCACATTCTA
TTTTTCAATATCACATCACAAAATCTCATTTTGCTTTATTATATATCTCTTGATTAATATTTTTTATTTT
AATTGAAATTTTTTTGTTGAAATTTTTGTGAAAAAAATGACTATTCTCATTGAGCAGCCTGAGTTTGGAT
TACAAGTGGAGGAGAAAAGAGTACCATTCAATGCCAGTGAACTTATTTTGGATGGTGGATTTGTGGTACC
AAACACATTGTCTTCTAATAATGATGATGAAATATTTGAAGTGCCAAACATAAATGCATTTGGTCAATCA

TTTAGGGATTATAATGCAGAAAGTGAAAGGCAAAAGACAGTGGAGGAATTTTATAGGGTTCAACACATTA
ACCAAACATATGACTATGTAAAAAGAATGAGAGAAGAATATGGAAAATTGAACAAGATTGAAATGAGTAT
TTGGGATTGTTGTGAACTTTTGAATGATGTTATTGATGATAGTGATCCAGATTTGGATGAACCACAAATT
GAGCATTTGTTACAAACAGCTGAAGCTATTAGAAAAGATTATCCAAATGAAGATTGGCTTCATTTGACTG
GTCTTATTCATGATCTTGGGAAAGTGCTACTTCACCCAAGTTTTGGAGGGCTTCCTCAATGGGCTGTTGT
TGGAGATACATTTCCTCTTGGATGTGCTTTTGATGAATCAATTGTTCACCACAAGTATTTTAAGGAAAAT
TCAGACATCAACAACACAATTTATAACACAAAAAATGGTGTATATGAAGAAGGTTGTGGACTTGACAAAG
TAGTTATGTCATGGGGACATGATGATTATATGTACTTAGTTTCAAAGGAAAATGGAACTACTCTTCCTTC
TGCGGCTTTATTCGTAATTCGTTACCATTCTTTCTATGCATTACATAAATCAGGAGCATATATACACTTG
ATGAATGAGGAGGACAAAGAGAACATGAAGTGGCTCAACATTTTTAACAAATATGATTTATATAGCAAGA
GCAAAGTTCGAATTGATGTGGAAAAAGTCAAGCCGTACTATCTCTCTCTCATTGAAAAGTATTTCCCAAC
AAAGTTGAGATGGTGAATAAGAAGCATTTTGGTGGCAGAGTAATTTCATATAATTCATTGATGATGGAAA
AATAGCTATTTATAAATAGCTATATTTCACCTCCCTTTGTTGATTATTAAAATATTTATTTATCCTTTCG
AAATTTCTGTATTTTTTTTTTTTTCAACAAATGTATTTCTTTCTTTTCATTCATTCATGAATAAATCCCA
TTGATAGTTCGGTCATCATTTAAATTTTGATCTCGTTTTTTGAAAAAAACACAAAAATAGATTCGCGGCC
TAAATTTATT

XP_016540384.1 Mio-inositol oxygenase 4 [*Capsicum annuum*]
MTILIEQPEFGLQVEEKRVPFNASELILDGGFVVPNTLSSNNDDEIFEVPNINAFGQSFRDYNAESERQK
TVEEFYRVQHINQTYDYVKRMREEYGKLNKIEMSIWDCCELLNDVIDDSDPDLDEPQIEHLLQTAEAIRK
DYPNEDWLHLTGLIHDLGKVLLHPSFGGLPQWAVVGDTFPLGCAFDESIVHHKYFKENSDINNTIYNTKN
GVYEEGCGLDKVVMSWGHDDYMYLVSKENGTTLPSAALFVIRYHSFYALHKSGAYIHLMNEEDKENMKWL
NIFNKYDLYSKSKVRIDVEKVKPYYLSLIEKYFPTKLRW-

>Ca_MIOX4
TTTGAGGAAGGACTTTAATTTCTAGAACTTATAAATACTTAAACCTTTTTTCTTTTTATCTAAATATCCC
AACACATAACTTCCCTTCTTTTCTTGATATTAATTAAACAAAAAAATTTTAATCACCTTCTTTGCTTCTT
GATTATCCCTCTTTGCTTTGCTTTAATTACTTTTAATTCTCAAATTTGAGATTTGTTGTCCTTGTTGTAG
TTCTATTTGTTAGTTTCCTCTACTTTTTATTTTTATTTTTAATCACAAAAAATGACTATGCAAGTAGAGG
TACAAAAAATCCATGACCAATTCATGGTACCTCAAACCAATGCATTTGGCCATAATTTTAGGGACTACAA
CAACGTAGAAAATGAAAGGCAAAAAGGGGTAGAAAAGTTCTATAAAACACAACACATAAATCAAACCTAT
GAGTTTGTCAAGAAAATAAGACAAGATTATGCAAAATTGGACAAGGCAGAAATGAGCATATGGGAATGTT
GTGAATTACTAAATGATGTTGTTGATGAAAGTGATCCTGATTTAGATGAACCACAAATCCAACATTTATT
ACAAAGTGCTGAGGCAATTAGAAAAAATTATCCTAATGAAGATTGGCTTCATTTAACAGCTTTAATTCAT
GACTTAGGTAAAATAATTACTTTACCTAAGTTTGGTGGTCTACCTCAATGGGCTGTTGTTGGTGACACAT
TCCCTGTGGGATGTGCATTTGATGAATCAATTATCCACCACAAATACTTTCAAGAAAATCAAGATTTTAA
CAACCCAATTTACAACACAAAATATGGTGTATATTCTGAAAATTGTGGACTAGAAAATGTTATGATGTCA
TTTGGTCATGATGACTATATGTACATGGTTGCTAAAGAGAATGGTACAACATTGCCATCAGCTGGACTTT
TTATTATAAGGTATCATTCATTTTATCCATTGCATAAAAATGGAGCTTATAAACATTTGATGAATGAAGA
AGATGAAGACAATTTGAAGTGGCTTCATATTTTTAATAAATATGATTTATATAGCAAAAGCAAAGTTTAT
GTTAATGTGGAAGAGGTCAAGCCTTATTACATGTCTCTAATTGAAAAGTATTTTCCAGCAAAGCTGAGGT
GGTGAAAAGGAGGTGAAGTTGTGAACAAATTAAATTTACAATTGTAAGTATAATTTGTGTGTGTGGATTA
AAGGTGTATTATAGGTAATATTGAAGTAATAGTACTAAGCTAAGTTGTGCAGATTTTTCACTCTCGATGT
CGCATTCATGTCGGATTTTCCAAAATGTACACTATTTTTGGAGAATTTGATATAGACCCGCAGACATTTT
TGAAGAGTCTGAGCAACATAGGCAATAAGCACTGTTTAATAGTGA

XP_016549752.1 Mio-inositol oxygenase 5-like [*Capsicum annuum*]

MTMQVEVQKIHDQFMVPQTNAFGHNFRDYNNVENERQKGVEKFYKTQHINQTYEFVKKIRQDYAKLDKAE
MSIWECCELLNDVVDESDPDLDEPQIQHLLQSAEAIRKNYPNEDWLHLTALIHDLGKIITLPKFGGLPQW
AVVGDTFPVGCAFDESIIHHKYFQENQDFNNPIYNTKYGVYSENCGLENVMMSFGHDDYMYMVAKENGTT
LPSAGLFIIRYHSFYPLHKNGAYKHLMNEEDEDNLKWLHIFNKYDLYSKSKVYVNVEEVKPYYMSLIEKY
FPAKLRW-

>Ca_GalUR
GTAAATCATCCCTCAACAGAAATAACAATTACTCCCTATTCTCTTTACTCTTTTCTTTATTCTTTATTAT
TTTATAACACTGTCAAATCAAATCAAGATAATCAAATCAAAACGGAGGGCGTACAATATTTTTTCGTTAT
TATTTTTTTTTTTGGGCGTATAATATTTGAAAATTTAAATAATGCGGCATTTCTATGAGGTTAAAAGAAA
GGGCGGCACTGTCCTCTGCCAACAACATGAACCAACAAATCCCTATACCTGAAATTCCTCTTCCGTCCGG
CTGCCGAAGTATGCCGGTATTAGGTTTTGGCACGGCAGCAGATCCACCTGTAGAACCAAAATTGTTAGAA
AGTGCTATTCTTCAAGCAATTGAACTTGGGTATAGGCATTTTGACACAGCTGCTTTATACAATTCTGAAA

AACCAGCTGGTGAAGCTATAACTGAAGCAATTAATGGAGGATTAATTGAATCTCGTGAACAATTGTTTAT
TACTTCGAAGCTATGGTGTAGTGATGCTCATCCTCAACATGTTCTTCCTGCCCTCAACAAAACACTACAG
AATCTGAAGATGGATTATATTGATTTGTTCCTAATACACTGGCCAGTAAGTTCTAAGTCAGGAATACATG
AGTATCCAATTAAAAAAAAGGACTTTCTTCCAATGGATTTCAAGTCAGTTTGGGCAGCAATGGAAGAGTG
CCAGAAACTTGGACTCACCAAGTCCATTGGAGTTAGCAACTTTTCTTGCAAGAAGCTTGCTGATGTTCTT
GCCACTGCCCAGATTCCCCCTGCTGTTAATCAAGTAGAAGTAAATCCATGTTGGCAGCAACGGAAGCTGA
GAGATTTCTGCAAGAGAAATGGAGTTTTTGTTGTTGGATATAGTCCTTTAGGATCCATAGGTACCTTTTA
TGGAACCAACAGAGTTATGGAATCTCAAGTGCTCAAAGAAATAGCAAAGGCCAAAGGCAAGACCGTGGCG
CAGGTTGCTTTAAGATGGGGATGTGAGCAAGGCATTGGTGTGGTGGTAAAGATCTATAACAAAGAGAGAA
TGAAACAGAATCTTGAGATTTTCGACTGGTCATTAAGTGATGATGATTGCAGAAAGATTAGTAAAATCCC
ACAAAGTAGAGCTTGTCTTGGTAAAGATTACACATCTCATTATGGGCCTTACAAAACAATTGAAGAACTC
TGGGATGAAGAACTCTGATTACTTCAGTTGCCAGTATAAGCATGTTGTTCCTATAATTCTTGTTGTTGTA
TGTCTTCCTCCTAATTGCGGTAGTTGTATATCAAGATAGAAATAAGAAGGGAGAAAGATTCATCCAGTTA
CTTCTTGTGGTTGTATACGGTTCAACCTCGTTATAGTGGCAACATTTGTCTGGAAATTTTGTGGCTACTA
TATGATGTGTTGTTATATGTGTACCGATATTTGACATTTAGATCTCATTTAGCTGTTATAAGCCAAGACA

XP_016546784.2 LOW QUALITY PROTEIN: non-functional NADPH-dependent codeinone reductase 2-like [*Capsicum annuum*]

MRLKERAALSSANNMNQQIPIPEIPLPSGCRSMPVLGFGTAADPPVEPKLLESAILQAIELGYRHFDTAA
LYNSEKPAGEAITEAINGGLIESREQLFITSKLWCSDAHPQHVLPALNKTLQNLKMDYIDLFLIHWPVSS
KSGIHEYPIKKKDFLPMDFKSVWAAMEECQKLGLTKSIGVSNFSCKKLADVLATAQIPPAVNQVEVNPCW
QQRKLRDFCKRNGVFVVGYSPLGSIGTFYGTNRVMESQVLKEIAKAKGKTVAQVALRWGCEQGIGVVVKI
YNKERMKQNLEIFDWSLSDDDCRKISKIPQSRACLGKDYTSHYGPYKTIEELWDEEL-
