# Supplementary material for: Genome and Transcriptome Analyses of Genes Involved in Ascorbate Biosynthesis in Pepper Indicate Key Genes Related to Fruit Development, Stresses, and Phytohormone Exposures
Source: Plants (Basel). 2023 Sep 23;12(19):3367. doi: 10.3390/plants12193367 (PMC10574469; doi:10.3390/plants12193367)
Supplement: Supplementary file 1 [file plants-12-03367-s001.zip › Table S10.pdf]

Table S10. Means of CPM normalization values ± SD (standard deviation) of transcripts from Asc biosynthesis in pepper apical stems infected with three *Tobacco etch virus* strains (HAT, Mex21 and N) sampled at 7 and 14h days post-inoculation (dpi) (Bioproject - PRJNA476480). One-way ANOVA analysis was performed followed by Bonferroni's test, comparing the treatments in each time point with the control plants. Significant differences between treatments are highlighted by asterisk (*) at p<0,05. Up- and down-regulated genes are indicated in red and green, respectively.

	**7 dpi**				**14 dpi**			
**Genes**	**Control**	**HAT**	**Mex21**	**N**	**Control**	**HAT**	**Mex21**	**N**
PMI1	37.90±1.33	35.15±0.22	35.76±2.56	33.36±1.86	37.83±3.55	36.96±1.47	32.61±3.60	22.74±2.90*
PMI2	18.51±1.74	22.83±0.34	21.54±1.32	18.94±3.31	22.16±0.16	22.31±0.46	18.25±1.83	14.92±1.29*
PMI3	21.84±2.48	20.09±1.59	21.04±0.83	18.21±2.03	25.88±5.30	20.61±0.64	14.77±2.78*	10.68±4.23*
PMM	70.44±1.18	72.17±4.91	67.14±8.81	60.17±1.41	66.11±2.55	73.02±0.93	62.42±2.34	42.80±4.38*
GMP1	277.61±19.48	246.10±14.62	260.91±7.73	221.90±17.03*	285.81±0.28	261.39±7.05	205.02±0.64*	121.16±8.46*
GMP2	206.31±8.55	180.00±9.94	198.59±8.44	239.28±32.19	203.40±11.65	201.03±13.25	177.89±8.87	184.85±1.33
GME1	138.55±1.18	134.86±3.84	123.11±1.18	100.33±4.63*	170.56±11.81	150.14±1.11*	117.85±6.53*	63.97±0.68*
GME2	355.08±4.72	354.69±12.61	383.75±9.10	211.16±10.63*	433.41±33.13	425.47±12.20	316.94±23.38*	188.36±29.90*
GGP1	39.16±1.70	40.15±0.56	44.51±4.37	47.63±3.59	62.80±0.35	61.43±3.58	61.28±1.60	44.88±5.17*
GGP2	560.79±41.40	590.37±55.43	679.06±42.17	732.28±70.16	1456.31±100.92	1363.70±100.18	1140.81±11.30*	706.81±124.69*
GPP1	44.95±0.46	43.05±1.70	42.86±3.30	31.52±0.26*	46.97±4.18	48.99±3.15	34.15±1.08*	21.77±4.83*
GPP2	7.08±1.55	7.26±0.34	3.91±2.74	5.19±2.07	4.45±0.60	5.44±2.67	6.82±0.53	5.77±0.47
GalDH	67.82±1.00	67.22±1.10	64.08±0.90	35.67±8.89*	73.75±6.92	70.13±1.06	48.97±6.05*	34.16±6.43*
GalLDH	87.35±5.71	88.77±2.03	87.91±7.53	69.40±2.47*	103.31±1.39	94.01±2.23	80.57±4.52*	57.48±0.81*
GulLO1	3.35±0.16	3.50±0.72	4.13±0.07	3.95±0.06	4.03±0.01	3.53±0.26	2.49±0.02*	1.45±0.01*
GulLO2	11.26±0.30	11.37±0.92	11.78±0.55	12.70±1.13	10.70±2.14	10.74±0.24	10.51±2.01	6.89±0.20
MIOX1	42.44±4.34	44.06±0.38	46.63±8.79	37.02±1.76	16.29±3.44	15.01±0.73	15.02±1.25	8.14±0.93
MIOX2	0.47±0.04	0.55±0.02	0.38±0.43	0.85±0.31	0.64±0.21	0.28±0.05	0.48±0.18	0.38±0.03
MIOX3	63.84±0.40	65.67±10.23	49.31±34.90	9.59±0.50*	6.13±1.25	5.74±1.93	4.67±0.36	16.08±0.24
MIOX4	2.95±0.53	3.37±0.94	2.98±0.52	3.28±0.12	3.35±0.16	3.41±0.43	3.85±0.30	2.81±0.13
GalUR	3.10±0.51	3.35±0.06	3.37±0.19	2.87±0.04	3.41±0.38	3.73±0.13	3.41±0.32	1.81±0.41*
