# Supplementary material for: Genome and Transcriptome Analyses of Genes Involved in Ascorbate Biosynthesis in Pepper Indicate Key Genes Related to Fruit Development, Stresses, and Phytohormone Exposures
Source: Plants (Basel). 2023 Sep 23;12(19):3367. doi: 10.3390/plants12193367 (PMC10574469; doi:10.3390/plants12193367)
Supplement: Supplementary file 1 [file plants-12-03367-s001.zip › Table S11.pdf]

Table S11. Means of CPM normalization values ± SD (standard deviation) of transcripts from Asc biosynthesis in pepper leaves treated with exogenous salicylic acid (SA), methyl jasmonate (MeJA), ethephone (ET) and abscisic acid (ABA) phytohormones sampled after 1, 3, 6, 12 and 24h (Bioproject - PRJNA634831). One-way ANOVA analysis was performed followed by Bonferroni's test, comparing the treatments in each time point with the control plants. Significant differences between treatments are highlighted by asterisk (*) at p<0,05. Up- and down-regulated genes are indicated in red and green, respectively.

	Control					SA					MeJa				
**Genes**	**1h**	**3h**	**6h**	**12h**	**24h**	**1h**	**3h**	**6h**	**12h**	**24h**	**1h**	**3h**	**6h**	**12h**	**24h**
PMI1	0.91±0.04	0.64±0.01	0.77±0.06	0.95±0.03	0.95±0.02	0.40±0.13*	0.36±0.02	0.41±0.06	1.05±0.18	0.68±0.06	0.47±0.14*	0.42±0.16	1.09±0.36	1.11±0.28	0.20±0.03*
PMI2	18.51±3.11	13.36±1.53	15.08±0.24	10.88±1.32	14.40±0.26	19.60±2.72	14.27±1.77	9.56±0.26*	14.30±0.86	19.60±2.07*	10.72±1.32*	12.83±0.98	12.10±1.36	17.09±3.60*	18.85±2.26
PMI3	10.46±0.14	6.90±1.33	15.80±1.40	13.66±0.46	5.19±0.80	7.22±1.28*	6.25±0.65	13.88±1.26	14.12±1.11	5.59±0.80	7.59±0.54*	6.53±0.40	14.16±0.52	14.11±0.47	4.14±0.05
PMM	35.23±2.78	36.45±1.11	34.10±3.04	37.56±2.25	30.87±2.13	29.91±1.74	29.72±2.86	34.15±3.31	53.14±3.54*	31.42±1.09	34.34±2.05	41.86±0.80	42.69±0.46*	47.13±5.90*	28.23±1.44
GMP1	94.71±4.37	108.19±6.78	121.46±3.58	92.12±2.10	106.03±7.45	102.28±4.65	114.66±10.41	82.48±5.54*	87.94±9.21	100.21±7.78	98.03±3.05	122.33±8.21	90.39±3.54*	75.34±2.13*	90.98±2.37
GMP2	7.73±1.36	8.03±0.55	7.66±1.06	8.78±2.03	8.75±2.78	5.28±1.24	6.59±2.20	8.08±0.69	11.78±1.77	8.32±0.93	8.81±1.34	9.37±1.39	8.30±1.22	13.55±3.25*	9.16±1.24
GME1	172.22±11.31	140.50±10.66	85.71±4.32	92.66±4.42	164.32±5.44	175.68±6.01	126.88±3.17	92.61±2.98	100.46±4.25	175.46±4.03	153.63±10.31*	119.24±2.81*	90.41±4.22	86.65±0.72	143.59±10.48*
GME2	335.37±22.12	398.12±21.76	256.45±17.28	599.35±37.77	339.91±33.41	336.75±22.29	359.16±47.26	260.72±31.04	610.68±3.81	399.33±18.33	340.45±11.48	355.33±46.01	316.82±24.66	544.58±26.05	349.05±9.96
GGP1	53.31±1.79	38.97±0.50	42.80±1.70	70.17±5.94	41.70±3.51	48.21±3.69	41.54±4.31	49.61±1.86	76.87±8.41	61.09±0.74*	36.12±1.35*	36.54±0.53	58.67±4.79*	97.28±3.23*	44.49±3.19
GGP2	455.56±20.90	293.11±15.48	90.32±6.44	246.02±12.17	694.95±5.16	686.37±11.25*	471.91±30.15*	212.39±37.25*	249.68±6.41	1475.31±15.07*	451.37±61.40	289.81±16.93	175.94±8.18	279.61±17.22	1066.49±93.00*
GPP1	36.60±1.98	30.45±0.48	22.55±0.45	25.50±1.59	33.11±2.52	29.37±3.54*	20.71±0.59*	22.54±0.92	22.11±2.59	36.27±1.65	32.07±3.08	25.65±1.10	23.44±0.76	21.75±2.27	31.13±1.83
GPP2	8.24±1.04	6.32±0.59	7.07±0.79	12.10±1.51	8.73±0.07	5.32±1.35*	4.44±1.00	5.50±0.58	6.80±0.58*	7.38±0.71	5.07±0.29*	4.83±1.17	6.12±0.56	10.50±1.03	6.47±0.40*
GalDH	40.76±2.69	40.90±2.10	35.49±2.14	57.48±0.49	30.69±1.42	31.78±5.74*	28.15±2.16*	29.65±3.18	40.84±3.79*	31.88±4.46	33.11±0.34	31.29±3.04*	32.80±1.34	46.94±3.91*	24.92±3.28
GalLDH	125.83±4.93	131.73±5.68	103.36±8.33	111.90±6.92	74.07±8.93	110.74±2.66	97.78±7.38*	112.05±5.03	138.16±3.64*	96.39±5.36*	102.13±9.38*	98.92±4.30*	122.13±4.76*	118.49±14.51	72.76±6.66
GulLO1	2.54±0.11	2.11±0.56	3.07±0.41	2.92±0.11	2.63±0.23	2.57±0.25	1.46±0.06	2.90±0.00	2.10±0.22*	2.99±0.32	1.85±0.62*	3.17±0.13*	2.49±0.24	1.96±0.01*	1.98±0.05
GulLO2	2.37±0.41	3.94±0.35	3.87±0.67	3.49±0.86	3.39±0.07	3.44±0.37	3.94±0.87	4.45±0.23	4.49±0.43	3.04±0.07	2.91±0.77	4.22±0.11	4.36±0.29	3.56±1.06	2.84±0.38
MIOX1	1.06±0.45	1.62±0.27	1.24±0.10	0.39±0.20	0.00±0.00	1.21±0.34	0.53±0.15*	2.10±0.62*	0.33±0.01	0.30±0.08	1.39±0.18	1.50±0.00	0.25±0.09*	0.15±0.15	0.17±0.07
MIOX2	0.00±0.00	0.00±0.00	0.00±0.00	0.00±0.00	0.00±0.00	0.00±0.00	0.00±0.00	0.00±0.00	0.00±0.00	0.00±0.00	0.00±0.00	0.00±0.00	0.00±0.00	0.00±0.00	0.00±0.00
MIOX3	4.42±0.57	2.07±0.40	2.21±0.11	0.48±0.39	0.40±0.28	3.84±0.42	2.39±0.46	4.37±0.16*	3.36±0.48*	2.69±0.12*	31.30±1.59*	10.09±3.16*	2.93±0.57	0.49±0.34	0.24±0.08
MIOX4	0.00±0.00	0.00±0.00	0.00±0.00	0.00±0.00	0.00±0.00	0.00±0.00	0.00±0.00	0.00±0.00	0.00±0.00	0.00±0.00	0.00±0.00	0.00±0.00	0.00±0.00	0.00±0.00	0.00±0.00
GalUR	0.47±0.13	0.68±0.05	0.33±0.10	0.71±0.07	0.66±0.17	0.37±0.00	0.48±0.10	0.46±0.13	0.56±0.08	0.63±0.01	0.50±0.02	0.40±0.07	0.49±0.09	1.04±0.35*	0.64±0.07

	ET					ABA				
**Genes**	**1h**	**3h**	**6h**	**12h**	**24h**	**1h**	**3h**	**6h**	**12h**	**24h**
PMI1	1.11±0.35	0.77±0.19	0.72±0.08	0.59±0.15	0.20±0.04*	1.26±0.15	0.43±0.09	1.36±0.08*	0.39±0.12*	0.63±0.11
PMI2	15.88±0.90	14.61±1.94	18.87±1.99	28.64±1.65*	25.92±2.58*	17.82±2.58	11.75±0.55	10.81±0.38	12.22±0.87	20.91±2.77*
PMI3	8.76±0.73	6.34±1.12	13.37±0.89*	13.22±0.90	6.26±0.27	9.14±0.77	6.05±0.44	16.16±1.49	18.06±0.53*	3.93±1.18
PMM	45.11±1.92*	50.99±2.41*	55.11±3.60*	68.40±5.03*	55.18±4.90*	31.68±1.53	39.73±2.54	41.65±2.04*	35.37±0.72	34.48±3.50
GMP1	122.13±6.90*	169.21±3.57*	141.84±3.84*	101.75±4.76	102.41±5.14	106.94±8.01	130.78±14.73*	84.36±1.12*	86.76±3.21	75.87±2.90*
GMP2	5.70±0.31	9.79±0.91	11.40±0.67	16.52±2.70*	14.89±0.15*	6.59±1.29	7.33±0.53	7.90±1.16	9.97±0.66	7.99±0.28
GME1	142.12±4.60*	132.62±3.24	113.03±2.95*	120.28±5.81*	214.16±8.14*	126.73±16.20*	106.95±6.61*	75.50±6.23	86.52±8.05	162.13±6.35
GME2	358.18±22.92	477.35±18.37*	437.75±21.12*	904.42±33.90*	722.17±14.12*	288.96±24.74	315.50±19.37*	338.03±17.43*	627.21±6.53	416.67±17.51*
GGP1	46.15±2.67	43.54±0.10	48.81±1.63	89.91±1.22*	60.12±3.79*	31.45±5.46*	36.71±7.17	52.16±2.75	55.73±2.45*	46.39±3.33
GGP2	315.49±20.40*	232.78±7.49	93.80±5.46	248.14±6.90	961.29±82.75*	414.14±51.75	257.64±29.91	120.12±5.69	155.60±14.28*	980.39±28.68*
GPP1	35.10±0.26	30.00±2.80	28.39±0.31*	37.05±3.92*	44.24±3.21*	24.27±0.63*	23.57±0.85*	20.09±1.72	18.52±1.59*	31.41±2.70
GPP2	8.88±0.82	7.13±0.18	9.64±0.61*	11.92±1.27	8.00±0.12	4.58±0.35*	4.10±0.62*	6.17±1.31	7.75±0.13*	4.20±0.77*
GalDH	39.96±2.08	41.81±3.54	43.70±4.55	61.61±2.60	42.40±3.18*	32.32±1.34*	31.17±2.45*	35.07±3.81	53.86±2.69	27.65±5.71
GalLDH	96.85±7.47*	97.81±5.92*	100.07±3.60	123.95±7.90	96.22±1.33*	101.44±7.28*	108.90±6.63*	117.14±8.06	99.03±7.25	79.85±7.51
GulLO1	2.38±0.48	1.45±0.41	2.60±0.47	1.98±0.08*	1.26±0.15*	1.62±0.16*	2.54±0.40	2.61±0.32	3.28±0.17	1.31±0.49*
GulLO2	4.84±0.85*	5.47±0.42*	7.38±0.64*	5.09±0.08*	3.22±0.51	3.64±0.81	5.28±0.91	3.58±0.23	5.48±0.69*	3.37±0.25
MIOX1	3.19±0.09*	1.37±0.14	2.96±0.16*	0.61±0.27	0.57±0.31*	1.45±0.33	0.97±0.14*	0.99±0.32	0.57±0.10	0.19±0.04
MIOX2	0.00±0.00	0.00±0.00	0.00±0.00	0.00±0.00	0.00±0.00	0.00±0.00	0.00±0.00	0.00±0.00	0.00±0.00	0.00±0.00
MIOX3	9.48±0.30*	6.85±0.79*	21.58±0.39*	9.20±0.40*	1.81±0.27	2.36±0.06	5.08±0.69*	2.00±0.53	1.25±0.15	0.45±0.29
MIOX4	0.00±0.00	0.00±0.00	0.00±0.00	0.00±0.00	0.00±0.00	0.00±0.00	0.00±0.00	0.00±0.00	0.00±0.00	0.00±0.00
GalUR	0.83±0.08*	0.28±0.13*	0.78±0.04*	0.59±0.07	0.47±0.16	0.64±0.01	0.72±0.19	0.78±0.07*	0.94±0.06	0.57±0.08
