# Supplementary material for: Genome and Transcriptome Analyses of Genes Involved in Ascorbate Biosynthesis in Pepper Indicate Key Genes Related to Fruit Development, Stresses, and Phytohormone Exposures
Source: Plants (Basel). 2023 Sep 23;12(19):3367. doi: 10.3390/plants12193367 (PMC10574469; doi:10.3390/plants12193367)
Supplement: Supplementary file 1 [file plants-12-03367-s001.zip › Table S2.pdf]

Table S2. Description of RNA-Seq experiments used to evaluate the expression of genes involved in ascorbate biosynthesis in pepper (*Capsicum annuum* L.).

**Fruit developmental stages**				
**BioProject Accession**	**Experimental design**	**Tissue**	**Biological replicates**	**Reference**
PRJNA485468	This experiment evaluated the transcriptome sequencing of fruit pericarps of two pepper varieties ‘SJ11−3’ (higher Asc content) and ‘06g19−1−1−1’ (lower Asc content) at the stages: immature green (IMG) - 20 Days After Anthesis (DAA), mature green (MG; 30 DAA), breaker (Br; 40 DAA), and mature red (MR; 50 DAA). Each sample consisted of pericarps collected and pooled from three individual plants.	Pericarp	Three	[36]
PRJNA533286	This study investigated the expression profile of fruit peels of four varieties HJ10-1, HJ11-3-1, CJ12-17-1, and 0622-1-3-2-1-3-1 exhibiting green, white, purple, and green color, respectively at 30 DAA - Days After Anthesis (commercial maturity stage), and when it turned to yellow, orange, red and, red at 50 DAA (fully mature). Each sample consisted of six fruit peels collected and pooled from three individual plants.	Peels	Three	[37]
PRJNA668052	This study sampled fruit from five different plants (California-type sweet pepper) at three different development stages: immature green (IG), breaking point (BP), and ripe red (RR). Subsequently, fruits at BP point stage were treated with nitric oxide - 5ppm (BP2+NO) and without NO (BP2–NO) for 1h, and then stored at room temperature for 3 days before sampling.	Fruits	Four - Five	[35]
**Abiotic stress**				

PRJNA525913	Pepper plants, at the six-true-leaf stage, were submitted at four abiotic stress types: heat (40ºC), cold (10 ºC), salinity (50 mL of NaCl - 400 mM) and osmotic stress (50 mL of mannitol - 400 mM). Then, the third or fourth leaves from four plants were harvested per replicate at 0, 3, 6, 12, 24, and 72h for transcriptome assays.	Leaves	Three	[38]
PRJNA646356	This investigation evaluated the transcriptomic response of two pepper cultivars: a cold-tolerant inbred line A188 and a cold-sensitive inbred line A122 grown up ~28 ºC. At five-leaf stage, leaves samples of both cultivars under cold stress (~4 ºC for 72h), and recovery (~28 ºC) were collected at 0, 1, 2, and 12h after cold treatment, and in rewarming after 1h, posterior 72 h of cold stress.	Leaves	Three	[39]
PRJNA793609	This study investigated the transcriptome of two hot pepper genotypes, one susceptible (ZHC1) and another tolerant (ZHC2) to waterlogging. Seedlings were cultivated in a plastic pot with sand at 25 ºC and normally watered. At the emergency of sixth leaf plants, pots received water until 2 cm depth above sand surface, while control plants were normally watered with 25 mL. Then, samples made up of leaves and roots from 10 plants were collected at 6 and 24h after stress, and 1h after water removal (recovery).	Roots	Three	[40]
**Biotic stress**				
PRJNA588750	Thirty-day-old pepper seedlings of two near-isogenic lines (NIL) of bell pepper leaves: one infected with bell pepper endornavirus (BPEV) and other BPEV-free were mechanically inoculated with	Leaves	Three	[41]

	PMMoV. The inoculation experiments consisted of four treatments: BPEV+/Mock, BPEV-/Mock, BPEV+/ PMMoV, and BPEV-/PMMoV. At 7 days post-inoculation, leaves of three NIL plants were collected for RNA extraction.			
PRJNA476480	In this study was evaluated the transcriptomic of pepper plants infected with three different *Tobacco etch virus* (TEV) strains: HAT, Mex21 and N which induce mild, moderate and severe symptoms disease, respectively. At the 7 to 8-leaf stage, each strain was inoculated at the two oldest true leaves along the main stem. Samples consisting of the main stem (including the apical apex) of 5 plants were collected at 7- and 14-days post-inoculation.	Stem	Two	[42]
**Phytohormones**				
PRJNA634831	Pepper plants at the 6-true-leaf stage, were sprayed with 5 mM sodium salicylate (SA), 100 μM methyl jasmonate (JA), 5 mM ethephone (ET), 100 μM abscisic acid (ABA), or distilled water (mock). Then, each treatment was separately incubated in the growth chamber to prevent contamination. Further, the third or fourth leaf was collected at 0, 1, 3, 6, 12, and 24h post-inoculation for RNA extraction. This experiment used leaves from four healthy plants for each replicate.	Leaves	Three	[43]
