# Supplementary material for: Genome and Transcriptome Analyses of Genes Involved in Ascorbate Biosynthesis in Pepper Indicate Key Genes Related to Fruit Development, Stresses, and Phytohormone Exposures
Source: Plants (Basel). 2023 Sep 23;12(19):3367. doi: 10.3390/plants12193367 (PMC10574469; doi:10.3390/plants12193367)
Supplement: Supplementary file 1 [file plants-12-03367-s001.zip › Table S3.pdf]

Table S3. Means of CPM normalization values ± SD (standard deviation) of transcripts from Asc biosynthesis in pepper fruits of two varieties, 'SJ11−3' (higher Asc content) and '06g19−1−1−1' (lower Asc content) at immature green (IG), mature green (MG), breaker (BR) and mature red (MR) developmental stages (Bioproject - PRJNA485468). One-way ANOVA analysis was performed followed by Bonferroni's test. Statistical significance between fruits at 20 DAA compared to other stages are indicated by different lowercase letters, while capital letters represent significant differences between varieties, according to Bonferroni's test ($p < 0.05$). Up- and down-regulated genes among developmental stages are indicated in red and green, respectively.

	'SJ11−3'				'06g19−1−1−1'			
**Genes**	**IG**	**MG**	**BR**	**MR**	**IG**	**MG**	**BR**	**MR**
PMI1	1.78±0.57Bb	3.39±0.36Aa	0.70±0.20Ab	0.50±0.09Ab	4.59±1.41Aa	2.91±0.63Ab	1.49±0.20Ab	1.47±0.18Ab
PMI2	123.15±10.29Ab	167.46±11.32Aa	42.02±1.35Aa	45.09±0.60Ba	122.05±6.31Aa	85.29±0.04Bb	52.94±3.92Ab	145.98±13.92Ab
PMI3	22.39±0.05Ab	22.02±0.94Ab	34.22±3.98Aa	24.40±1.31Ab	21.24±4.50Aa	21.28±2.28Aa	23.69±3.26Ba	13.06±1.36Bb
PMM	19.38±1.24Bb	23.40±1.71Ab	56.09±2.90Aa	42.77±1.70Aa	26.76±1.12Ab	23.86±2.37Ab	39.30±3.75Ba	32.37±1.77Ba
GMP1	227.09±6.41Aa	227.15±5.41Aa	107.79±7.89Bb	90.07±5.86Bb	230.38±10.08Aa	242.22±7.34Aa	131.75±7.57Ab	165.09±4.19Ab
GMP2	24.57±1.97Ba	26.19±3.83Aa	5.16±0.33Ab	4.59±0.93Ab	59.13±8.40Aa	25.55±0.49Ab	5.50±0.16Ab	4.90±0.20Ab
GME1	148.06±10.93Ab	226.86±17.20Aa	158.24±4.17Ab	118.09±0.64Aa	126.98±3.13Ba	116.08±3.53Ba	92.29±2.22Bb	127.03±4.68Aa
GME2	668.29±66.41Aa	607.75±11.87Aa	126.95±8.42Ab	33.21±0.21Ab	574.99±35.24Ba	365.02±20.04Bb	86.52±22.15Ab	87.23±12.04Ab
GGP1	55.45±2.32Bb	102.23±6.88Aa	49.14±3.73Ab	37.31±0.80Aa	65.77±2.11Aa	65.26±1.59Ba	40.99±2.15Ab	45.22±3.23Ab
GGP2	2342.33±54.88Ab	3635.60±91.40Aa	3448.98±133.76Aa	2917.63±93.81Aa	2143.17±305.15Ab	3756.60±334.37Aa	1599.64±89.42Ba	1172.80±18.78Ba
GPP1	7.71±0.51Bb	13.28±0.21Aa	17.87±0.28Aa	9.74±0.94Aa	11.00±0.47Aa	9.44±0.08Bb	12.23±0.44Ba	10.22±0.98Aa
GPP2	5.01±0.21Ab	12.48±0.27Aa	5.98±0.40Aa	2.74±0.07Aa	0.56±0.11Bb	1.18±0.23Ba	0.45±0.02Bb	0.31±0.17Bb
GalDH	15.59±0.02Ba	19.17±0.67Aa	9.76±0.22Bb	9.50±1.36Bb	28.58±4.17Aa	23.18±1.60Ab	27.95±3.82Aa	20.16±1.52Ab
GalLDH	49.69±1.27Bb	69.64±0.32Aa	45.00±1.80Aa	29.12±0.48Aa	76.96±2.25Aa	38.73±1.37Bb	10.52±0.39Bb	9.28±0.32Bb
GulLO1	0.11±0.06	0.00±0.00	0.00±0.00	0.00±0.00	0.00±0.00	0.00±0.00	0.00±0.00	0.00±0.00
GulLO2	8.30±0.54Aa	6.92±0.14Ab	6.25±0.58Ab	9.41±0.91Aa	2.09±0.41Ba	3.28±0.05Ba	0.62±0.13Bb	0.20±0.20Bb
MIOX1	0.31±0.02Ab	0.00±0.00Ab	3.40±0.94Aa	1.87±0.96Bb	0.00±0.00Ab	0.10±0.00Ab	1.96±1.16Aa	3.76±1.01Aa
MIOX2	0.00±0.00	0.00±0.00	0.00±0.00	0.00±0.00	0.00±0.00	0.00±0.00	0.00±0.00	0.00±0.00
MIOX3	4.45±0.56Aa	0.17±0.15Ab	0.12±0.01Bb	0.00±0.00Bb	4.21±0.07Aa	0.18±0.07Ab	1.08±0.11Ab	0.80±0.12Ab
MIOX4	0.00±0.00	0.00±0.00	0.00±0.00	0.00±0.00	0.00±0.00	0.00±0.00	0.00±0.00	0.00±0.00
GalUR	0.56±0.05Aa	0.23±0.07Ab	0.20±0.03Ab	0.00±0.00Bb	0.18±0.03Ba	0.21±0.00Aa	0.19±0.03Aa	0.10±0.02Aa
