# Supplementary material for: Genome and Transcriptome Analyses of Genes Involved in Ascorbate Biosynthesis in Pepper Indicate Key Genes Related to Fruit Development, Stresses, and Phytohormone Exposures
Source: Plants (Basel). 2023 Sep 23;12(19):3367. doi: 10.3390/plants12193367 (PMC10574469; doi:10.3390/plants12193367)
Supplement: Supplementary file 1 [file plants-12-03367-s001.zip › Table S4.pdf]

Table S4. Means of CPM normalization values ± SD (standard deviation) of transcripts from Asc biosynthesis in pepper fruits of four varieties, HJ10-1, HJ11-3-1, CJ12-17-1 and 0622-1-3-2-1-3-1 at 30 and 50 DAA (Bioproject - PRJNA533286). One-way ANOVA analysis was performed followed by Bonferroni's test. Statistical significance between developmental stages in each variety is indicated by asterisk (*), according to Bonferroni's test ($p < 0.05$). Up- and down-regulated genes are indicated in red and green, respectively.

	**HJ10-1**		**HJ11-3-1**		**CJ12-17-1**		**0622-1-3-2-1-3-1**	
**Genes**	**30 DAA**	**50 DAA**	**30 DAA**	**50 DAA**	**30 DAA**	**50 DAA**	**30 DAA**	**50 DAA**
PMI1	9.33±0.04	1.29±0.34*	5.08±0.71	0.75±0.25*	4.81±0.83	0.77±0.02*	1.41±0.49	0.32±0.15*
PMI2	113.76±6.40	26.31±1.68*	106.37±10.91	26.43±3.03*	92.76±1.72	16.99±0.82*	42.69±5.53	30.00±1.38*
PMI3	29.45±1.25	17.44±2.34*	38.29±2.08	16.30±2.99*	23.54±2.47	21.77±2.23	12.53±2.82	13.01±0.64
PMM	51.90±1.06	59.18±1.68*	48.98±4.17	44.04±1.21	43.89±4.26	63.46±2.19*	55.06±1.02	41.50±1.73*
GMP1	196.39±15.18	78.79±3.06*	166.53±8.99	70.05±2.27*	179.38±10.10	68.64±8.01*	100.40±4.41	105.37±17.81
GMP2	39.83±1.17	12.94±0.50*	25.26±2.32	8.10±0.48*	29.60±1.16	8.48±1.80*	20.62±3.64	5.30±1.30*
GME1	189.52±9.25	213.27±7.55*	121.27±8.95	88.71±5.39*	197.13±10.81	214.46±5.52	116.25±7.07	98.25±4.24*
GME2	316.96±4.59	27.05±4.61*	122.85±4.73	18.93±4.17*	430.33±48.19	25.32±4.13*	211.85±24.11	70.61±4.25*
GGP1	81.35±1.96	40.66±4.96*	60.52±6.66	58.46±6.06	86.15±5.11	57.95±0.82*	77.35±4.52	54.97±6.42*
GGP2	2120.75±165.58	1234.06±48.85*	1476.55±179.11	722.42±43.81*	1709.83±22.82	1343.55±62.13*	1639.15±122.84	888.20±130.25*
GPP1	19.64±0.61	18.13±1.44	12.97±1.64	23.92±1.76*	13.91±2.35	15.47±1.56	17.19±1.75	11.38±0.89*
GPP2	6.66±0.60	15.85±1.50*	2.44±0.07	5.29±1.73*	8.64±1.10	8.98±1.82	4.98±0.34	6.95±1.13
GalDH	57.60±4.00	56.85±5.02	42.89±0.05	45.53±1.45	28.82±0.97	10.82±0.35*	45.16±1.25	52.94±4.65*
GalLDH	53.36±3.96	11.04±0.26*	36.00±5.10	13.71±2.19*	35.33±2.81	3.87±0.55*	37.85±1.88	3.32±0.90*
GulLO1	0.14±0.02	0.00±0.00	0.00±0.00	0.00±0.00	0.00±0.00	0.00±0.00	0.00±0.00	0.00±0.00
GulLO2	4.81±0.07	3.71±0.12	6.48±1.15	5.34±0.32	15.90±0.84	2.02±0.36*	0.43±0.13	0.91±0.25
MIOX1	10.25±0.65	21.46±11.97*	5.76±2.08	33.20±4.32*	0.00±0.00	17.92±0.92*	0.21±0.02	0.87±0.14
MIOX2	0.00±0.00	0.00±0.00	0.00±0.00	0.00±0.00	0.00±0.00	0.00±0.00	0.00±0.00	0.00±0.00
MIOX3	0.11±0.09	0.56±0.52	16.16±11.01	2.86±0.48*	0.26±0.02	0.62±0.19	0.23±0.05	0.55±0.17
MIOX4	0.00±0.00	0.00±0.00	0.00±0.00	0.00±0.00	0.00±0.00	0.00±0.00	0.00±0.00	0.00±0.00
GalUR	0.52±0.17	0.26±0.13	0.68±0.04	0.04±0.00*	0.71±0.13	0.00±0.00*	0.34±0.06	0.25±0.20
