# Supplementary material for: Genome and Transcriptome Analyses of Genes Involved in Ascorbate Biosynthesis in Pepper Indicate Key Genes Related to Fruit Development, Stresses, and Phytohormone Exposures
Source: Plants (Basel). 2023 Sep 23;12(19):3367. doi: 10.3390/plants12193367 (PMC10574469; doi:10.3390/plants12193367)
Supplement: Supplementary file 1 [file plants-12-03367-s001.zip › Table S5.pdf]

**Table S5.** Means of CPM normalization values ± SD (standard deviation) of transcripts from Asc biosynthesis in pepper fruits at immature green (IG), breaking point (BP1) and ripe red (RR) developmental stages, as well as fruits in the breaking point 2 without nitric oxide treatment (BP2–NO) and treated with 5 ppm NO (BP2+NO) for 1h (Bioproject – PRJNA668052). One-way ANOVA analysis was performed followed by Bonferroni's test as follows: BP1 and RR stages vs. IG; BP2-NO and BP2+NO treatments vs BP1 stage. Significant differences between treatments are highlighted by asterisk (*) at p<0,05. Up- and down-regulated genes are indicated in red and green, respectively.

| Genes | Immature green | BP1 | BP2-NO | BP2+NO | Red |
|---|---|---|---|---|---|
| ***PMI1*** | 0.43±0.27 | 0.33±0.06 | 0.14±0.06 | 0.18±0.04 | 0.24±0.04 |
| ***PMI2*** | 111.48±16.53 | 118.03±7.02 | 65.34±7.40* | 103.28±12.61 | 174.92±20.51* |
| ***PMI3*** | 20.43±1.12 | 23.12±1.61 | 27.67±1.67* | 25.35±2.42 | 18.02±2.01 |
| ***PMM*** | 57.92±4.76 | 59.44±1.07 | 44.89±2.34* | 50.12±2.65* | 37.59±1.80* |
| ***GMP1*** | 136.27±13.97 | 122.92±10.25 | 113.77±8.06 | 160.92±14.53* | 76.04±7.02* |
| ***GMP2*** | 68.83±3.10 | 57.69±1.93* | 47.02±4.22* | 42.82±4.81* | 57.71±4.25* |
| ***GME1*** | 135.73±20.49 | 138.99±5.45 | 123.89±6.96 | 99.11±4.19* | 160.20±5.44* |
| ***GME2*** | 125.12±13.72 | 66.18±11.83* | 57.33±10.05 | 141.55±23.93* | 36.24±4.75* |
| ***GGP1*** | 50.42±7.14 | 34.51±1.53* | 39.07±3.36 | 46.94±8.38* | 40.70±1.55* |
| ***GGP2*** | 1148.42±56.69 | 949.39±89.42* | 1012.94±16.84 | 1173.35±194.68* | 1017.33±34.27 |
| ***GPP1*** | 18.17±0.94 | 22.11±1.16* | 19.82±2.07 | 17.88±1.72* | 13.79±1.29* |
| ***GPP2*** | 6.11±0.54 | 3.97±0.62* | 7.17±0.67* | 6.72±1.03* | 3.65±1.30* |
| ***GalDH*** | 27.07±1.56 | 28.21±1.62 | 19.76±1.18* | 23.72±1.56* | 20.35±2.52* |
| ***GalLDH*** | 17.10±1.28 | 9.47±1.20* | 15.55±0.91* | 12.53±1.55* | 10.98±1.22* |
| ***GulLO1*** | 0.00±0.00 | 0.00±0.00 | 0.00±0.00 | 0.00±0.00 | 0.00±0.00 |
| ***GulLO2*** | 0.59±0.14 | 5.77±0.91* | 13.79±2.20* | 6.38±2.57 | 12.23±1.49* |
| ***MIOX1*** | 0.43±0.11 | 0.50±0.15 | 1.92±0.34* | 1.28±0.24* | 1.68±0.76* |
| ***MIOX2*** | 0.00±0.00 | 0.00±0.00 | 0.00±0.00 | 0.00±0.00 | 0.00±0.00 |
| ***MIOX3*** | 0.17±0.07 | 0.50±0.13 | 3.16±1.37* | 1.74±0.38* | 1.14±0.45* |
| ***MIOX4*** | 0.00±0.00 | 0.00±0.00 | 0.00±0.00 | 0.00±0.00 | 0.00±0.00 |
| ***GalUR*** | 0.23±0.12 | 0.14±0.02* | 0.21±0.02 | 0.12±0.02 | 0.05±0.02* |
