# Supplementary material for: Genome and Transcriptome Analyses of Genes Involved in Ascorbate Biosynthesis in Pepper Indicate Key Genes Related to Fruit Development, Stresses, and Phytohormone Exposures
Source: Plants (Basel). 2023 Sep 23;12(19):3367. doi: 10.3390/plants12193367 (PMC10574469; doi:10.3390/plants12193367)
Supplement: Supplementary file 1 [file plants-12-03367-s001.zip › Table S6.pdf]

Table S6. Means of CPM normalization values ± SD (standard deviation) of transcripts from Asc biosynthesis in pepper leaves under four types heat, cold, salinity and osmotic (mannitol) stresses at 3, 6, 12, 24 and 72h after treatments (Bioproject – PRJNA525913). One-way ANOVA analysis was performed followed by Bonferroni's test, comparing the treatments in each time point with the control plants. Significant differences between treatments are highlighted by asterisk (*) at p<0,05. Up- and down-regulated genes are indicated in red and green, respectively.

	**Control**					**Heat**					**Cold**				
**Genes**	**3h**	**6h**	**12h**	**24h**	**72h**	**3h**	**6h**	**12h**	**24h**	**72h**	**3h**	**6h**	**12h**	**24h**	**72h**
PMI1	0.60±0.15	0.83±0.11	0.95±0.10	0.89±0.07	0.27±0.01	0.24±0.09*	1.17±0.15	1.81±0.17*	1.50±0.14*	3.55±0.06*	0.59±0.00	0.70±0.19	0.39±0.22*	0.45±0.15*	0.42±0.01
PMI2	13.34±0.18	11.53±0.97	13.38±1.43	17.66±0.07	20.59±1.05	14.10±0.40	9.86±1.56	8.87±0.85*	9.55±1.05*	8.33±0.82*	14.88±0.94	16.14±2.48*	13.92±1.08	32.64±2.28*	38.87±3.01*
PMI3	6.41±0.11	15.23±0.90	14.70±1.12	5.78±0.14	5.05±1.45	7.64±0.25	12.14±2.02	12.47±1.90	11.29±0.47*	9.04±1.71*	6.89±0.33	16.55±2.29	20.80±1.53*	20.08±0.18*	15.19±0.61*
PMM	40.94±2.86	41.90±2.91	48.01±2.26	42.11±3.10	37.13±2.62	35.71±3.00	38.40±2.32	42.30±1.75	45.38±4.63	47.19±1.32*	41.50±4.29	46.67±0.37	36.80±2.67*	46.08±0.36	69.04±3.66*
GMP1	129.45±1.13	106.78±2.35	92.43±1.09	106.45±5.86	104.96±4.81	82.65±3.35*	67.32±3.73*	74.21±1.83*	78.88±7.33*	90.70±2.86*	111.71±0.87*	99.56±0.44	96.45±6.87	95.71±2.45*	71.49±5.09*
GMP2	5.18±1.12	7.71±0.66	8.19±0.23	6.85±0.83	6.70±0.52	5.25±1.12	6.51±1.80	5.49±1.31	3.96±0.47*	4.51±0.55	6.22±0.57	6.75±1.48	8.76±1.10	10.30±0.49*	23.75±1.90*
GME1	173.69±7.91	103.70±3.16	124.93±2.08	323.05±2.80	284.78±7.92	74.79±0.83*	69.27±0.94*	78.42±3.60*	175.34±0.57*	162.94±2.56*	251.24±1.65*	256.18±4.08*	236.33±10.38*	237.61±9.71*	226.67±3.87*
GME2	420.62±1.55	297.91±10.91	740.24±3.83	529.65±16.01	566.92±8.23	228.85±4.18*	190.46±0.27*	375.76±3.05*	535.71±7.10	474.66±16.70*	450.28±2.26*	531.34±24.06*	377.21±17.52*	332.42±13.36*	301.15±4.69*
GGP1	45.40±2.33	42.10±4.56	100.26±0.69	71.08±3.13	64.06±2.52	23.74±4.63*	37.26±3.42	50.80±3.06*	47.63±4.20*	49.78±2.10*	48.30±2.15	51.69±1.03*	43.56±2.71*	62.72±3.62*	86.98±3.89*
GGP2	304.75±10.65	58.65±0.24	207.34±1.23	1296.68±18.70	1647.89±12.64	224.54±5.47*	81.93±6.11	300.98±5.48*	1097.28±19.09*	705.83±15.41*	802.30±27.33*	855.93±46.94*	484.96±4.39*	2294.11±23.75*	1161.56±50.40*
GPP1	32.46±0.71	31.11±1.42	31.02±0.34	54.38±3.83	50.33±3.19	20.75±0.61*	18.38±0.34*	21.03±1.00*	29.63±0.57*	28.09±2.58*	45.27±2.95*	46.39±2.34*	39.31±0.43*	41.48±0.63*	48.79±0.31
GPP2	3.02±0.38	3.29±0.26	4.99±0.71	3.58±0.78	3.19±0.12	2.76±0.19	5.09±0.67*	8.72±0.03*	5.33±0.16*	5.22±0.34*	2.39±0.36	2.13±0.16*	1.90±0.48*	1.37±0.23*	1.65±0.10*
GalDH	77.56±3.53	70.66±1.81	98.00±6.79	95.19±5.35	84.92±1.27	32.83±0.27*	39.03±3.20*	55.49±2.91*	65.02±1.13*	71.32±5.05*	81.02±1.99	77.64±2.97	76.56±1.33*	73.23±1.67*	74.31±1.65*
GalLDH	92.21±1.05	88.32±5.49	119.00±1.59	95.35±2.71	74.51±6.15	52.88±0.76*	76.71±1.37*	81.56±1.02*	106.09±3.05*	84.35±2.76*	72.85±3.19*	80.33±4.93	76.69±3.91*	85.95±5.71*	120.03±6.02*
GulLO1	1.37±0.14	0.98±0.01	1.58±0.19	1.44±0.17	0.67±0.02	2.22±0.13	2.64±0.24*	1.77±0.06	0.73±0.07*	0.43±0.13	0.95±0.21	1.67±0.29	1.35±0.19	1.73±0.21*	0.95±0.32
GulLO2	2.32±0.32	2.24±0.25	2.31±0.16	2.50±0.37	1.25±0.28	2.49±0.08	3.63±0.01*	2.95±0.43	1.77±0.12	2.29±0.07*	2.08±0.22	1.73±0.04	2.33±0.46	2.39±0.48	0.81±0.18
MIOX1	0.38±0.07	0.26±0.02	0.23±0.07	0.13±0.07	0.18±0.08	0.39±0.07	0.28±0.09	0.18±0.01	0.52±0.02	0.17±0.00	0.12±0.04	0.51±0.19	0.41±0.19	0.27±0.22	1.22±0.19*
MIOX2	0.00±0.00	0.00±0.00	0.00±0.00	0.00±0.00	0.10±0.05	0.00±0.00	0.00±0.00	0.00±0.00	0.00±0.00	0.00±0.00	0.00±0.00	0.00±0.00	0.00±0.00	0.00±0.00	0.00±0.00
MIOX3	1.54±0.25	0.46±0.16	0.24±0.16	1.28±0.40	1.14±0.14	4.12±0.22*	4.71±1.10*	0.23±0.09	1.29±0.47	1.20±0.50	1.45±0.04	4.01±0.35*	2.57±0.81*	0.52±0.28	0.67±0.30

MIOX4	0.00±0.00	0.00±0.00	0.00±0.00	0.00±0.00	0.00±0.00	0.00±0.00	0.00±0.00	0.00±0.00	0.00±0.00	0.00±0.00	0.00±0.00	0.00±0.00	0.00±0.00	0.00±0.00	0.00±0.00
GalUR	0.32±0.06	0.64±0.07	0.18±0.03	0.13±0.01	0.44±0.11	1.07±0.23*	0.75±0.03	0.59±0.13*	0.32±0.00	0.55±0.05	0.23±0.08	0.00±0.00*	0.00±0.00	0.44±0.01*	0.15±0.01*

	**Salt**					**Mannitol**				
**Genes**	**3h**	**6h**	**12h**	**24h**	**72h**	**3h**	**6h**	**12h**	**24h**	**72h**
PMI1	0.18±0.09*	0.77±0.21	1.26±0.34	0.26±0.01*	0.40±0.06	0.54±0.20	0.48±0.21	0.78±0.21	0.59±0.19	0.38±0.00
PMI2	13.70±2.01	10.60±1.57	14.72±0.87	21.09±1.21	18.73±0.64	13.53±0.43	10.31±1.38	13.86±0.28	20.69±2.81	26.62±3.19*
PMI3	6.49±0.56	15.26±0.77	12.67±1.28	5.79±0.18	6.54±0.69	6.82±1.24	14.43±1.85	14.92±1.71	4.53±1.25	6.20±1.12
PMM	41.76±4.05	44.31±0.46	56.94±4.77*	72.55±2.18*	43.79±0.58	42.54±1.58	40.72±1.66	53.52±3.19	48.29±4.73	52.54±3.88*
GMP1	112.91±0.86*	74.51±3.28*	91.07±2.53	102.30±4.33	90.67±4.38*	141.25±1.12*	108.85±4.94	102.43±2.57	122.66±2.69*	122.09±1.02*
GMP2	7.11±0.31	8.75±1.17	10.93±1.12	24.53±0.90*	9.30±0.66	7.97±1.47*	7.71±0.59	12.75±1.17*	13.40±1.73*	11.89±0.66*
GME1	126.74±1.02*	75.04±2.63*	83.47±3.42*	114.38±4.14*	204.83±1.93*	151.69±3.14*	91.70±2.36	101.29±7.81*	176.69±8.85*	201.02±13.31*
GME2	263.94±8.99*	162.40±8.17*	412.72±7.77*	242.58±12.54*	388.14±10.85*	366.98±13.29*	207.97±3.46*	513.76±5.22*	405.59±4.17*	356.14±13.71*
GGP1	31.02±4.57*	38.95±2.03	67.99±2.50*	67.77±2.50	51.12±2.90*	40.25±4.91	46.80±2.35	76.52±0.41*	62.74±1.7*	62.15±2.07
GGP2	129.72±9.02*	43.70±3.93	222.83±4.88	689.47±2.02*	1273.99±37.62*	252.59±12.30	67.92±8.95	228.40±10.91	1076.15±47.20*	1082.55±76.64*
GPP1	27.84±1.90	22.78±1.20*	30.84±1.04	48.53±0.71*	51.04±1.82	32.73±0.86	24.83±2.01*	30.63±3.43	46.10±4.35*	54.10±3.22
GPP2	2.51±0.28	2.42±0.20	3.63±0.31*	2.41±0.51*	2.32±0.12	2.85±0.41	2.72±0.47	4.45±0.79	2.77±0.38	4.43±0.03*
GalDH	63.26±0.52*	47.09±1.08*	65.07±2.22*	56.24±3.09*	61.32±2.55*	71.42±3.64	63.01±2.28	84.77±0.84*	65.96±6.61*	58.66±5.51*
GalLDH	59.47±2.69*	74.96±4.46*	88.49±0.78*	77.29±3.40*	72.48±0.29	68.58±0.83*	81.44±0.95	79.32±0.61*	64.39±4.55*	46.06±1.27*
**GulLO1**	1.25±0.06	1.41±0.09	1.91±0.19	1.25±0.11*	1.20±0.36	1.21±0.22	1.55±0.30	1.80±0.07	1.66±0.36*	1.74±0.17*
**GulLO2**	1.85±0.52	2.00±0.35	2.67±0.24	2.65±0.46	3.70±0.25*	3.68±0.27*	1.61±0.02	3.03±0.34	2.54±0.25	2.10±0.45*
MIOX1	0.33±0.06	0.26±0.01	0.24±0.18	1.40±0.45*	1.35±0.83*	0.13±0.01	0.34±0.10	0.33±0.00	0.72±0.58	5.15±1.53*
MIOX2	0.00±0.00	0.00±0.00	0.00±0.00	0.00±0.00	0.00±0.00	0.00±0.00	0.00±0.00	0.00±0.00	0.00±0.00	0.00±0.00
MIOX3	0.29±0.16*	0.30±0.15	0.19±0.07	1.18±0.12	0.69±0.05	1.58±0.16	0.13±0.01	0.48±0.30	8.14±0.15*	4.94±0.44*
MIOX4	0.00±0.00	0.00±0.00	0.00±0.00	0.00±0.00	0.00±0.00	0.00±0.00	0.00±0.00	0.00±0.00	0.00±0.00	0.00±0.00
GalUR	0.41±0.01	0.39±0.05*	0.24±0.12	0.25±0.00	0.65±0.31	0.41±0.00	0.20±0.07*	0.49±0.06*	0.45±0.09*	0.19±0.05*
