# Supplementary material for: Genome and Transcriptome Analyses of Genes Involved in Ascorbate Biosynthesis in Pepper Indicate Key Genes Related to Fruit Development, Stresses, and Phytohormone Exposures
Source: Plants (Basel). 2023 Sep 23;12(19):3367. doi: 10.3390/plants12193367 (PMC10574469; doi:10.3390/plants12193367)
Supplement: Supplementary file 1 [file plants-12-03367-s001.zip › Table S7.pdf]

Table S7. Means of CPM normalization values ± SD (standard deviation) of transcripts from Asc biosynthesis in pepper leaves of two genotypes, a tolerant (A188) and another sensitive (A122) to cold at 1h, 2h and 12h of cold stress, and in the recovery (Bioproject - PRJNA646356). Two-way ANOVA analysis was performed followed by Tukey's test. Significant differences between control plants and the treatments are indicated by different lowercase letters, while capital letters represent significant differences between cultivars at $p<0.05$. Up- and down-regulated genes between treatments are indicated in red and green, respectively.

	A188					A122				
**Genes**	**Control**	**1h**	**2h**	**12h**	**Recovery**	**Control**	**1h**	**2h**	**12h**	**Recovery**
PMI1	0.80±0.28Aa	0.50±0.09Ba	1.38±0.19Aa	1.24±0.45Aa	0.31±0.13Aa	1.50±0.48Aa	1.01±0.17Aa	0.75±0.29Aa	1.32±0.15Aa	0.67±0.13Aa
PMI2	34.00±0.66Ab	61.30±1.47Aa	24.34±0.60Ba	27.29±0.47Aa	32.31±1.98Ab	34.44±1.86Aa	43.84±0.90Ba	33.80±0.61Aa	32.86±2.01Aa	22.66±0.46Ba
PMI3	6.09±0.34Bb	7.06±0.26Ab	6.76±0.52Ab	7.53±0.36Ab	24.92±0.54Aa	7.85±0.35Aa	7.92±1.11Aa	7.49±0.28Aa	8.67±0.55Aa	11.76±0.59Ba
PMM	36.77±2.09Aa	39.23±2.12Aa	37.35±0.54Ba	39.90±0.58Aa	27.94±1.35Aa	39.79±1.57Aa	34.23±0.70Aa	41.75±0.79Aa	29.45±0.76Bb	23.83±0.78Ab
GMP1	94.52±2.14Ab	125.71±3.41Aa	89.17±3.27Ab	87.86±3.06Ab	106.63±2.02Ab	72.96±3.82Ba	86.04±3.49Ba	79.97±1.82Aa	64.83±2.67Aa	36.75±1.80Bb
GMP2	8.63±0.48Ab	8.09±0.68Ab	9.30±1.12Ab	8.95±1.09Ab	13.65±0.68Aa	9.08±0.99Ab	10.11±0.69Ab	11.75±0.33Ab	8.46±0.15Ab	17.78±1.35Aa
GME1	182.53±2.02Ab	232.12±3.30Aa	169.74±3.37Ab	185.09±6.17Ab	318.02±7.33Aa	150.09±2.73Ba	175.65±5.72Ba	136.81±3.19Ba	136.73±5.28Ba	155.71±6.99Ba
GME2	297.55±9.09Ab	471.59±4.15Aa	244.05±3.78Bb	267.95±5.02Ba	167.38±7.72Aa	318.14±3.81Ab	439.01±9.28Aa	295.01±5.45Aa	333.89±4.69Ab	148.33±3.66Aa
GGP1	86.24±2.83Bb	142.16±1.59Aa	77.95±0.95Bb	76.56±1.93Ab	42.34±1.72Aa	98.21±1.56Ab	113.78±2.79Ba	103.11±2.06Ab	87.49±0.91Ab	39.61±2.19Aa
GGP2	2875.84±39.76Ab	4643.73±39.38Aa	2438.01±34.35Ba	2908.16±21.83Bb	250.09±5.81Ba	2535.17±12.79Bb	3749.65±26.98Ba	2931.80±11.25Aa	3667.11±35.25Aa	393.35±0.85Aa
GPP1	42.03±1.64Aa	47.98±0.55Aa	37.27±1.37Ab	38.47±1.36Aa	23.15±1.27Ab	38.94±1.30Aa	44.83±1.41Aa	38.44±0.52Aa	39.68±1.16Aa	21.34±0.66Ab
GPP2	2.77±0.35Ab	5.04±0.06Aa	2.57±0.37Ab	1.41±0.14Ab	1.05±0.16Ab	3.09±0.50Aa	4.17±0.57Aa	3.43±0.23Aa	1.78±0.12Aa	1.18±0.09Aa
GalDH	49.73±3.55Aa	46.35±2.43Aa	49.81±0.80Aa	45.50±0.90Aa	38.81±1.46Aa	40.95±3.28Aa	39.99±0.77Aa	32.21±1.51Ba	25.70±0.69Ba	24.91±1.07Aa
GalLDH	95.82±2.44Bb	124.29±0.31Aa	104.07±2.49Aa	101.80±0.71Ab	61.35±1.70Aa	120.50±1.73Aa	124.34±3.95Aa	102.45±3.11Ab	100.88±2.80Ab	62.57±4.32Ab
GulLO1	2.07±0.09Aa	1.19±0.13Ab	2.42±0.16Aa	1.66±0.12Aa	0.66±0.01Bb	1.81±0.43Aa	1.31±0.08Aa	1.62±0.13Aa	1.06±0.14Ba	0.82±0.01Aa
GulLO2	2.21±0.08Ab	2.21±0.14Ab	4.18±0.12Aa	3.66±0.30Ab	3.53±0.60Ab	2.17±0.34Ab	2.15±0.36Ab	4.20±0.04Aa	1.28±0.05Bb	2.44±0.22Ab
MIOX1	0.69±0.04Ab	0.19±0.07Bb	1.28±0.07Ba	0.57±0.11Ab	0.63±0.17Ab	3.01±0.45Aa	1.73±0.13Aa	6.24±0.49Aa	1.04±0.14Ab	1.66±0.11Aa
MIOX2	0.07±0.06Aa	0.06±0.04Aa	0.15±0.04Aa	0.08±0.03Aa	0.42±0.08Aa	0.08±0.04Aa	0.00±0.00Aa	0.11±0.03Aa	0.06±0.03Aa	0.06±0.00Ba
MIOX3	4.44±0.27Aa	0.61±0.01Ab	4.15±0.08Aa	2.86±0.17Ab	5.64±0.43Aa	1.42±0.33Ba	0.75±0.05Aa	2.01±0.43Ba	1.16±0.16Ba	2.73±0.37Ba
MIOX4	0.00±0.00	0.00±0.00	0.00±0.00	0.00±0.00	0.00±0.00	0.00±0.00	0.00±0.00	0.00±0.00	0.00±0.00	0.00±0.00
GalUR	1.11±0.19Aa	0.12±0.02Ba	0.63±0.00Aa	0.56±0.13Aa	0.73±0.04Ba	0.75±0.17Aa	0.59±0.10Aa	1.23±0.21Aa	1.03±0.08Aa	1.06±0.05Aa
