# Supplementary material for: Genome and Transcriptome Analyses of Genes Involved in Ascorbate Biosynthesis in Pepper Indicate Key Genes Related to Fruit Development, Stresses, and Phytohormone Exposures
Source: Plants (Basel). 2023 Sep 23;12(19):3367. doi: 10.3390/plants12193367 (PMC10574469; doi:10.3390/plants12193367)
Supplement: Supplementary file 1 [file plants-12-03367-s001.zip › Table S8.pdf]

Table S8. Means of CPM normalization values ± SD (standard deviation) of transcripts from Asc biosynthesis in pepper roots of two genotypes, a susceptible ('ZHC1') and another tolerant ('ZHC2') to waterlogging at 6 and 24h of waterlogging stress, and 1h post recovery (Bioproject - PRJNA793609). One-way ANOVA analysis was performed followed by Bonferroni's test. Significant differences between control plants and the treatments are indicated by different lowercase letters, while capital letters represent significant differences between cultivars at $p<0.05$. Up- and down-regulated genes between treatments are indicated in red and green, respectively.

	ZHC1				ZHC2			
**Genes**	**Control**	**6h**	**24h**	**Recovery**	**Control**	**6h**	**24h**	**Recovery**
PMI1	3.11±0.51_Ba	0.76±0.18_Ab	0.65±0.23_Ab	0.29±0.00_Ab	4.52±0.05_Aa	1.04±0.09_Ab	0.49±0.11_Ab	0.51±0.05_Ab
PMI2	21.10±2.17_Aa	21.62±0.53_Aa	15.26±2.17_Ab	14.62±0.69_Ab	22.07±2.42_Aa	15.74±3.02_Bb	19.14±0.60_Aa	15.99±0.11_Ab
PMI3	10.67±1.27_Bb	21.93±1.46_Aa	15.42±0.93_Aa	15.81±0.53_Aa	13.54±1.27_Ab	17.09±0.87_Ba	17.84±0.78_Aa	18.18±0.07_Aa
PMM	46.37±4.83_Aa	31.40±0.88_Ab	14.10±2.40_Ab	15.11±2.68_Ab	38.83±1.56_Ba	23.71±0.91_Bb	14.92±2.40_Ab	18.04±0.65_Ab
GMP1	75.65±3.69_Aa	73.62±4.69_Aa	76.37±0.64_Aa	65.90±4.56_Ab	70.61±0.77_Aa	62.14±0.81_Bb	74.19±3.70_Aa	52.86±0.04_Bb
GMP2	19.58±0.44_Aa	14.40±0.55_Ab	14.21±0.40_Ab	14.28±0.32_Ab	16.72±1.68_Ba	14.16±0.48_Ab	14.35±0.36_Ab	15.26±1.48_Aa
GME1	99.37±6.84_Aa	66.72±5.09_Ab	77.36±3.88_Ab	88.06±1.95_Aa	98.48±8.39_Aa	66.43±10.43_Ab	86.38±7.38_Aa	100.32±2.81_Aa
GME2	196.77±5.92_Aa	100.36±8.30_Ab	78.94±14.85_Ab	159.97±17.96_Ab	135.17±16.15_Ba	87.94±2.03_Ab	69.28±10.25_Ab	150.49±19.29_Aa
GGP1	63.20±6.58_Aa	21.62±1.22_Ab	28.05±2.60_Ab	34.43±9.06_Bb	64.06±3.23_Aa	32.63±1.09_Ab	36.56±4.72_Ab	67.55±4.54_Aa
GGP2	226.01±11.23_Aa	95.74±5.85_Ab	202.89±31.09_Aa	250.15±6.12_Ba	191.51±4.36_Ba	79.33±4.45_Ab	167.54±31.02_Ba	327.31±13.60_Ab
GPP1	32.94±1.83_Aa	11.97±1.48_Bb	10.55±0.96_Ab	13.18±1.54_Ab	27.17±1.74_Ba	18.48±0.12_Ab	13.27±0.20_Ab	15.53±0.53_Ab
GPP2	0.66±0.02_Aa	0.32±0.07_Ab	0.14±0.04_Ab	0.11±0.02_Ab	0.10±0.03_Ba	0.05±0.02_Ba	0.00±0.00_Bb	0.05±0.02_Aa
GalDH	32.60±1.95_Aa	15.94±2.64_Ab	6.28±0.22_Ab	7.46±0.26_Bb	31.00±0.58_Aa	17.41±1.23_Ab	8.33±1.81_Ab	13.90±3.45_Ab
GalLDH	71.43±2.92_Aa	29.60±1.79_Ab	44.32±4.61_Ab	52.10±3.10_Ab	67.87±1.56_Aa	32.00±4.08_Ab	41.18±2.49_Ab	52.53±2.59_Ab
GulLO1	5.53±0.48_Aa	2.23±0.38_Ab	1.73±0.55_Ab	1.18±0.20_Ab	5.95±0.67_Aa	3.10±0.35_Ab	1.52±0.02_Ab	1.74±0.26_Ab
GulLO2	58.36±2.06_Ab	124.49±1.19_Aa	151.75±1.57_Aa	119.01±8.17_Aa	45.89±5.25_Ab	124.21±6.97_Aa	138.50±6.76_Aa	128.20±6.93_Aa
MIOX1	1.15±0.12_Ab	10.25±0.70_Aa	2.40±0.31_Aa	1.57±0.36_Ab	1.31±0.30_Aa	2.03±0.50_Ba	1.07±0.02_Ba	1.13±0.06_Aa
MIOX2	0.00±0.00_Ab	0.25±0.02_Aa	0.77±0.03_Aa	0.29±0.32_Aa	0.00±0.00_Ab	0.13±0.00_Ab	0.42±0.00_Ba	0.14±0.05_Ab
MIOX3	3.31±0.24_Ab	5.87±0.09_Aa	4.64±0.91_Ab	4.88±1.71_Ab	3.03±1.26_Aa	2.65±2.15_Ba	1.64±0.43_Ba	1.71±0.05_Ba
MIOX4	0.00±0.00	0.00±0.00	0.00±0.00	0.00±0.00	0.00±0.00	0.00±0.00	0.00±0.00	0.00±0.00
GalUR	1.16±0.04_Ba	0.76±0.03_Ab	0.56±0.05_Ab	0.58±0.03_Ab	1.46±0.26_Aa	0.75±0.11_Ab	0.43±0.08_Ab	0.40±0.05_Ab
