# Supplementary material for: Genome and Transcriptome Analyses of Genes Involved in Ascorbate Biosynthesis in Pepper Indicate Key Genes Related to Fruit Development, Stresses, and Phytohormone Exposures
Source: Plants (Basel). 2023 Sep 23;12(19):3367. doi: 10.3390/plants12193367 (PMC10574469; doi:10.3390/plants12193367)
Supplement: Supplementary file 1 [file plants-12-03367-s001.zip › Table S9.pdf]

Table S9. Means of CPM normalization values ± SD (standard deviation) of transcripts from Asc biosynthesis in pepper leaves of two bell pepper near-isogenic lines (NIL), one infected with bell pepper endornavirus (BPEV+) and other BPEV-free, both further inoculated with PMMoV (Bioproject - PRJNA588750). One-way ANOVA analysis was performed followed by Bonferroni's test. Statistical significance between BPEV-/Mock compared to other treatments are highlighted by asterisk (*) at $p<0.05$. Up- and down-regulated genes are indicated in red and green, respectively.

Genes	BPEV-/Mock	BPEV+/Mock	BPEV-/PMMoV	BPEV+/PMMoV
PMI1	0.24±0.03	0.11±0.03	1.75±0.17*	1.37±0.14*
PMI2	9.63±1.15	10.43±0.62	11.40±1.02	5.67±0.52*
PMI3	10.32±1.70	12.55±1.35	11.85±0.34	11.11±1.31
PMM	28.66±1.33	34.66±1.35*	36.73±3.36*	41.66±3.42*
GMP1	147.26±5.68	112.69±10.79*	74.85±9.22*	86.26±5.25*
GMP2	16.43±1.85	19.20±0.51	13.79±1.67	13.53±2.01
GME1	131.40±12.07	177.87±1.28*	1746.55±26.54*	1538.31±16.05*
GME2	353.96±27.02	343.09±12.26	214.28±12.97*	145.30±14.40*
GGP1	44.16±2.69	61.09±1.32*	63.27±1.82*	61.80±3.94*
GGP2	288.10±13.57	226.18±22.22*	226.71±6.50*	167.97±3.37*
GPP1	29.27±1.76	32.57±1.31*	21.40±1.41*	19.76±1.09*
GPP2	2.73±0.06	3.11±0.03*	1.89±0.25*	1.16±0.16*
GalDH	26.63±2.77	19.34±1.39*	32.70±1.46*	35.89±1.76*
GalLDH	50.32±2.92	40.19±3.08*	43.03±1.85*	41.75±2.38*
GulLO1	1.76±0.33	0.56±0.38*	1.97±0.10	2.81±0.10*
GulLO2	6.10±0.03	2.33±0.58*	6.73±0.64	7.97±0.97*
MIOX1	0.35±0.28	0.41±0.04	0.30±0.02	1.04±0.08*
MIOX2	0.00±0.00	0.04±0.00*	0.00±0.00	0.00±0.00
MIOX3	21.43±9.63	45.85±8.54*	7.54±1.42	7.24±0.64
MIOX4	0.00±0.00	0.00±0.00	0.00±0.00	0.00±0.00
GalUR	1.24±0.25	0.86±0.11*	0.95±0.12*	1.74±0.09*
